# Transcriptome profiling shows a rapid variety-specific response in two Andigenum potato varieties under drought stress

**DOI:** 10.3389/fpls.2022.1003907

**Published:** 2022-09-27

**Authors:** Olga Patricia Ponce, Yerisf Torres, Ankush Prashar, Robin Buell, Roberto Lozano, Gisella Orjeda, Lindsey Compton

**Affiliations:** ^1^ School of Biosciences, University of Birmingham, Birmingham, United Kingdom; ^2^ Department of Plant Science, Wageningen University, Wageningen, Netherlands; ^3^ Unidad de genómica, Laboratorios de Investigación y Desarrollo (LID), Universidad Peruana Cayetano Heredia, Lima, Peru; ^4^ School of Natural and Environmental Sciences, Newcastle University, Newcastle upon Tyne, United Kingdom; ^5^ Department of Crop & Soil Sciences, Institute for Plant Breeding, Genetics & Genomics, Center for Applied Genetic Technology, University of Georgia, Athens, GA, United States; ^6^ Digital Science and Technology Department, Joyn Bio LLC, Boston, MA, United States; ^7^ Faculty of Biological Sciences, Universidad Nacional Mayor de San Marcos, Lima, Peru

**Keywords:** drought, potato, abiotic stress, rehydration, transcriptome

## Abstract

Potato is a drought-sensitive crop whose global sustainable production is threatened by alterations in water availability. Whilst ancestral *Solanum tuberosum* Andigenum landraces retain wild drought tolerance mechanisms, their molecular bases remain poorly understood. In this study, an aeroponic growth system was established to investigate stress responses in leaf and root of two Andigenum varieties with contrasting drought tolerance. Comparative transcriptome analysis revealed widespread differences in the response of the two varieties at early and late time points of exposure to drought stress and in the recovery after rewatering. Major differences in the response of the two varieties occurred at the early time point, suggesting the speed of response is crucial. In the leaves and roots of the tolerant variety, we observed rapid upregulation of ABA-related genes, which did not occur until later in the susceptible variety and indicated not only more effective ABA synthesis and mobilization, but more effective feedback regulation to limit detrimental effects of too much ABA. Roots of both varieties showed differential expression of genes involved in cell wall reinforcement and remodeling to maintain cell wall strength, hydration and growth under drought stress, including genes involved in lignification and wall expansion, though the response was stronger in the tolerant variety. Such changes in leaf and root may help to limit water losses in the tolerant variety, while limiting the reduction in photosynthetic rate. These findings provide insights into molecular bases of drought tolerance mechanisms and pave the way for their reintroduction into modern cultivars with improved resistance to drought stress and yield stability under drought conditions.

## 1 Introduction

As the human population is projected to approach 9 billion within four decades, the growing food gap necessitates at least a 50% increase in crop-based production to ensure food security (Grafton et al., 2015). Potato (*Solanum tuberosum* L.) is the fourth most important food crop after maize, wheat and rice, with an estimated annual tuber production of 370 million tonnes (FAO, 2021). The potato crop has highly desirable characteristics, including rich and balanced nutrition, high yields and adaptability to diverse cultivation environments, maintaining stable yields in marginal soil with limited labour inputs (Lutaladio and Castaldi, 2009; Scott, 2011; Zaheer and Akhtar, 2016). However, for potato as for other crops, drought presents a serious threat to food security as the climate changes, as it decreases crop growth and yield more than any other abiotic or biotic stress (George et al., 2018).

Modern potato varieties are particularly susceptible to periodic water shortages. Their shallow rooted systems have a weak soil penetration ability and poor nutrient uptake capacity, thus requiring consistent irrigation and making them especially susceptible to periodic droughts (Iwama, 2008). Drought conditions shorten the potato growth cycle, hamper growth and reduce the final tuber number (Deblonde and Ledent, 2001; Eiasu et al., 2007; Kumar et al., 2007). Low soil water potential can also reduce tuber quality by lowering dry matter concentration and increasing abundance of reducing sugars, resulting in “sugar ends” formation (Haverkort and Verhagen, 2008). A key goal is therefore the adaptation of existing potato varieties to drought conditions, including progressively more frequent and intense agricultural droughts due to the combined effects of growing evapo-transpiration demand and below-normal precipitation regimes (Monneveux et al., 2013). Without such adaptation, global yield losses are projected to range between 18% and 32%, particularly at lower latitudes (Hijmans, 2003).

Plants, including potato, have a broad range of adaptive strategies for responding to drought stress at morphological, physiological and molecular levels, enabling either drought escape or tolerance of lower water potential (Dahal et al., 2020). For example, drought tolerance is often associated with the accumulation of solutes such as sugar alcohols and proline that can decrease the leaf water potential and facilitate uptake of water (Dahal et al., 2020). At the molecular level, drought induces the expression of many genes involved in tolerance to the stress. One of the most well characterised responses to drought, and other forms of abiotic stress, involves the phytohormone abscisic acid (ABA). Synthesis of ABA induces transcriptional reprogramming leading to a variety of outcomes, including accumulation of osmo-protectants and stomatal closure (Sah et al., 2016). Its endogenous content is predominantly regulated through the oxidative cleavage of β-carotene in plastids. In this pathway, the NCED family of enzymes catalyse the rate-limiting cleavage of violaxanthin and neoxanthin *cis*-isomers to produce xanthoxin, which is exported to the cytosol and converted to ABA in a 2-step enzymatic process (Sah et al., 2016; Ali et al., 2020). While ABA is a crucial signaling molecule in the response to drought, there are many drought-responsive genes that do not respond to exogenous application of ABA, showing that there are also ABA-independent mechanisms (Dahal et al., 2020).

Several studies have taken omics-based approaches to understanding differences in responses to drought in leaf or root tissues between potato varieties with varying degrees of tolerance (Vasquez-Robinet et al., 2008; Evers et al., 2010; Zhang et al., 2014; Sprenger et al., 2016; Moon et al., 2018; Pieczynski et al., 2018; Chen et al., 2020). Of particular interest are the tetraploid Andean potato varieties, *S. tuberosum* subsp. *andigena*, that are well adapted to harsh climatic conditions (Vasquez-Robinet et al., 2008). These varieties may provide an important primary gene pool for improving the stress responses of the more widely grown potato *S. tuberosum* subsp. *tuberosum* (Sukhotu and Hosaka, 2006). Andigena landraces can more effectively maintain photosynthesis levels under prolonged drought stress compared with Tuberosum (Vasquez-Robinet et al., 2008). Moreover, the more stress tolerant Andigena landraces show key differences in leaves relating to resistance, including lower reactive oxygen species (ROS) accumulation, higher mitochondrial activity and more active chloroplast defence responses (Vasquez-Robinet et al., 2008). In this study, RNA-sequencing was performed at early and late stages of exposure to drought stress and after rewatering, in leaf and root tissues of two Andigenum varieties with contrasting drought tolerance phenotypes. The objective was to identify key genes and molecular pathways associated with tolerance to drought to help inform the breeding of new *S. tuberosum* cultivars with improved yield and quality under drought stress.

## 2 Methods

### 2.1 Plant material and stress treatment

Two CIP potato varieties of the subspecies *Andigenum* (*Solanum tuberosum* subsp. *andigena*) were employed in this study, namely “Negrita” (CIP accession number: 703671) and “Wila Huaka Lajra” (CIP accession number: 703248), which are tolerant and susceptible to drought, respectively. The two genotypes were chosen based on data from the PapaSalud project presented by Barandalla et al. (2010) at the XXIV congress of the Latin American Potato Association in Cusco, Peru. The PapaSalud project evaluated 77 native potato accessions for their level of resistance against different pests and diseases, their nutritional properties and adaptive potential for different environments to identify appropriate genotypes for sustainable agriculture. Their tolerance to abiotic stresses was also evaluated and data on drought tolerance was provided by the Neiker Institute (E. Ritter personal communication; **Supplementary Table 1**). Additional information on the two varieties covering a broad range of phenotypic traits is provided in **Supplementary Table 2**.

Plants were grown as described by Torres et al., 2013 in an aeroponic system installed in the Estación experimental Santa Ana (INIA - Huancayo) in Huancayo, Peru, where the temperature oscillation was between 6°C and 18°C. The aeroponic system was established based on Otazu (2010) (**Supplementary Figure 1A**). Briefly, it consisted of several wooden tables/boxes within a net house. The tables were covered with black plastic to avoid transmission of light underneath. The table top and plastic were perforated and the holes filled with Styrofoam leaving a space where *in vitro* plants were inserted so that the roots were in complete darkness under the table. Nutrients were prepared and delivered directly to the root system using a pump connected to a timer, allowing us to control the amount and timing of water and nutrients.

After 3 months of normal irrigation when plants were at the tuber initiation stage, the two potato varieties were exposed to hydric stress to simulate a drought condition by removal of water from the aeroponic system. Photosynthetic rate was measured using the CI-340 Handheld Photosynthesis System (CI-340) at different time points during stress and rewatering (Torres et al., 2013). Based on the patterns of photosynthetic activity described by Vasquez-Robinet et al. (2008), the time taken for the initial photosynthetic rate to decrease by 25% and 60% after removal of water from the system, defined as the early and late responses to drought, was determined by Torres et al., 2013. They also determined the time when plants recovered 80% of their initial photosynthetic rate after re-irrigation (Torres et al., 2013). The different responses were defined in the tolerant variety for the early response at 40 minutes after drought induction (T1), for the late response at 120 minutes after drought induction (T2), and for the recovery phase (T3) at 20 minutes after rewatering which took place at 190 min (**Supplementary Figure 1B**). The photosynthetic rate of the susceptible variety decreased more rapidly compared with the tolerant variety. While in the tolerant variety, the photosynthetic rate was reduced to 60% at 120 minutes, the same reduction in the susceptible variety occurred at 100 minutes. Moreover, after rewatering, the susceptible variety only recovered only ~50% of its initial photosynthetic rate (Torres et al., 2013). Leaves and roots from the two varieties were collected at the three stress time points (T1, T2, T3) and control samples (T0) were collected from normal irrigation conditions (i.e. before the stress), with samples from 3 individual plants providing biological replication in each condition (**Supplementary Figure 1B**). In total, there were 48 samples (4 ‘treatments’ (3 stress time points, one control before stress) x 2 varieties x 2 tissues x 3 biological replicates).

### 2.2 RNA extraction and sequencing

From each leaf or root sample, total RNA was extracted from 1-2g of material using Tri^®^Reagent (Sigma) followed by treatment with DNAase using the DNA-free^TM^ (Ambion) kit. Sample purity and concentration was determined by the OD260/OD280 and OD260/OD230 ratios using NanoDrop™ and sample integrity was verified with agarose gel electrophoresis. mRNA library construction and sequencing of all 48 samples was carried out at Michigan State University using the Illumina Hi-Seq™ 2000 platform in 1 x 50 nt single end mode.

### 2.3 Sample QC and read alignment

Read quality was assessed using FastQC v0.11.9. TrimGalore v0.6.5 (Krueger, 2021) was used to trim Illumina adapters and remove reads with a Phred score< 28 or length below 20 nt. Quality-filtered reads were aligned to the potato genome v6.1 (Pham et al., 2020) downloaded from SpudDB (http://spuddb.uga.edu/), using STAR v2.7.2b with default parameters (Dobin et al., 2013). Before mapping, index files were built for the potato reference genome with the option –runMode genomeGenerate using the gff3 genome annotation file. For mapping, the option –quantMode TranscriptomeSAM was used to align the reads to the genome. The number of counts per gene excluding ambiguous reads was obtained using HTSeq v0.11.0 (Anders et al., 2015) with the parameters: –stranded=no, –mode=union and –nonunique=none. Subsequent analysis was carried out using the R statistical software v3.6.3 and RStudio v.1.2.1335.

### 2.4 Differential expression analysis

Gene count normalization, sample quality assessment and differential expression analysis were performed using DESeq2 v1.26.0 (Love et al., 2014). Genes with raw counts below 10 across all samples were removed prior to downstream analysis. Outlier assessment was carried out after DESeq2 count normalization and variance-stabilizing transformation. Principal component analysis was performed on the top 1,000 most variable genes. To identify significant differentially expressed genes (DEGs) between individual stress time-points compared with the control, or between two varieties at any given time point, we used a threshold of below 0.05 for the default Benjamini-Hochberg adjusted P-values reported by DESeq2 and a threshold of >= 1 for the absolute shrunken log2-fold change (log2FC). This approach provides an overall false discovery rate (FDR) of 5%.

### 2.5 Functional enrichment analysis

The gene ontology (GO) terms associated with the annotated genes were downloaded from SpudDB (http://spuddb.uga.edu/GO). GO enrichment analysis of the DEGs was performed with the gost() function of the gprofiler2 R package v0.2.0, which uses the hypergeometric test to determine the significance of functional terms (Raudvere et al., 2019). Enriched GO terms were defined where the Bonferroni-corrected P*-*value from the hypergeometric test was below 0.05.

### 2.6 Identification of drought and rewatering responsive genes in the tolerant variety

We selected genes that were either up- or down- regulated in response to drought stress and then partially or fully recovered their expression after rewatering (“drought-responsive” genes) in three steps. First, we selected significantly up- or down- regulated genes in the early response (T1) compared with the control condition, with Benjamini-Hochberg adjusted P-value below 0.05 and no threshold for log 2-fold change. Second, we selected up (or down) regulated genes whose expression level was maintained or further increased (or decreased) at T2. Third, we selected genes whose expression changed in an opposite direction from T2 to T3 (i.e. recovered partially or completely) with a log 2-fold change difference of more than 0.5.

## 3 Results

To characterize drought-induced transcriptional changes in *S. tuberosum* subsp. *andigenum* we performed gene expression profiling using RNA-seq of leaf and root tissues of two varieties with contrasting drought tolerance phenotypes. RNA-seq reads from all 48 samples mapped well to the potato reference genome, with 82-90% of reads mapping uniquely (**Supplementary Table 3**). The responses of the stress tolerant variety Negrita and the susceptible variety Wila Huaka Lajra to water stress were compared at early (T1, 40-minutes) and late (T2, 120-minutes) time points and in a recovery time point following rewatering (T3). A false discovery rate of 5% and an absolute log 2-fold change >= 1 was used to identify DEGs in each variety that were up- or down- regulated in each time compared to the non-stressed control, or genes that were differentially expressed between varieties at a given time point.

### 3.1 Overview of drought-induced transcriptional changes in leaf and root

**Figure 1:**
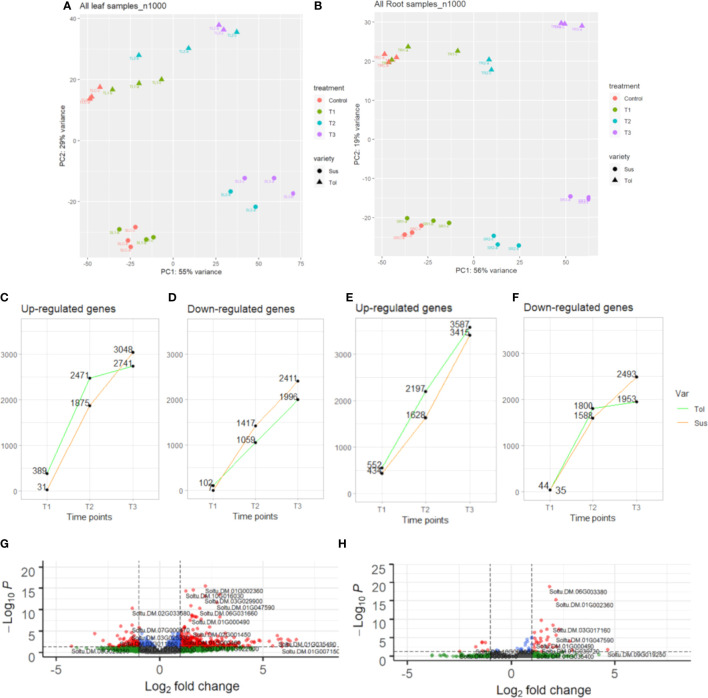
Transcriptomic overview of progressive drought response in two potato varieties. Principal component analysis (PCA) of leaf **(A)** and root **(B)** in tolerant (Tol) and susceptible (Sus) varieties in the non-stressed control and across three drought-stressed time points (early T1, late T2 and recovery after rewatering T3). The number of DEGs in each time point compared with the non-stressed control that are up-regulated in leaf **(C)** or root **(E)** or downregulated in leaf **(D)** or root **(F)**. Volcano plot showing differential expression of genes in T1 in leaf **(G)** or root **(H)** compared with the control. Vertical dashed lines indicate absolute log2FC ≥2. Horizontal dashed lines indicate padj. equal to 5%. Genes passing neither threshold are shown in grey, while non-significant genes passing the FC threshold are shown in green. In blue are genes with a small but significant fold change and in red are genes passing both thresholds.

Principal component analysis (PCA) showed clear separation between tolerant and susceptible varieties in both leaf and root, showing widespread differences in gene expression between varieties (**Figures 1A, B** **)**. In both varieties and tissues, samples were distributed according to the duration of stress treatment, with T1 samples most similar to control samples and T2 and T3 samples increasingly distant from control (**Figures 1A, B** **)**. This suggested there is a progressive response to drought stress and the observed morphological recovery in response to rewatering was not matched by a full recovery of gene expression in either variety. Also evident from the PCA plot is the relatively tight clustering in both tissues of the control and early stress (T1) samples in the susceptible compared with the tolerant variety (**Figures 1A, B** **)**. This indicated a stronger, faster response to drought stress in the tolerant variety, particularly in leaf.

**Figure 2:**
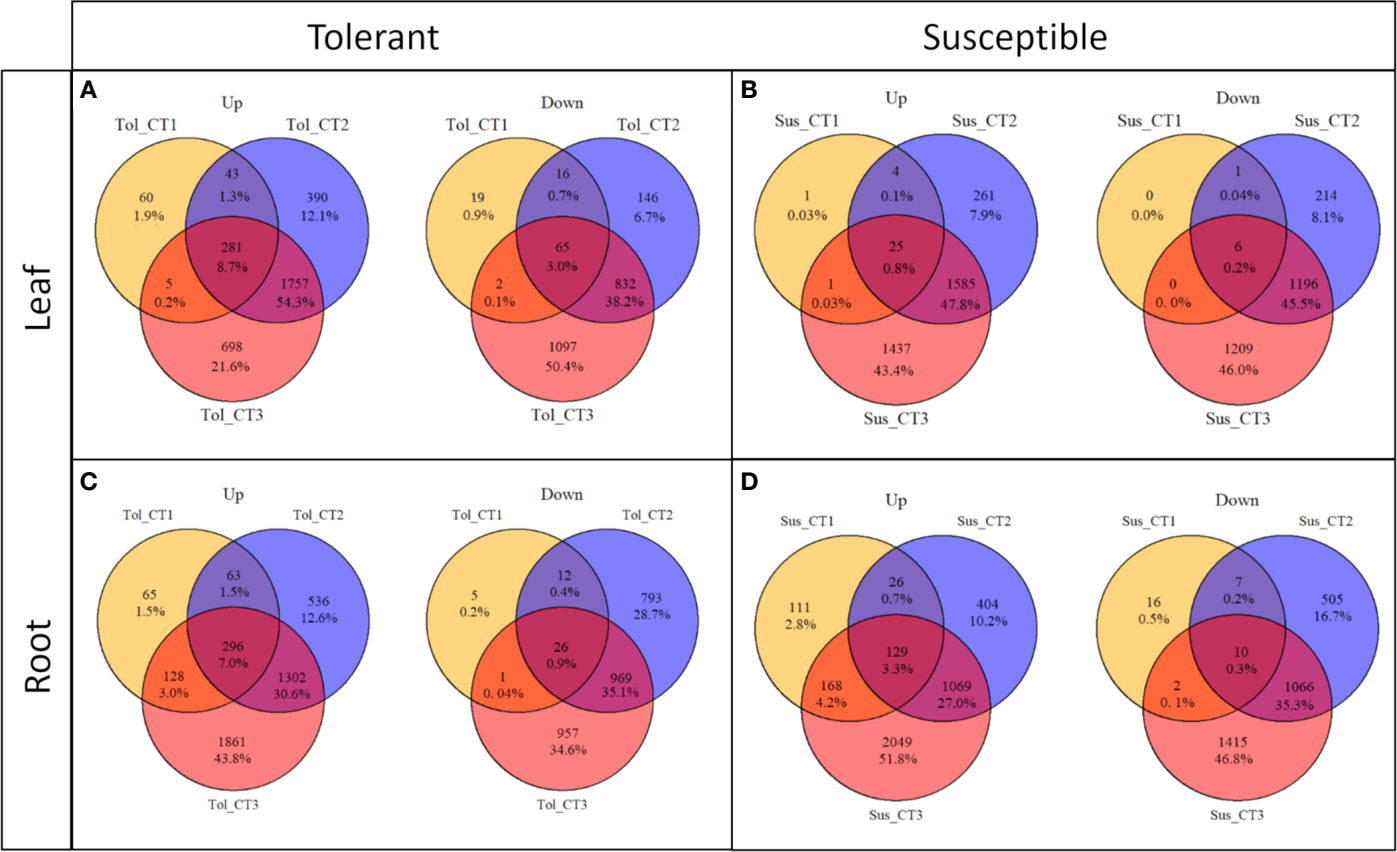
Commonalities and differences in DEGs across drought treatment time points in two potato varieties. Up and down-regulated DEGs in leaf **(A, B)** and root **(C, D)** in tolerant (Tol) and susceptible (Sus) varieties in three drought-stressed time points (early T1, late T2 and recovery after rewatering T3) compared with the non-stressed control condition.

The progressive response to drought can also be observed by looking at the differentially expressed genes in each time point compared with the non-stressed control (**Figures 1** **,** **2** **;** **Supplementary Figure 2**). Overall, there was more widespread upregulation of gene expression in response to drought stress compared with downregulation (**Figures 1C-F** **,** **3A**). For both varieties and both tissues, there was a progressive increase in the number of DEGs in each time point (**Figures 1C-F**) and a corresponding increase in the number of DEGs unique to any particular time point from T1 through to T3 (**Figures 2A-D**). In each variety, most genes differentially expressed at the early time point continued to be differentially expressed at the later time point(s), suggesting a sustained response to stress in both tissues (**Figures 2A-D**). Only a very small proportion of genes were differentially expressed across all three time points (**Figures 2A-D**).

**Figure 3:**
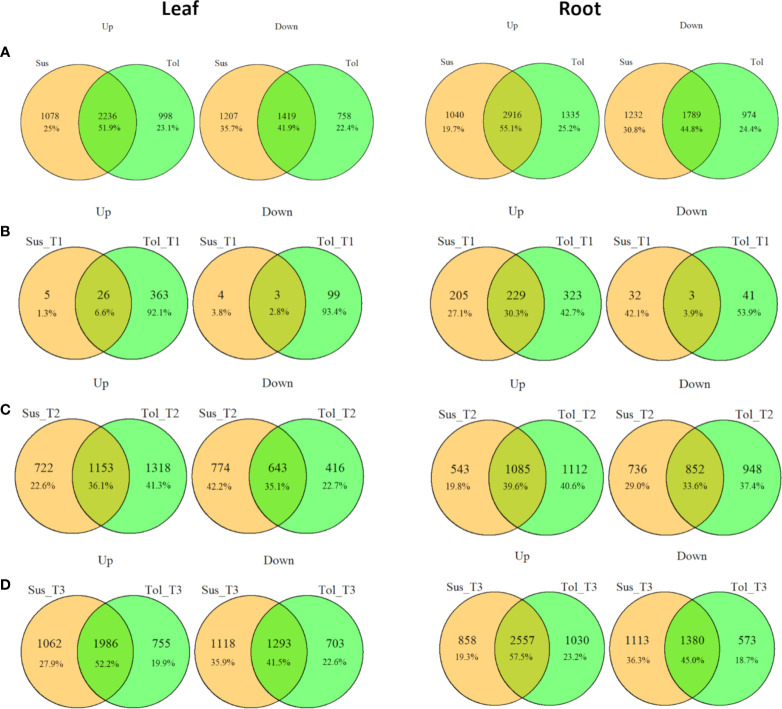
Commonalities and differences in drought-responsive DEGs between tolerant and susceptible varieties. Overlap between up- and down- regulated DEGs in leaf and root in tolerant (Tol) and susceptible (Sus) varieties in at least one drought-stressed time point (T1-T3) **(A)**, or in each of the early T1 **(B)**, late T2 **(C)** or recovery after rewatering T3 **(D)** time points compared with the non-stressed control condition.

Leaves of the tolerant variety responded more rapidly to drought stress than susceptible leaves, with 389 genes upregulated compared to only 31 in the susceptible, and 102 genes downregulated compared to 7 in the susceptible at the early time point (**Figures 1C, D** **,** **3B**). The same but less pronounced pattern was also observed in root (**Figures 1E, F** **,** **3B**). This suggested the speed of response to stress could be a crucial difference between the two varieties. The rapid response in the leaves of the tolerant variety could also be seen in the volcano plots (**Figures 1G, H** **;** **Supplementary Figure 2**), which showed more upregulated genes in the tolerant variety and more genes with a larger log2 fold change, including some genes with fold changes greater than 5.

While the early response to drought stress was largely unique to the tolerant variety, particularly in leaf, the response of the two varieties became more similar across the stress time points (**Figures 3B-D**). In the late stress response (T2), the tolerant variety continued to upregulate more genes than the susceptible variety in both tissues (**Figures 1C, E** **,** **3C**). The opposite pattern was observed for downregulated genes in leaves, with the susceptible variety downregulating more genes than the tolerant variety at T2, with this trend continuing into the recovery phase, T3 **(** **Figures 1D** **,** **3C, D** **)**. The delayed response in the leaves and roots of the susceptible variety can also be observed as an increase in the proportion of genes that were uniquely differentially expressed in T3 compared with the tolerant variety (**Figure 2**). Meanwhile, there was some indication that the tolerant variety could recover its gene expression more effectively in response to rewatering compared with the susceptible variety. This can be seen by a drop-off in the rate of gene upregulation in T3 in leaf tissue of the tolerant variety (**Figure 1C**), while the susceptible variety had more than double the number of upregulated genes unique to the T3 phase (1,437 genes) compared with the tolerant variety (698 genes) (**Figures 2A, B** **)**.

Similarly, in roots, the susceptible variety downregulated more genes in the recovery phase, T3, compared with the tolerant variety (**Figures 1F** **,** **3D**). A higher proportion of these downregulated genes in the susceptible variety (46.8%) were uniquely downregulated in the recovery phase compared with the tolerant (34.6%) (**Figures 2C, D** **)**. Meanwhile, in the tolerant variety, there was a drop-off in the rate of gene downregulation in T3 compared with T2 (**Figure 1F**).

### 3.2 Common, variety-specific and tissue-specific responses to drought stress

We identified DEGs that were up- or down- regulated in each variety compared with the non-stressed control condition and performed a gene ontology enrichment analysis to identify the functional roles of DEGs responding to drought and rewatering and the variety-specific responses that may be related to the tolerance phenotype (**Supplementary Figure 3**).

#### 3.2.1 Early responses to drought stress

Consistent with the higher number of DEGs observed in the tolerant leaf or root in the early response to drought, there were more enriched GO terms unique to the tolerant variety, particularly in leaf. Most of the enriched terms exclusive to the tolerant variety in leaves were processes related to osmotic or abiotic stress, including the responses to ABA, regulation of stomatal movement and responses to chitin. Notably, these terms were also enriched for upregulated genes in the roots of both varieties but were not enriched in susceptible leaf (**Supplementary Figure 3A**). Meanwhile, genes downregulated in tolerant leaves were enriched for seven GO terms relating to DNA replication, negative regulation of transcription factor activity and cell division, likely reflecting a generalized shut down in growth occurring in leaf, not observed in the susceptible variety until T2 **(** **Supplementary Figures 3A, B** **)**.

**Figure 4:**
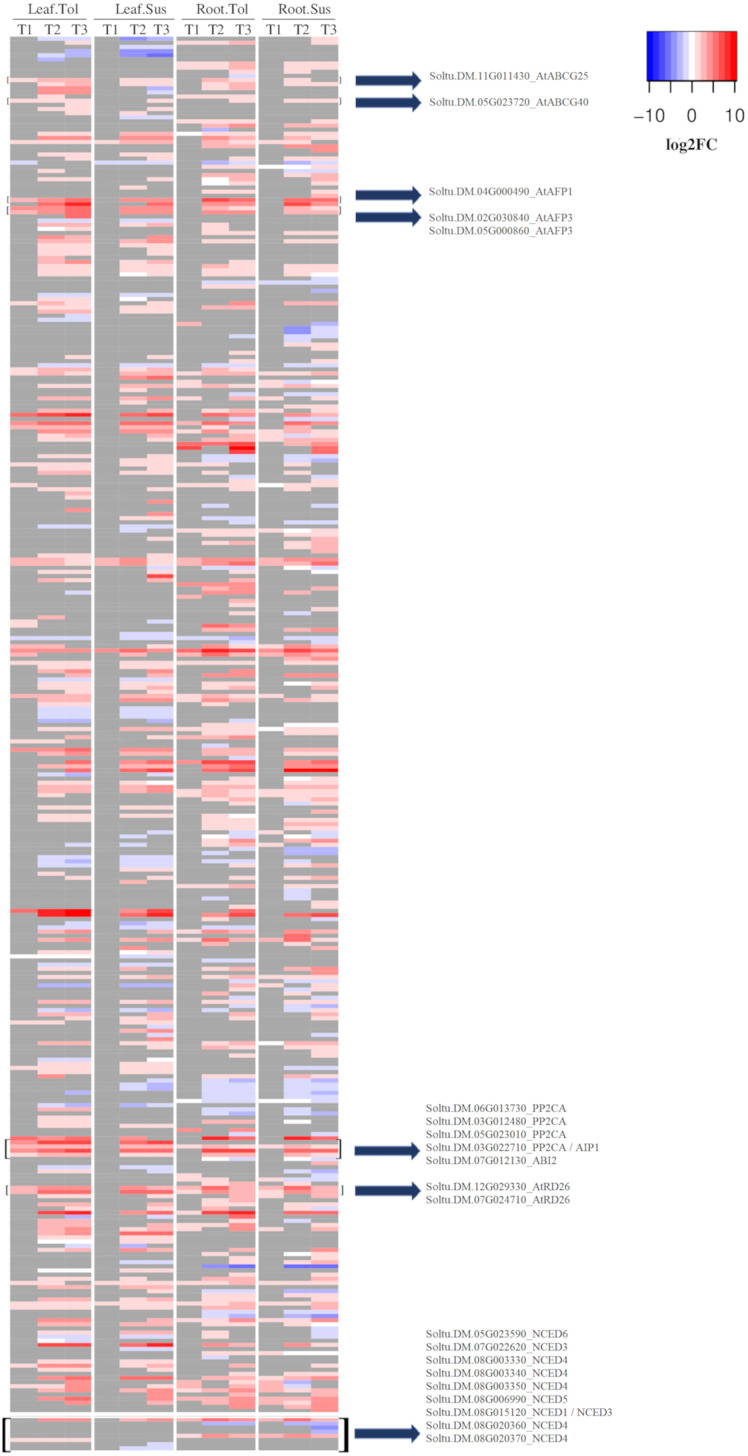
Heatmap of the log2 fold change for DEGs related to the ABA response. Shown is the log2FC compared to the non-stressed control for all DEGs annotated with GO:0009737 and all *NCED* genes. In grey are genes with no significant change in the respective time point (early T1, late T2, and recovery T3), tissue, or variety.

Within the response to ABA (GO:0009737), there were 39 upregulated DEGs in leaves of the tolerant variety compared with only 5 in susceptible leaves (**Figure 4**). These included 5 genes encoding PP2C proteins, two ABA transporters (Soltu.DM.11G011430-AtABCG25, Soltu.DM.05G023720-AtABCG40), 3 ABI five binding proteins (Soltu.DM.04G000490, Soltu.DM.02G030840, Soltu.DM.05G000860), and 2 AtRD26 proteins (Soltu.DM.12G029330, Soltu.DM.07G024710), which were not upregulated in the susceptible leaf in T1. Two of these PP2Cs were among the 20 most upregulated DEGs in only the tolerant leaf (**Supplementary Table 4B**). Most of the genes that responded to ABA early in the tolerant variety only began to be upregulated in the susceptible leaves during the late response to drought (T2) (**Figure 4**). However, there were some commonalities in the early response of leaf in both varieties. In leaf, though not in root, the upregulated DEGs in both varieties were enriched for the process of cell wall modification (GO:0009827, **Supplementary Figure 3A**). Six genes encoding plant invertase/pectin methylesterase inhibitor superfamily proteins were upregulated to similar levels in both varieties, though only three were significant DEGs in the susceptible. However, by T2, seven genes coding for these proteins were DE in both varieties with a high log2FC (**Supplementary Table 5**).

While there was very little similarity between the two varieties for gene enrichment in leaves, in root there were 21 common enriched GO terms for upregulated genes (**Supplementary Figure 3A**). Although the response to ABA term was enriched in both varieties (GO:0009737), it also contained many DEGs specific for the tolerant root, which barely overlapped with DEGs specific to the tolerant leaf (**Supplementary Table 6**). These included 3 ABCG11 transporters (for cutin transport), 2 ABCG40 transporters (for ABA transport), 1 raffinose synthase family protein, and most highly upregulated were 1 galactinol synthase (Soltu.DM.02G006360) and 2 MYB domain proteins (Soltu.DM.05G023310, Soltu.DM.12G001820) (**Supplementary Table 6**). These 2 last DEGs were among the top 20 most upregulated genes in only the tolerant root (**Supplementary Table 4**).

**Figure 5:**
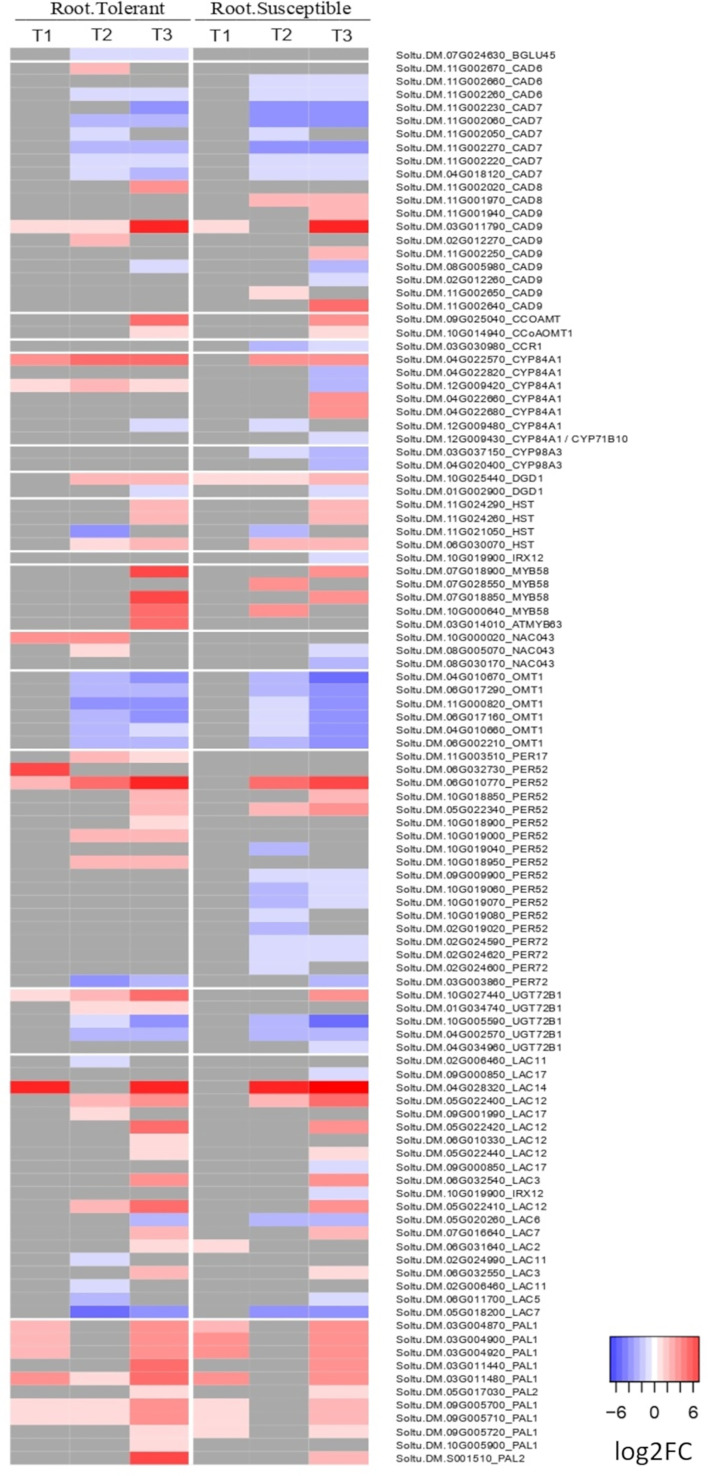
Heatmap of the log2 fold change of DEGs related to lignin biosynthesis in roots. Shown is the log2FC compared to the non-stressed control for all DEGs annotated with lignin biosynthetic process (GO:0009809), lignin catabolic process (GO:0046274) or as laccase genes in the potato genome v6.1. In grey are genes with no significant change in the respective time point (early T1, late T2, and recovery T3), tissue, or variety.

Among the 21 GO terms enriched in the roots of both varieties were those related to salicylic acid, lignin and L-phenylalanine catabolic processes, the cellular response to hypoxia and response to oxidative stress (**Supplementary Figure 3A**). In the lignin catabolic process (GO:0046274) and L-phenylalanine catabolic process (GO:0006559), both varieties upregulated six DEGs encoding phenylalanine ammonia lyases (PALs), which are involved in the first step of production of lignin by converting phenylalanine into cinnamic acid (**Figure 5**). However, there were also unique components of the tolerant response. Among the top 20 most upregulated genes in only the tolerant variety was a laccase gene (Soltu.DM.04G028320) and a peroxidase superfamily protein homologous to Per52 in *A. thaliana* (Soltu.DM.06G032730) (**Supplementary Table 4**), both involved in the polymerization of lignin monomers. The laccase Soltu.DM.04G028320 was also one of the genes with the largest difference in expression between tolerant and susceptible varieties (**Supplementary Table 4**).

While genes involved in the response to oxidative stress were upregulated in the roots of both varieties, including the 6 PAL genes, there were more upregulated genes in the tolerant than in the susceptible variety, including 16 DEGs upregulated only in the tolerant. These included the upregulation of one galactinol synthase (Soltu.DM.02G006360), three peroxidase superfamily genes in addition to *PER52*, and 3 serine-type endopeptidase inhibitors (Soltu.DM.03G003070, Soltu.DM.06G018620, and Soltu.DM.06G018610), 2 of which were among the 20 most upregulated genes in only the tolerant root (**Supplementary Table 7**). There were also 10 enriched GO terms for upregulated genes observed only in the tolerant but not susceptible roots. These included phenylpropanoid and camalexin biosynthetic processes, and with a higher number of DEGs were GO terms related to the response to salt stress and the defense response to fungus (**Supplementary Figure 3A**).

Since the response to ABA was one of the main differences observed between the two varieties, particularly in leaf but also in root, the expression of the potato 9-*cis*-epoxycarotenoid dioxygenase (*NCED*) genes were observed. In roots, Soltu.DM.07G022620 *(AtNCED3*) and Soltu.DM.08G006990 *(AtNCED5*) were upregulated in both varieties across all three time points. At*NCED3* was also upregulated in leaves of both varieties at the later stress time points. Soltu.DM.08G015120, homologous to At*NCED3* and At*NCED1*, was not upregulated in roots or in the susceptible variety at any time point but was upregulated at all three time points in tolerant leaves (**Figure 4**; **Supplementary Table 8**).

Returning to the top 20 most highly upregulated genes in the tolerant variety (but not differentially expressed in the susceptible), we observed cytochrome P450 superfamily proteins in both leaf (Soltu. DM.08G010200, also expressed at a significantly higher level than in the susceptible variety) and root (Soltu.DM.04G027470, Soltu.DM.01G005280) (**Supplementary Tables 4A, B**). At least 8 of the top 20 upregulated genes in the tolerant leaf were involved in processes related directly or indirectly to the ABA response, including transport of organic compounds (sugars, amino acids, lipids), and chaperones controlling protein folding. In the tolerant root, highly upregulated genes included an expansin-like B1 involved in cell wall modification, three genes involved in terpene biosynthesis, and two genes encoding UDP-glycosyl-transferases. The top 20 downregulated genes included three genes encoding NAD(P)-binding Rossmann-fold superfamily proteins, two in leaf (Soltu.DM.10G029820, Soltu.DM.10G025190) and one in root (Soltu.DM.09G001780) (**Supplementary Tables 4A, B**). In leaf, two genes encoding bifunctional inhibitor/lipid-transfer protein/seed storage 2S albumin superfamily proteins were also strongly downregulated in the early response and throughout the duration of stress treatments.

#### 3.2.2 Late responses to drought stress

In the late response to drought, the responses became more similar between the two varieties and tissues. This was reflected in seven shared enriched GO terms for upregulated genes, including the responses to ABA (GO:0009737), hypoxia (GO:0071456), salt stress (GO:0009651), heat (GO:0009408) and water deprivation (GO:0009414), with a large number of DEGs in each term (**Supplementary Figure 3B**). In leaf, most of these GO terms (other than response to hypoxia and salt stress) were already enriched in the tolerant variety at the early time point. A similar pattern was also observed for downregulated genes related to growth, with genes involved in DNA replication (GO:1902975) and cell population proliferation (GO:0008283) reducing their expression in leaf at T1 in the tolerant variety, but not until T2 in the susceptible variety. Similarly, for upregulated genes in roots, there were GO terms enriched in the tolerant variety at the early stage of stress that were not enriched in the susceptible response until T2, including the response to salt stress (GO:0009651) and the ABA activated signaling pathway (GO:0009738) (**Supplementary Figure 3B**).

A total of 19 GO terms were enriched in tolerant but not susceptible leaves at the late time point, of which 4 were also enriched at the early time point (regulation of stomatal movement, regulation of transcription, response to cold and response to chitin). The remaining 15 enriched terms included regulation of the jasmonic acid signaling pathway, salicylic acid biosynthetic process, and more terms related to the defense response against biotic stresses, including wounding (GO:0009611), fungi (GO:0050832), bacteria (GO:0042742) and oomycetes (GO:0002239) (**Supplementary Figure 3B**). Similarly, tolerant roots were enriched for responses to fungus (GO:0050832) and the response to wounding (GO:0009611), though susceptible roots were not. Meanwhile, unique to the late response of the susceptible leaves were upregulated DEGs enriched for processes involved in protein refolding.

**Figure 6:**
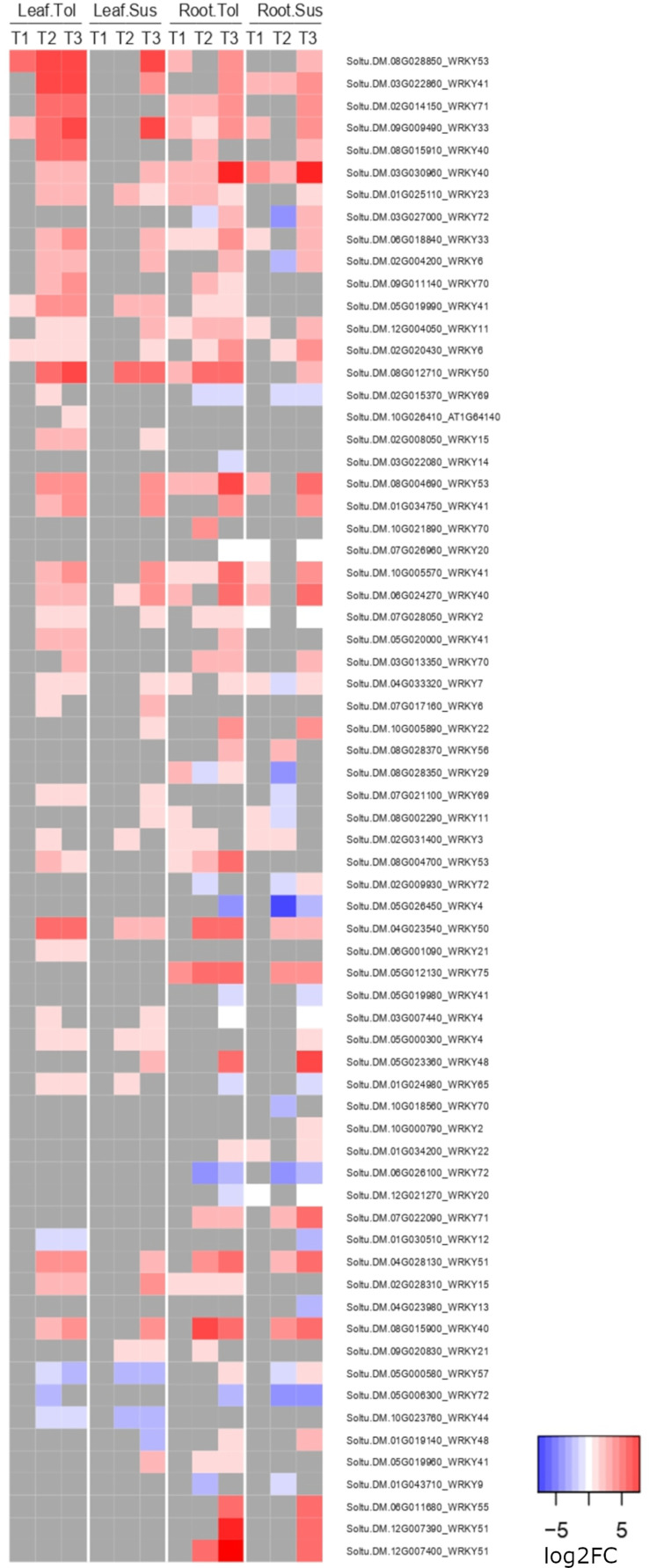
Heatmap of the log2 fold change of WRKY genes. Shown is the log2FC expression of WRKY genes differentially expressed in either tissue in two potato varieties at any time point compared to the non-stressed control.

In both leaves and roots of the tolerant variety, the defense response to fungus process (GO:0050832) was enriched among upregulated genes but was not enriched until the recovery phase in the susceptible variety. These genes included 15 WRKY genes; in leaf, 3 were significantly upregulated in both varieties and 8 uniquely in the tolerant, while in root, 4 were significantly upregulated in both varieties and 8 uniquely in the tolerant, including Soltu.DM.12G007400 *(AtWRKY51)*, which was also one of the top 20 most upregulated genes in the late response of root (**Supplementary Table 9**). Another WRKY gene, Soltu.DM.08G028850 (*WRKY53*), was also in the top 20 DEGs uniquely upregulated in tolerant leaves. Considering all annotated WRKY genes in the potato genome v6.1, the tolerant variety clearly upregulated more WRKY genes in the late response, especially in leaf, four of which also showed an early upregulation in T1 only in the tolerant variety (**Figure 6**). Many of these genes were not upregulated in the susceptible variety until the recovery phase, T3. Within the response to fungus process (GO:0050832), there were also 4 genes whose products interact with calcium, including two calmodulin genes (Soltu.DM.10G026220, Soltu.DM.10G026210) upregulated in all but the susceptible roots during stress (**Supplementary Table 9**).

In the late response of roots, the susceptible variety downregulated more genes than the tolerant variety (**Figures 1D** **;** **3C**), which was reflected in a large number of GO terms uniquely enriched in the susceptible variety (**Supplementary Figure 3B**). These included three terms relating to biosynthesis of lignin (GO:0009809), melatonin (GO:0030187) and aromatic compounds (GO:0019438). Similarly, downregulated genes in the leaves of the susceptible but not the tolerant variety were enriched for lignan (GO:0009807) and cutin (GO:0010143) biosynthetic processes.

Both varieties showed evidence for cell wall remodeling during the late response. In tolerant and susceptible roots, downregulated genes were enriched for the xyloglucan metabolic process (GO:0010411) and cell wall biogenesis (GO:0042546), both involving a large number of xyloglucan endotransglucosylase/hydrolase genes (**Supplementary Table 10**). Only the tolerant variety was enriched for genes involved in cell wall modification (GO:0042545), involving downregulation of 10 genes encoding pectin methylesterase inhibitors, only 4 of which were downregulated in the susceptible. Moreover, among the top 20 genes downregulated only in the tolerant roots were four cell wall related genes, including one xyloglucan endotransglucosylase/hydrolase (Soltu.DM.12G025120), one plant invertase/pectin methylesterase inhibitor superfamily (Soltu.DM.02G001870) and one pectin lyase-like superfamily gene (Soltu.DM.04G014020) (**Supplementary Table 4A**).

### 3.3 Drought and rewatering responsive genes in the tolerant variety

To identify drought-responsive genes in the tolerant variety, we identified genes whose expression significantly increased in the early response, was maintained or continued to be increased in the late response and returned partially or fully towards non-stressed control levels in response to rewatering. Genes that decreased and then recovered their expression were identified similarly. We identified 45 and 176 such genes in leaf and root, respectively. From the 45 genes in leaf, 39 upregulated and 6 genes downregulated their expression in the early response to drought, while in root, these numbers were 131 and 45 genes, respectively (**Supplementary Table 11**).

In leaf, the drought-responsive genes were involved in various processes including cell wall modification, responses against pathogens, starch breakdown, transport and calcium binding, among others **(** **Supplementary Table 11A** **)**. From the 45 genes, 25 showed a stronger response to drought than the susceptible variety (a difference of more than 0.80 log2FC at T1), including 21 upregulated genes and 4 downregulated genes. Notably, Soltu.DM.06G031870, a HSP20-like chaperones superfamily protein and Soltu.DM.08G010200, a cytochrome P450 family 71 subfamily B polypeptide, were particularly highly upregulated at T1, with log2FC values of > 5 and > 4 respectively. Both were in the top 20 upregulated genes in the tolerant variety and both were significantly more upregulated in T1 compared with susceptible variety, which barely changed its expression compared with the control. The susceptible variety only upregulated these genes from T2 onwards, and even then, Soltu.DM.08G010200 was expressed at a significantly lower level than in the tolerant variety, indicating a weaker, delayed response to drought stress.

Among the 25 genes with a quick response to drought and rewatering in the tolerant variety were 12 genes that were not differentially expressed in the susceptible variety in either the early or late response to drought. These included genes related to the response to pathogens (Soltu.DM.12G00530), calcium-binding (Soltu.DM.01G032110) and transcription regulation (Soltu.DM.09G019660). Two of the upregulated genes from control to T1 (Soltu.DM.01G028100, a beta glucosidase and Soltu.DM.01G032110, an EF hand calcium-binding protein family gene) had a higher expression in the tolerant than in the susceptible leaves during T1, and one downregulated gene (Soltu.DM.08G002160, an FAD-dependent oxidoreductase family protein) had a lower expression in the tolerant than the susceptible at T1.

In root, the drought-responsive genes in the tolerant variety included transcription factors, genes that respond to pathogens and genes involved in transport and signaling cascades. Among 176 genes, 42 had more extreme changes compared to the susceptible variety (a difference of more than 0.80 log2FC compared with the susceptible at T1), including 38 upregulated genes and 4 downregulated genes (**Supplementary Table 11B**). Comparing the expression of these 42 genes between the two varieties at each time point, 7 upregulated and 2 downregulated genes had a significant difference in expression between tolerant and susceptible varieties in T1 (**Supplementary Table 11B**). Among the 7 genes were 3 basic chitinases (Soltu.DM.07G005400, Soltu.DM.07G005390, Soltu.DM.02G022960), which were not differentially expressed in the susceptible at any time, and one beta-1,3-glucanase (Soltu.DM.02G033060), which was not differentially expressed in the susceptible variety until the recovery phase.

Among the most highly upregulated genes from control to T1 in the tolerant variety were Soltu.DM.05G002810, encoding an alpha/beta-hydrolases superfamily protein and Soltu.DM.09G019250, encoding an EID1-like protein, whose expression also significantly changed in the susceptible across the 3-time points, but with a lower log2FC. Of the 42 genes shown in **Supplementary Table 11B**, 18 genes were not differentially expressed in the susceptible root from control to T1 or T2, and 15 were not differentially expressed at any time point. These 15 genes that were responding to drought stress only in the tolerant roots included, among the upregulated genes, 2 NAC-domain containing proteins (Soltu.DM.07G014750 and Soltu.DM.10G000020), the three basic chitinases (Soltu.DM.07G005400, Soltu.DM.07G00539, and Soltu.DM.02G022960), and one nitrate transporter (Soltu.DM.06G030890); the downregulated genes included a flavanone 3-hydroxylase (Soltu.DM.02G023850).

## 4 Discussion

The increased frequency and severity of abiotic stress conditions caused by climate change, particularly drought, creates a need to identify key genes and molecular pathways that enable potato plants to adapt to or tolerate the stress (Hijmans, 2003; George et al., 2018). To address this need, the transcriptomic response in leaf and root of two Andean potato varieties with contrasting drought tolerance phenotypes was analysed and key genes and pathways associated with tolerance were identified in the early and late responses to drought.

### 4.1 Faster response of the tolerant variety to drought stress

Both tissues showed a faster change in gene transcription in response to drought in the tolerant compared with the susceptible variety. This was observed in the higher number of DEGs in leaves and in higher fold changes compared with the non-stressed control condition in roots of the tolerant compared with the susceptible variety in the early response. This suggested rapid recognition of the lack of water by the tolerant root and transmission of signals to enable a fast response in leaf. The delayed response of the susceptible leaves was confirmed by observing the different biological processes related to the response to drought stress that began to be enriched in the late response, many of which were already enriched in the tolerant variety since the early time point. One such response was the widespread downregulation of genes involved in DNA replication and cell division, which suggested a generalised shut down/arrest of cell growth. This behaviour is an important mechanism enabling plants to conserve energy under stress, and this reduction under water deficit has been observed to occur independently from changes in photosynthesis (Granier and Tardieu, 1999; Skirycz and Inzé, 2010).

The recovery phase after rewatering produced only a partial recovery of genome-wide gene expression in both varieties. Nevertheless, there were many ‘drought-responsive’ genes in the tolerant variety that increased (or decreased) their expression in response to the stress and whose expression returned towards non-stressed control levels in response to rewatering, and the response of these genes was much reduced in the susceptible variety. In the tolerant root, these genes included several NAC domain-containing proteins whose expression increased under drought but decreased after rewatering. NACs are a large transcription factor family that are involved in diverse biological processes in plants. These include plant development, cell division, senescence, cell wall formation, plant immunity and responses to abiotic stress (Singh et al., 2021). Several NAC proteins respond to hydric stress and regulate genes in the ABA-dependent pathway (Shen et al., 2017; Jiang et al., 2019). In rice, the expression of *OsNAC2* was associated with the increase in ABA by activating the expression of the *NCED3* gene (Jiang et al., 2019). A NAC gene in potato, *StNAC053*, became highly expressed under ABA and drought treatment. When overexpressed in *Arabidopsis* transgenic lines, this gene enabled plants to better tolerate drought compared with the wild type (Wang et al., 2021).

Nitrate transporters also responded quickly to the availability of water in tolerant compared with susceptible roots. Nitrate excretion transporter1 (*NAXT1*), mainly expressed in the cortex of mature roots (Segonzac et al., 2007), is responsible for nitrate $\left(NO_{3}^{-}\right)$ efflux from the root into the external medium and is stimulated by cytoplasmic acidic pH (Aslam et al., 1995; Segonzac et al., 2007). Under drought, it was observed that some nitrate transporters were involved in ABA transport and stomatal closure (Kanno et al., 2012). In both the tolerant leaf and root, several genes encoding 2-oxoglutarate (2OG) and Fe (II)-dependent oxygenase superfamily proteins were upregulated rapidly under drought and downregulated with rewatering but did not respond strongly in the susceptible variety. This superfamily protein is involved in diverse processes in plants and subsequently affects responses to biotic or abiotic stresses; these include DNA repair, histone demethylation, biosynthesis or catabolism of enzymes, such as gibberellin, ethylene, auxin, and salicylic acid, and metabolism of secondary metabolites like flavonoids, and coumarin (Farrow and Facchini, 2014).

### 4.2 Rapid drought-induction of ABA related genes in tolerant leaves and roots

One of the most obvious differences between the tolerant and susceptible varieties was the more widespread and rapid upregulation of ABA-related genes in the early time point, in both tolerant leaves and roots compared with the susceptible variety. Tolerant leaves upregulated a large number of DEGs (33 genes) that were not upregulated in the susceptible variety in the early the response, while tolerant roots upregulated a different set of 19 genes that were also not upregulated in the early response of the susceptible variety. In tolerant leaves, the upregulated genes included 5 PP2C genes, 2 ABA transporters including the ATP-binding cassette family G25 (ABCG25), and 3 ABI five binding proteins (AFPs). Two of these PP2Cs were among the 20 most upregulated DEGs in the tolerant variety. Similarly in roots, the upregulated genes included 5 ABA related transporters. Most of these genes that responded to ABA early in the tolerant variety only began to be upregulated in the susceptible leaves or roots during the late response to drought, showing the delayed response of the susceptible variety, which has also been observed in *Brassica rapa* varieties in response to drought (Guo et al., 2014).

ABA is a phytohormone that regulates different physiological processes under drought, inducing transcriptional reprogramming leading to a variety of outcomes, including stomatal closure and synthesis of osmoprotectants (Sah et al., 2016). ABCG25 is located in the plasma membrane of vascular tissues and functions as an ABA exporter to allow mobilization of this phytohormone towards the guard cells (Ma et al., 2018). AFP proteins, including PP2C proteins, are negative regulators of the ABA response (Lynch et al., 2017). While upregulation of ABCG25 could indicate more effect of the ABA hormone in tolerant leaves, the upregulation of AFP and PP2C genes would suggest that limiting its effects is also important. In the absence of ABA, PP2C interacts with and inactivates SnRK2, but in the presence of ABA, PP2C is inactivated by interaction with PYR/PYL/RCAR receptors, allowing the release of SnRK2. Free SnRK2 phosphorylates and activates itself and other downstream factors, including SLAC1 and SLAH3 transporters involved in ion import into guard cells, and transcription factors, such as AREB/ABF, to mediate stomatal closure and decrease water transpiration (Ali et al., 2020). Therefore, a higher level of PP2C proteins could maintain more inactive SnRK2 to limit or reduce the downstream effects of the ABA signal in the tolerant variety.

It has been reported that higher levels of ABA can be detrimental to plants in various ways, including accelerating senescence and increasing disease susceptibility (Gietler et al., 2020). In addition, plants under drought stress still need an adequate amount of CO_2_ to maintain photosynthesis (Jung et al., 2020), which would also be important for crop yield. Therefore, although the ABA mediated response may be important in response to drought, so too is its effective regulation to make sure that levels are properly modulated so as not to confer a threat. This modulation can occur by regulating the production of ABA or by regulating the response to ABA through the action of PP2C proteins (Jung et al., 2020). Interestingly, the expression of PP2C can in turn be induced by ABA, specifically by the action of the transcription factors AREB/ABF that are activated in the ABA signaling pathway. This may be considered as an important form of negative feedback regulation within the ABA response pathway (Jung et al., 2020). Previous work in potato has also found an increase in PP2C gene expression under drought stress in the leaves of tolerant potato plants (Chen et al., 2020) and in the stolon tissue (Gong et al., 2015). Interestingly, in the potato plants evaluated here, the rate of photosynthesis decreased more rapidly in the susceptible than in the tolerant variety. The tighter regulation of ABA levels through upregulation of PP2C proteins likely allowed the tolerant variety to better maintain its rate of photosynthesis under prolonged stress compared with the susceptible variety.

On the other hand, the early enrichment of genes that respond to ABA in the tolerant variety indicated that there might be more production of this phytohormone in this variety, though ABA levels were not measured in this study. This could be related to the expression of NCED genes. In leaf, an early upregulation of the Soltu.DM.08G015120 gene homologous to *NCED1/NCED3* in *Arabidopsis* was observed in only the tolerant variety, and maintained across all three time points, while the susceptible variety did not upregulate this gene. Since *NCED3* catalyses the rate-limiting step in the ABA biosynthetic pathway, this difference might lead to earlier accumulation of ABA in the tolerant compared with the susceptible leaves. The role of *NCED3* in drought tolerance was previously reported in *Arabidopsis*, where its antisense suppression produced a drought-sensitive phenotype (Iuchi et al., 2001).

Interestingly, the roots of both varieties upregulated *NCED3* and *NCED5* genes throughout drought stress. Previous work also reported that *NCED5* contributes together with *NCED3* to the synthesis of ABA in response to water deficit (Frey et al., 2012). Therefore, in our varieties, functional enrichment of the processes related to ABA was associated with NCED gene expression, which would enable more ABA to be produced at the earlier time point in the roots of both varieties, and potentially more ABA to be produced in tolerant compared with susceptible leaves. Moreover, in tolerant but not susceptible leaves, a beta-glucosidase gene, Soltu.DM.01G028100, was rapidly upregulated in response to drought and recovered its expression after rewatering, but did not significantly respond to stress in the susceptible variety. BGlu enzymes have been associated with the activation of ABA by hydrolyzing ABA glucose ester (ABA-GE) to convert it to free active ABA. In *Oryza sativa*, Os4Glu9, Os4Glu10, Os4BGlu11, Os4BGlu12, and Os4BGlu13 belong to a group of beta glucosidase enzymes that act on ABA-GE (Kongdin et al., 2021). The coding sequence of Soltu.DM.01G028100 was similar to *GLU12*, suggesting that the tolerant variety may not only respond to drought by synthesising ‘new’ ABA, but also by activating pre-existing ABA in the leaf. Overall, these results suggest an earlier, stronger ABA-mediated response in the tolerant compared with the susceptible variety, coupled with more effective feedback regulation.

### 4.3 Conserved lignification of root tissue is enhanced in the tolerant variety

During the early response to drought, there was a conserved upregulation of several genes involved in lignification in both tolerant and susceptible roots, including six phenylalanine ammonia lyases (PALs), which catalyse the first step in lignin biosynthesis. This is consistent with the widely reported role of lignin in enhancing tolerance to drought and other abiotic stresses in many plant species (Moura et al., 2010; Chun et al., 2019; Karlova et al., 2021). Lignin provides rigidity to the cell wall and forms a hydrophobic barrier around the xylem to reduce water loss through leakage, thus facilitating more effective water transport through the plant (Karlova et al., 2021). However, only the tolerant variety strongly upregulated other genes involved in lignin polymerization. These included a laccase homologous to *A. thaliana LAC14* among the top 20 most strongly upregulated genes, and four peroxidase superfamily proteins homologous to *PER52* in *A. thaliana*, one of which was in the top 20 and all of which are involved in the polymerisation of monolignols to produce the final lignin polymer (Chun et al., 2019). These results suggest that the tolerant variety responds to drought stress by more strongly inducing the expression of lignin biosynthetic genes in order to reinforce the plant cell wall and reduce water loss. Similarly, Sprenger et al. (2016) found a constitutively higher expression of genes for lignin biosynthesis in drought tolerant compared with susceptible plants, while Moon et al. (2018) reported upregulation of Laccase 14 in tolerant potato varieties at 6, 12, 24 and 48 hours after drought stress.

### 4.4 ROS-related damage limitation in the tolerant variety

It is well known that ROS accumulation is one of the first responses to stress in plants. Although at high concentrations it can produce severe damage to cellular structures including proteins, lipids, and nucleic acids, at lower concentrations it functions as a stress signal that allows plants to respond to adverse conditions (Halliwell, 2006; Petrov et al., 2015). Although the roots of both varieties responded early to oxidative stress, roots of the tolerant variety upregulated double the number of genes relating to this process in the early response to drought. In particular, only the tolerant variety highly upregulated two genes homologous to *MYB78* in *Arabidopsis* (Soltu.DM.05G023310 and Soltu.DM.12G001820), which may lead to a rapid accumulation of ROS and downstream signaling, as observed in *Brassica napus* where expression of *BnaMYB78* led to ROS accumulation (Chen et al., 2016). On the other hand, in roots of the tolerant variety we also observed more widespread upregulation of genes that may protect against ROS-induced damage, which is consistent with other studies that have shown greater accumulation of sugars, proline, and molecular chaperones in Andean potato varieties under drought stress (Vasquez-Robinet et al., 2008).

Upregulated genes in the tolerant variety included one galactinol synthase and three serine-type endopeptidase inhibitors. These three genes showed a log2FC of more than four in the early response of the tolerant variety, but a negligible change in the susceptible variety. Serine-type endopeptidase inhibitors (SPIs) are enzymes that regulate the action of proteases to avoid excessive protein degradation that could lead to cellular damage (Clemente et al., 2019). The expression of protease inhibitors was highly induced under abiotic stress in *Arabidopsis* and their overexpression conferred resistance against drought, salt, cold, and oxidative stresses (Zhang et al., 2008). In addition, *Arabidopsis* transgenic lines overexpressing an SPI gene had less oxidative damage than the wild type under drought, showing less lipid peroxidation and more antioxidant activities (Malefo et al., 2020). Upregulation of these genes in the tolerant variety could therefore play a key role in avoiding and/or limiting cellular damage. This is consistent with the upregulation of genes involved in protein refolding that was only observed in the susceptible leaf, suggesting this variety had suffered greater protein damage by the late drought stress time point.

Galactinol synthase is involved in the synthesis of raffinose; this enzyme converts UDP-galactose into galactinol, which in turn is converted into raffinose by the raffinose synthase enzyme (Taji et al., 2002). In addition to upregulating a galactinol synthase, only the tolerant variety upregulated a raffinose synthase gene (Soltu.DM.02G033230) in the early drought response. Both genes were also upregulated in the susceptible variety, but not until the later stage of drought stress and to a lesser degree. Moreover, two UDP-glycosyltransferases were among the top 20 most highly upregulated genes in the early response of only the tolerant variety, while related genes in *Arabidopsis* have a known role in cold, salt and drought tolerance (Li et al., 2017). Raffinose family oligosaccharides (RFOs) accumulate during seed development and play an important role in desiccation tolerance of the seed (Taji et al., 2002). In *Arabidopsis* rosettes, over-expression of galactinol synthase results in increased galactinol and raffinose levels and enhanced dehydration tolerance (Taji et al., 2002). The accumulation of galactinol and raffinose in plants can protect against ROS-related damage under stress (ElSayed et al., 2014). The high expression of these antioxidant proteins in only the tolerant variety could be alleviating the oxidative damage produced under drought. Variety-specific accumulation of raffinose and galactinol is supported by other studies that show conserved accumulation in some potato varieties such as Alegria, Milva, Desiree and Saturna (Sprenger et al., 2016), but no accumulation in other Andean varieties, Sullu and SS2613 (Evers et al., 2010).

### 4.5 Biotic stress related response of the tolerant variety is conserved across tissues

In the late response to drought, many genes involved in the response against pathogens, including fungi, bacteria and oomycetes, changed their expression in both tissues. Crosstalk between the responses to abiotic and biotic stresses in plants involves processes that respond to hormones, such as ABA, salicylic acid, or jasmonic acid, as well as ROS generation as a signal of stress (Fujita et al., 2006). Overexpression of transcription factors, such as MYB, NAC, HSF, and WRKY are also involved in this crosstalk (Fujita et al., 2006; Bai et al., 2018). Such crosstalk was clearly observed between these two types of stress, in the early response to drought in roots and increasingly so in the late response, particularly in leaves. Among the genes involved in the crosstalk, the tolerant variety upregulated more WRKY genes in both tissues compared with the susceptible variety. In the potato genome, 129 genes were annotated as a putative WRKY, whose expression responded to different types of stress, such as heat, salt, and drought, and to salicylic acid treatment (Zhang et al., 2017).

Specifically, Soltu.DM.08G028850, annotated as *AtWRKY53* in *Arabidopsis*, was one of the most highly upregulated genes in the tolerant leaves, but was not upregulated in the susceptible leaf until the recovery phase. The same was observed for Soltu.DM.12G007400 *(AtWRKY51)* in the late response of root. Members of the WRKY protein family are involved in the regulation of the ABA pathway, and their overexpression promotes drought tolerance in tomato, tobacco, and rice (Bai et al., 2018). It was reported that expression of *AtWRKY53* was modulated under biotic stress, induced by SA but repressed by JA, and was involved in plant senescence (Zentgraf and Doll, 2019). In contrast to the result observed here, the upregulation of this specific *AtWRKY53* under drought was correlated with reduced drought tolerance (Sun and Yu, 2015), where its overexpression decreased the hydrogen peroxide levels and stomatal closure in *Arabidopsis* lines that did not survive after drought and rewatering. In contrast, the lower reduction in the rate of photosynthesis observed in our tolerant variety could be correlated with reduced stomatal closure.

Other upregulated genes in the tolerant variety relating to biotic stress responses included genes encoding basic chitinases and genes relating to calcium signaling. Under both biotic and abiotic stress, the fluctuation of calcium functions as a signal, activating stress-responsive calcium sensors like calmodulins (CaMs) or calcineurin B-like proteins (CBLs), calcium-dependent protein kinases (CPK) and calcium/calmodulin-dependent protein kinases (CCaMKs) (Ku et al., 2018). The tolerant variety showed stronger upregulation of genes involved in calcium signaling in both tissues, including two calmodulin genes upregulated in all but the susceptible roots (Soltu.DM.10G026220, Soltu.DM.10G026210), and a BCL-2-associated athanogene 6 (*BAG6*) upregulated in all but the susceptible leaves. BAG proteins including Bag6 mediate the response to multiple kinds of stress in *Arabidopsis*, including the response to salt stress (Arif et al., 2021).

Three basic chitinases were only upregulated in the tolerant root during the early and late responses to drought stress and largely recovered after rewatering, while the susceptible variety did not upregulate chitinases at all. Chitinases are enzymes that participate in the first line of the plant defence during PAMP-triggered immunity by degrading chitin, a major component of the fungal cell wall. However, chitinases are not only induced under pathogen attack, but also under salt, cold, and drought stress (Takenaka et al., 2009) and play a role in plant growth and development. In potato, a class I chitinase was observed within a group of genes conferring drought tolerance identified by a yeast functional screening approach (Kappachery et al., 2013). In clover leaves, chitinases and β-1,3 glucanases increase their expression under drought during the early stage of stress, and were significantly correlated with an increase in proline, with a possible role in detoxification of accumulated ammonia under drought (Lee et al., 2008). Here also, the tolerant variety upregulated a beta-1,3-glucanase (Soltu.DM.02G033060), which then recovered its expression after rewatering, while the susceptible variety did not upregulate this gene until the recovery phase. β-1,3 glucanases hydrolyse glycosidic bonds in the glucans of the fungal cell wall, to protect against fungal pathogens (Oide et al., 2013). The upregulation of such genes involved in the conserved pathways between biotic and abiotic stress responses may contribute to the improved response to drought stress in the tolerant variety.

### 4.6 Cell wall remodeling in response to drought stress

Both tolerant and susceptible varieties altered the expression of cell-wall related genes in the early and late responses to drought stress, though the response was stronger and faster in the tolerant variety. In tolerant leaves, more plant invertase/pectin methylesterase inhibitor superfamily proteins (INV/PMEI-SP) were upregulated in the early response compared with the susceptible variety. This family includes pectin methylesterase inhibitors (PMEIs) and invertase inhibitor (INVI) proteins that regulate the PME and INV enzymes, respectively (Coculo and Lionetti, 2022). Since the tolerant variety showed stronger upregulation of PMEIs in leaf, this may translate into more inhibition of the action of PMEs. Under drought, one of the most important mechanisms generated by the plant is the regulation of stomatal aperture/closure. In *Arabidopsis*, the activity of PME upon methylesterified pectin was important for proper regulation of stomatal aperture under heat and drought stress (Wu et al., 2107). *Arabidopsis* mutants not expressing *PME34*, whose activation depended on ABA, showed enhanced stomatal aperture and a lethal phenotype to heat stress (Wu et al., 2017). In pepper, the overexpression of CaPMEI1 increased tolerance to drought in *Arabidopsis* plants (An et al., 2008). Therefore, the regulation of PME by PMEI is an important factor influencing stomatal opening during drought stress.

In contrast to early responses in leaf, in the late response the tolerant roots showed more widespread and stronger downregulation of INV/PMEI-SP genes than the susceptible roots, including one gene in the top 20 most downregulated genes (Soltu.DM.02G001870). PME demethylesterifies oligomers of the pectin backbone, then these blockwise demethylesterified pectins may bind to each other by crosslinking with calcium ions to form a rigid structure called the “egg-box” in which calcium ions interact with molecules of water to keep the cell wall hydrated (Wormit and Usadel, 2018). Therefore, downregulation of PMEIs may facilitate formation of the egg box structure and maintenance of root cell wall hydration. However, different experiments have shown contradictory results in *Arabidopsis* regarding the relationship between root growth and PMEI activity. While in some cases root growth was promoted by overexpression of PMEI (An et al., 2008), in others it was promoted by inhibition of PMEI (Wormit and Usadel, 2018). In rice, the high expression of PMEI provoked a negative effect on plant growth, producing dwarfed plants (Nguyen et al., 2017). In transgenic potato expressing a Petunia PME, whose activity was pronounced in leaf and tubers, more plant growth at the early stage but no difference in growth after 35 days was observed (Pilling et al., 2000). In the case of invertase inhibitors, their expression was favourable against drought in maize (Chen et al., 2019), in contrast to observations in cucumber where overexpression of vacuolar invertase reduced drought tolerance (Chen et al., 2022). Therefore, further investigation is needed to understand the species and tissue-specific effects of PMEIs, PMEs and invertase inhibitors in the abiotic stress response.

Among the most strongly upregulated genes in root during the early response to drought was an expansin like-B1 (*EXLB1*). Expansins are a class of non-enzymatic cell wall proteins that play a role in the regulation of cell growth by disrupting hydrogen bonds, facilitating cell wall loosening and expansion (Marowa et al., 2016). Strong overexpression of an expansin like-B1 gene was also observed in the stolon of potato variety Ningshu under drought stress (Gong et al., 2015). In *Brassica rapa*, *BrEXLB1* was preferentially expressed in root, and under drought stress its expression was highly elevated, contributing to enhanced root growth and drought tolerance (Muthusamy et al., 2020). In maize, Exp1, Exp5 and ExpB8 levels were increased in roots under low water potential, allowing continued root elongation under stress (Wu et al., 2001). Here, while both varieties upregulated expansin-like B1 during the early response to drought stress, the response was much stronger in the tolerant than in the susceptible variety, with log2 fold changes of > 6 and > 2 respectively. Continued strong upregulation of this gene in the tolerant variety throughout the late and recovery responses to drought stress may have contributed to enhanced root growth compared with the susceptible variety. Consistent with this idea, the root hair specific gene Soltu.DM.01G006590 homologous to *Arabidopsis* AT5G22410 and encoding the cell-wall localised peroxidase PER60/RHS18 was strongly downregulated in the late response of only the tolerant variety (log2FC > 5). Its overexpression in *Arabidopsis* mutants reduced root hair length by 16% compared with wild type, thus its downregulation in the tolerant variety may further facilitate cell wall expansion and growth under drought stress.

A common response in the late response of root between both varieties was the downregulation of xyloglucan endotransglucosylase/hydrolase genes (XTHs). However, though the response was common, more XTHs were downregulated in the tolerant variety and to a greater extent than in the susceptible variety, including one gene in the top 20 most downregulated genes (Soltu.DM.12G025120). XTHs have the capacity to cleave and re-ligate the xyloglucan fragments and their increased expression has been correlated with improved drought tolerance (Le Gall et al., 2015). In maize, the expression and the activity of XTHs in the root differed depending on the evaluated region. The expression of XTH in the apical zone was downregulated, while it was upregulated in the subapical zones (Iurlaro et al., 2016). Therefore, it may be important to differentiate the expression of these enzymes in the different root zones to better understand the responses of the tolerant and susceptible varieties.

### 4.7 Conclusions

There are commonalities and differences in the transcriptomic response between potato varieties that differ in their tolerance to drought stress, many of which involve genes related to the plant cell wall. Strikingly, leaves and roots of the tolerant variety show more widespread and stronger upregulation of genes relating to the ABA response in the early stages of stress compared with the susceptible variety, indicating the speed of response may be crucial. Similarly, there is a general early shut down in growth in the tolerant variety that is not seen until the late response in the susceptible variety. The tolerant roots upregulate many more genes involved in the response to oxidative stress than susceptible roots, facilitating maintenance of protein integrity and early accumulation of metabolites including galactinol and raffinose that may enhance desiccation tolerance. In addition, the tolerant roots showed stronger upregulation of genes involved in lignin biosynthesis, which likely strengthens the cell walls and maintains water transport/minimizes water loss under drought stress. In the late response to stress, the tolerant variety upregulates many genes involved in the response to various biotic stresses, including WRKY family proteins, chitinases and glucanases that may modulate hormone signaling and facilitate effective detoxification of cells under drought stress.

## Data availability statement

The original contributions presented in the study are publicly available. This data can be found here: NCBI, PRJNA874012.

## Author contributions

GO conceived and designed the experiments. YT and RL carried out the experiments and RB generated the RNA-seq data. OP and LC led the analysis of the data, with contributions from GO and AP. OP and LC wrote the manuscript with contributions from all authors.

Please see here for full authorship criteria.

## Funding

This work was supported by the Catedra-CONCYTEC in Genómica funcional y AgroNegocios (Grant no. 242-2011-CONCYTEC-OAJ) and FINCyT (grant no. 099-FINCyT-EQUIP-2009)/(076-FINCyT-PIN-2008). OP received a fellowship from FONDECYT–CONCYTEC, Lima, Peru (grant no. 126-2017-FONDECYT).

## Acknowledgments

The authors acknowledge the FONDECYT – CONCYTEC- Peru for the attribution of a PhD grant to OP (grant no. 126-2017-FONDECYT).

## Conflict of interest

RL is employed by Joyn Bio LLC.

The remaining authors declare that the research was conducted in the absence of any commercial or financial relationships that could be construed as a potential conflict of interest.

## Publisher’s note

All claims expressed in this article are solely those of the authors and do not necessarily represent those of their affiliated organizations, or those of the publisher, the editors and the reviewers. Any product that may be evaluated in this article, or claim that may be made by its manufacturer, is not guaranteed or endorsed by the publisher.
