# Supplementary material for: Transcriptome profiling shows a rapid variety-specific response in two Andigenum potato varieties under drought stress
Source: Front Plant Sci. 2022 Sep 27;13:1003907. doi: 10.3389/fpls.2022.1003907 (PMC9551401; doi:10.3389/fpls.2022.1003907)
Supplement: Supplementary file 1 [file DataSheet_1.docx]

Supplementary Material

## Supplementary Figures

1.
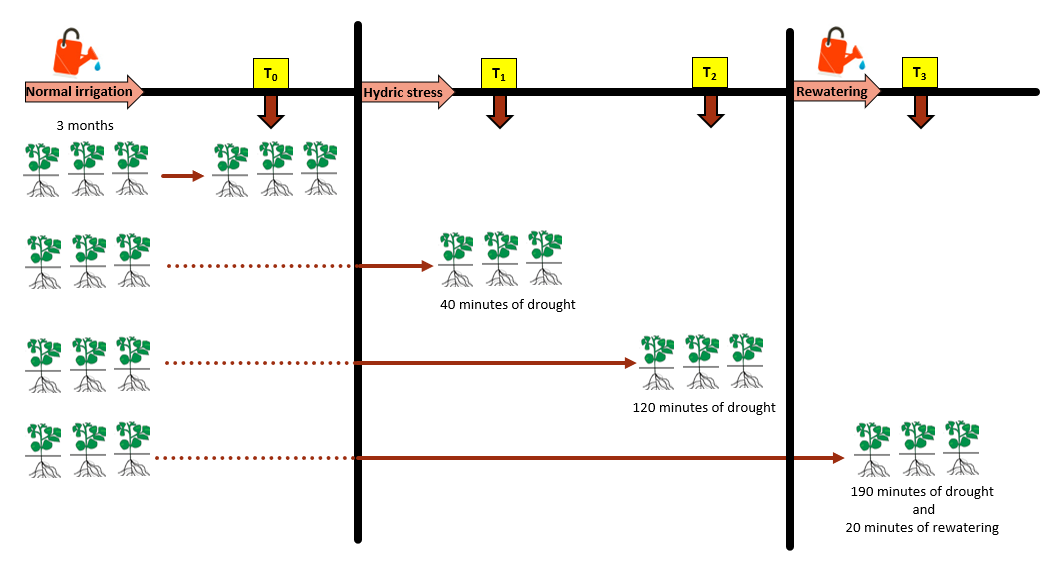
 (B)


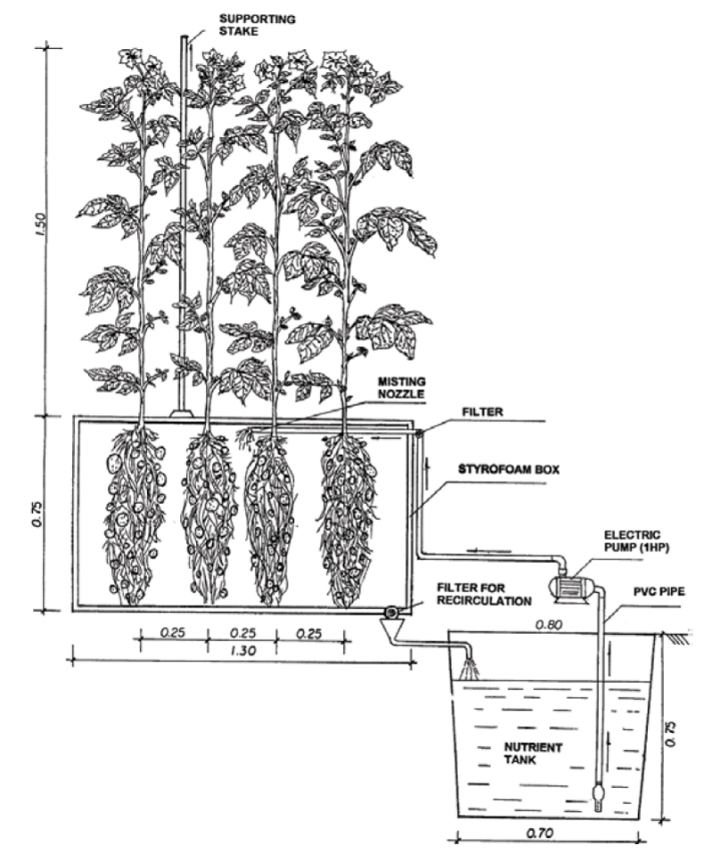


Supplementary Figure 1. Aeroponic system and experimental design. (A) An aeroponic system for quality seed and potato production (reproduced from Figure 1, Otazú, 2010) (B) After 3 months of irrigation, potato plants were exposed to drought stress until observing a decrease of 25% and 60%, and a recovery of 60% of the initial photosynthetic rate in the tolerant variety. These responses were observed at 40 (early response, T1) and 120 (late response, T2) minutes after removing the water supply, and at 20 minutes after rewatering which took place at 190 min (recovery phase, T3). At these time points, leaf and root samples were collected from three plants of each susceptible and tolerant variety. Control plant samples without drought stress were also collected (Control, T0).

**(A)**

| **Tolerant variety** | **Susceptible variety** |
| --- | --- |
| Early response (T_1_)  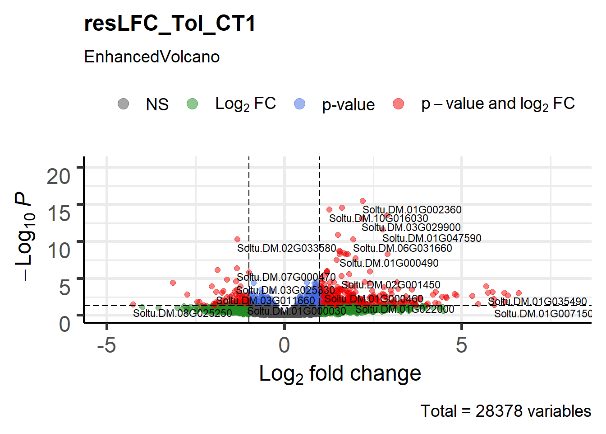  Late response (T_2_)  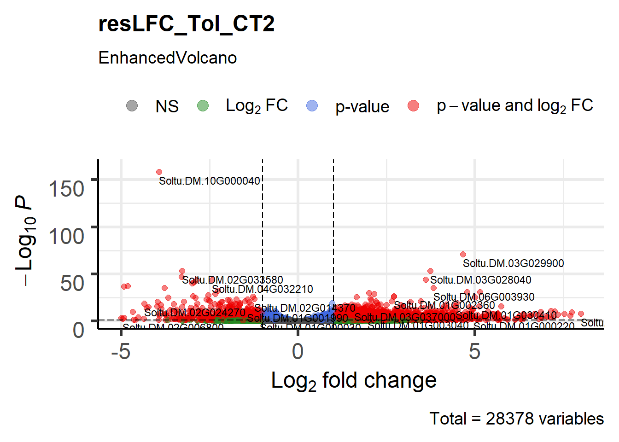  Recovery phase (T_3_)  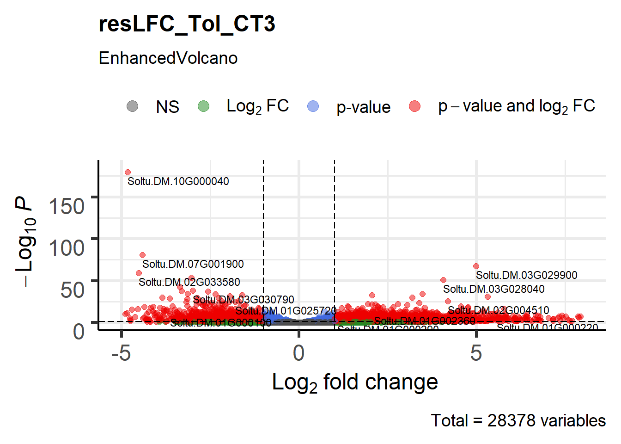 | Early response (T_1_)  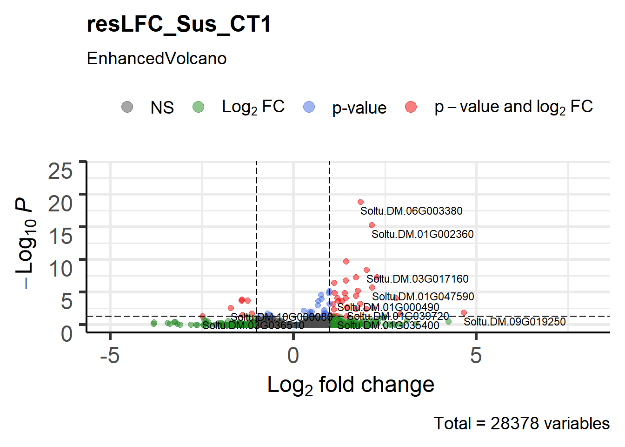  Late response (T_2_)  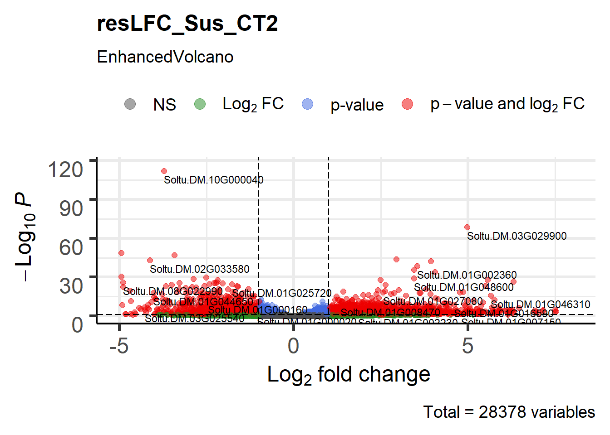  Recovery phase (T_3_)  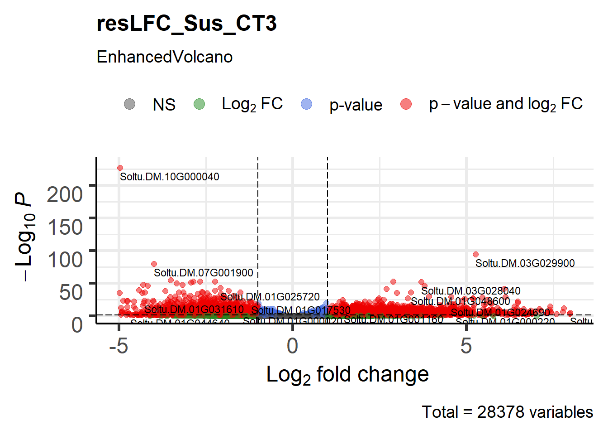 |

**(B)**

| **Tolerant variety** | **Susceptible variety** |
| --- | --- |
| Early response (T_1_)  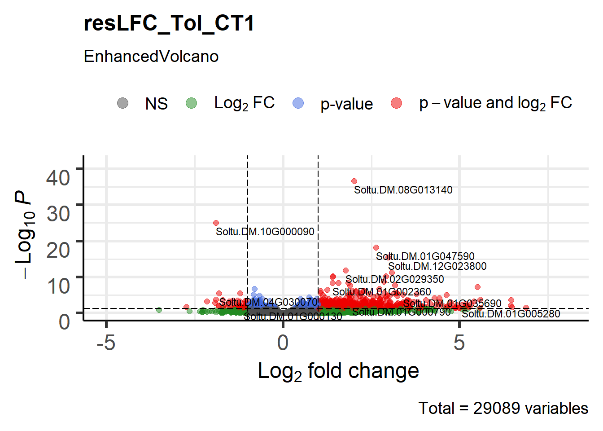  Late response (T_2_)  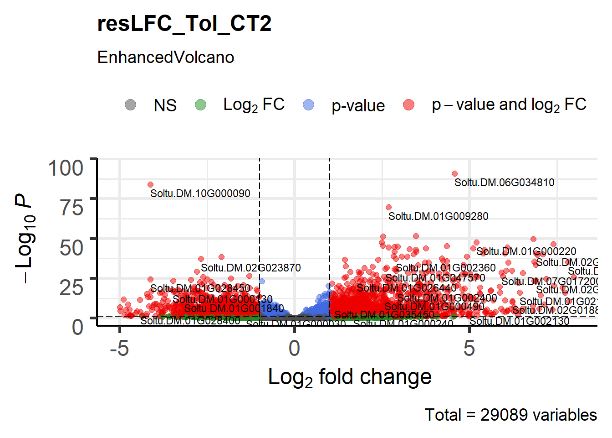  Recovery phase (T_3_)  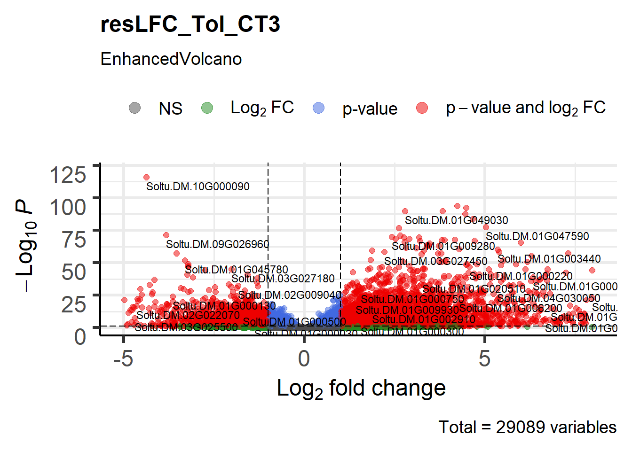 | Early response (T_1_)  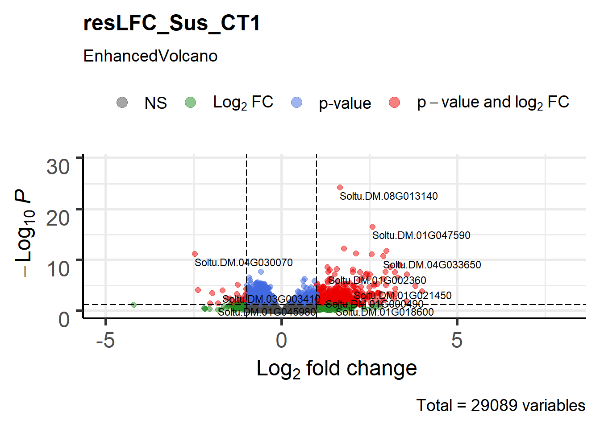  Late response (T_2_)  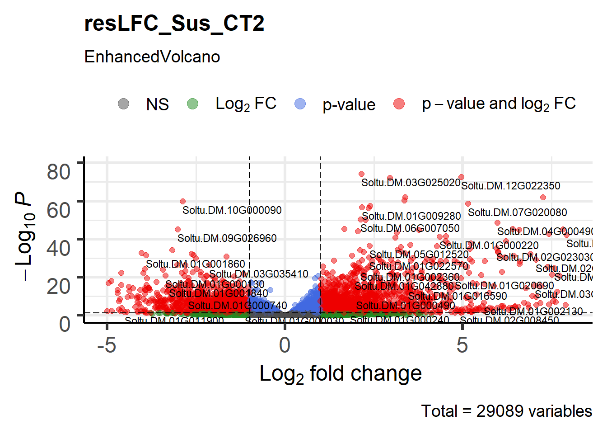  Recovery phase (T_3_)  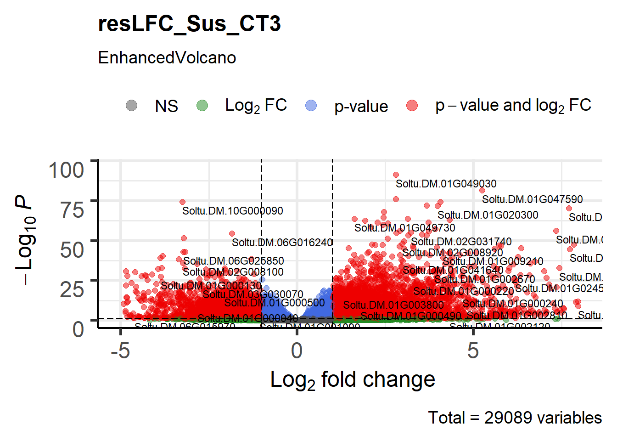 |

**Supplementary Figure 2.** **Progressive drought response in two potato varieties and two tissues.** DEGs are shown in each time point compared with the non-stressed control in leaf (**A**) and root (**B**) for tolerant and susceptible varieties at early (T1) and late (T2) time points and the recovery phase after rewatering (T3). Vertical dashed lines indicate absolute log2FC ≥2. Horizontal dashed lines indicate padj. equal to 5%. Genes passing neither threshold are shown in grey, while non-significant genes passing the FC threshold are shown in green. In blue are those genes with a small but significant fold change and in red are genes passing both thresholds.

**(A) Early, T1**

**
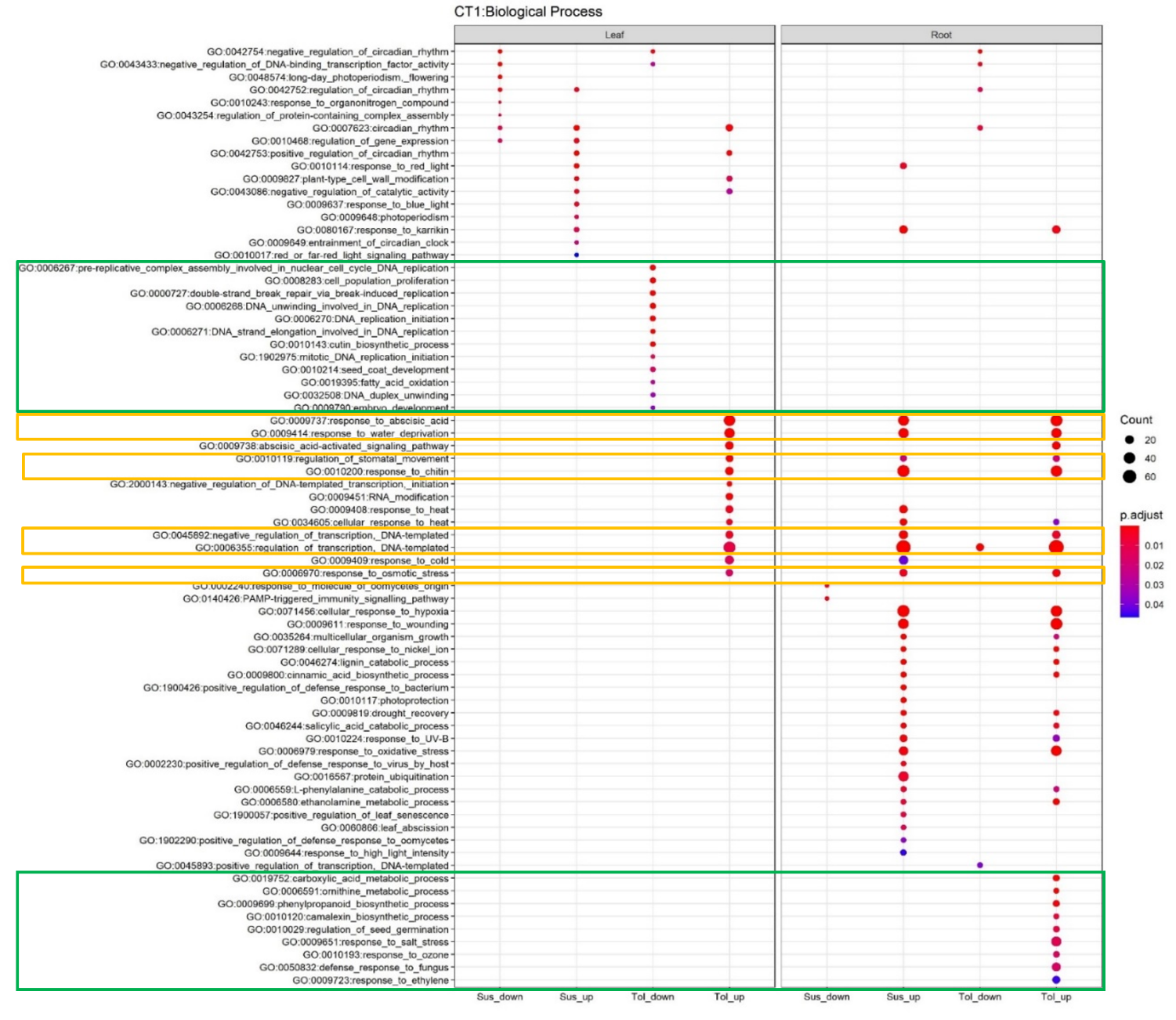
**

**(B) Late, T2**

**
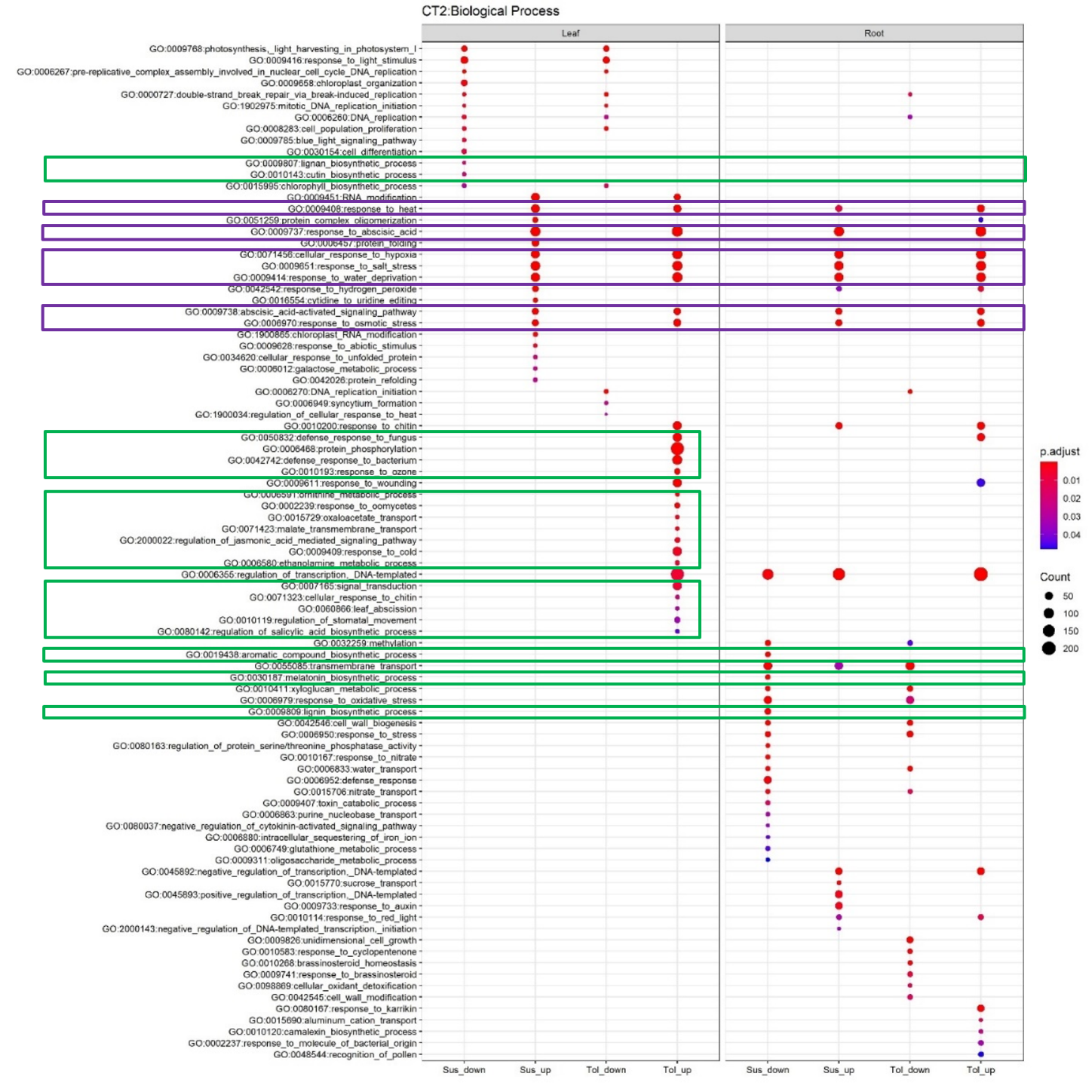
**

**(C) Recovery, T3**


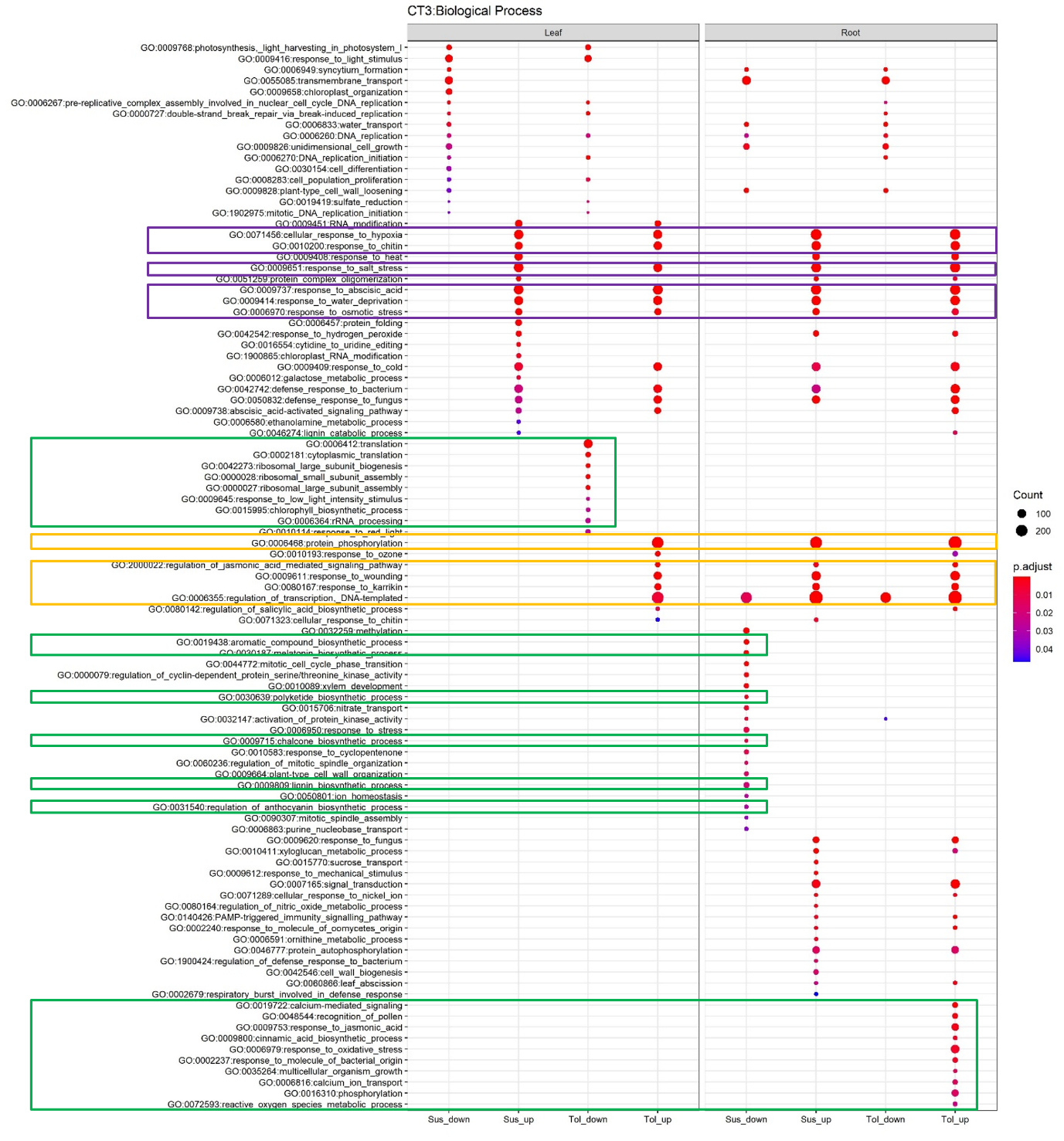


**Supplementary Figure 3.** **Enriched Biological Processes in the Early, Late and Recovery Responses.** Enriched processes are shown for early **(A),** late **(B)** and recovery **(C)** responses to drought. Left and right panes show results in leaf and root tissues for up- and down- regulated DEGs in susceptible (sus) and tolerant (tol) varieties. Significant enrichment is considered at Benjamini-Hochberg adjusted p < 0.05. Green boxes highlight some enriched categories specific to a particular tissue and variety. Orange boxes highlight some enriched GO terms for upregulated genes in all samples apart from susceptible leaf. Purple boxes highlight some terms enriched across both tissues and both varieties.

## Supplementary Tables

| **Neiker Institute Code** | **Variety** | **Species** | **Country** | **Pressure (bar)** | | |
| --- | --- | --- | --- | --- | --- | --- |
|  |  |  |  | **HP1** | **HP2** | **HP2-HP1** |
| NKD-130 | MURO SHOCCO | *S.andigena* | Perú | 2 | 5 | 3 |
| NKD-131 | PUCA HUAYRO | *S. chaucha* | Perú | 2 | 5 | 3 |
| NKD-132 | CHAUCHA | *S. phureja* | Perú | 3 | 5 | 2 |
| NKD-134 | PULU | *S.andigena* | Bolivia | 3 | 11 | 8 |
| NKD-135 | SOCCO HUACCOTO | *S. andigena* | Perú | 2 | 4 | 2 |
| NKD-137 | SIPANCACHI | *S.andigena* | Bolivia | 3 | 6 | 3 |
| NKD-138 | LARAM AJAWIRI | *S. ajanwiri* | Bolivia | 5 | 5 | 0 |
| NKD-139 | JANCKO AJAWIRI | *S. ajanwiri* | Bolivia | 3 | 5 | 2 |
| NKD-140 | MORAR NAYRA MARI | *S. stenotonum* | Perú | 4 | 7 | 3 |
| NKD-141 | UNKNOWN | *S.andigena* | Perú | 3 | 7 | 4 |
| NKD-143 | WILA HUAKA LAJRA | *S.andigena* | Bolivia | 3.5 | 10 | 6.5 |
| NKD-145 | PUCA QUITISH | *S.andigena* | Perú | 3 | 5 | 2 |
| NKD-149 | YANA PPOCCOYA | *S. stenotonum* | Perú | 5 | 6.5 | 1.5 |
| NKD-152 | MORADA TURUNA | *S. stenotonum* | Perú | 4 | 6 | 2 |
| NKD-155 | KASHPADANA AMARILLA | *S. goniocalix* | Perú | 3 | 3 | 0 |
| NKD-156 | HOLANDESA | *S.andigena* | Colombia | 4 | 7 | 3 |
| NKD-157 | UNKNOWN | *S. andigena* | Colombia | 3.5 | 8 | 4.5 |
| NKD-158 | POLUYA | *S. stenotonum* | Perú | 3 | 5 | 2 |
| NKD-159 | CAMUSA | *S. andigena* | Venezuela | 3 | 12 | 9 |
| NKD-160 | CHIMBINA | *S.andigena* | Perú | 3.5 | 5 | 1.5 |
| NKD-161 | NEGRITA | *S.andigena* | Perú | 4 | 4 | 0 |
| NKD-162 | YEMA DE HUEVO | *S. phureja* | Colombia | 2.5 | 4.5 | 2 |
| NKD-163 | COLOR UNCKUNA | *S. chaucha* | Perú | 4.5 | 12 | 7.5 |
| Testigo | MONALISA | *S.tuberosum* | España | 4.5 | 10 | 5.5 |

Supplementary Table 1. Hydric potential (HP) of PapaSalud potato varieties before and after water stress. Shown for each native potato variety is accession information and average hydric potential. HP1 readings were taken from “just watered” plants in an aeroponic system at 20°C with a 16h light regime. HP2 readings were taken 15 days after removal of water supply. The two varieties selected for the current study are shown in red font.

| **Variety** | | | **WILA HUAKA LAJRA** | **NEGRITA** |
| --- | --- | --- | --- | --- |
| Neiker Institute Code | | | NKD-143 | NKD-161 |
| CIP Code | | | CIP 703248 | CIP 703671 |
| Species | | | *S. andigena* | *S. andigena* |
| Country | | | Bolivia | Peru |
| Longitude of collecting site | | | -65.7166 | -78.3439 |
| Latitude of collecting site | | | -17.4333 | -7.0846 |
| Elevation of collecting site (m) | | | 3420 | 3097 |
| Tuber characters | | Pollen Viability | 100 | 50 |
|  |  | Tuber shape | 3 | 2 |
|  |  | Predominant Tuber Skin Color | 5 | 7 |
|  |  | Predominant Sprout Color |  |  |
|  |  | Secondary Sprout Color | 0 | 0 |
|  |  | Distribution of Secondary Sprout Color | 0 | 0 |
|  |  | Predominant Tuber Flesh Color | 2 | 2 |
|  |  | Depth of Tuber Eyes | 1 | 7 |
|  |  | Tuber Skin Type | 5 | 5 |
| Plant characters |  | Growth Habit Type | 4 | 2 |
|  | Leaf characters | Leaf Dissection | 3 | 3 |
|  |  | Lateral Leaflet Number | 2 | 4 |
|  |  | Interjected Leaflets Number among Lateral Leaflets | 2 | 2 |
|  |  | Interjected Leaflets Number on the Petiolules | 0 | 0 |
|  |  | Stem Color | 4 | 6 |
|  |  | Stem Wing |  |  |
|  |  | Degree of Flowering | 5 | 7 |
|  |  | Corolla Shape | 7 | 5 |
|  | Flower Color | Predominant Flower Color | 6 | 8 |
|  |  | Intensity of Predominant Flower Color | 1 | 2 |
|  |  | Secondary Flower Color | 1 | 0 |
|  |  | Distribution of Secondary Flower Color | 9 |  |
|  |  | Anther Pigments | 3 | 2 |
|  |  | Pistil Pigments | 0 | 3 |
|  |  | Calyx Color | 3 | 7 |

**Supplementary Table 2**. **Accession, tuber and plant characteristics for the tolerant variety Negrita and the susceptible variety Wila Huaka Lajra**. Descriptors for each trait based on usage at the International Potato Collection, Peru can be found in: International Board for Plant Genetic Resources (IBPGR) (1977) Descriptors for the cultivated potato. International Board for Plant Genetic Resources Rome, Italy, 47 pp.

| **Library** | **Sample** | **Initial** | **Trimmed** | **%** | **Mapped** | **%** |
| --- | --- | --- | --- | --- | --- | --- |
| **Replicate 1** | | | | | | |
| AA | TLC-1 | 26,304,956 | 26,156,706 | 99.44 | 22,777,230 | 86.59 |
| AB | TRC-1 | 30,840,996 | 30,689,477 | 99.51 | 27,183,400 | 88.14 |
| AC | SLC-1 | 27,976,839 | 27,813,683 | 99.42 | 23,799,346 | 85.07 |
| AD | SRC-1 | 27,993,309 | 27,876,741 | 99.58 | 24,431,616 | 87.28 |
| AE | TL3-1 | 25,424,875 | 25,118,280 | 98.79 | 22,160,674 | 87.16 |
| AF | TR3-1 | 14,717,082 | 14,595,966 | 99.18 | 13,000,115 | 88.33 |
| AG | SL3-1 | 20,067,948 | 19,863,932 | 98.98 | 17,280,268 | 86.11 |
| AH | SR3-1 | 16,975,442 | 16,837,551 | 99.19 | 14,940,540 | 88.01 |
| AI | TL1-1 | 17,079,619 | 16,928,170 | 99.11 | 14,987,216 | 87.75 |
| AJ | TR1-1 | 19,780,525 | 19,622,591 | 99.20 | 17,364,649 | 87.79 |
| AK | SL1-1 | 28,223,430 | 28,023,124 | 99.29 | 23,172,726 | 82.10 |
| AL | SR1-1 | 27,415,791 | 27,233,457 | 99.33 | 23,998,857 | 87.54 |
| AM | TL2-1 | 22,260,826 | 22,103,981 | 99.30 | 19,354,610 | 86.94 |
| AN | TR2-1 | 23,292,392 | 23,065,199 | 99.02 | 20,639,123 | 88.61 |
| AO | SL2-1 | 23,408,804 | 23,091,712 | 98.65 | 19,844,045 | 84.77 |
| AP | SR2-1 | 24,620,110 | 24,397,771 | 99.10 | 21,557,819 | 87.56 |
| **Replicate 2** | | | | | | |
| BA | TLC-2 | 46,550,628 | 46,228,457 | 99.31 | 40,877,664 | 87.81 |
| BB | TRC-2 | 30,706,596 | 30,215,910 | 98.40 | 27,145,973 | 88.40 |
| BC | SLC-2 | 26,077,819 | 25,330,779 | 97.14 | 22,169,365 | 85.01 |
| BD | SRC-2 | 35,020,751 | 34,514,605 | 98.55 | 30,536,729 | 87.20 |
| BE | TL3-2 | 36,111,457 | 35,848,611 | 99.27 | 32,055,002 | 88.77 |
| BF | TR3-2 | 39,146,541 | 38,586,768 | 98.57 | 34,664,659 | 88.55 |
| BG | SL3-2 | 37,473,883 | 37,111,310 | 99.03 | 32,793,862 | 87.51 |
| BH | SR3-2 | 46,512,109 | 45,954,048 | 98.80 | 40,720,535 | 87.55 |
| BI | TL1-2 | 50,760,622 | 49,934,704 | 98.37 | 44,185,061 | 87.05 |
| BJ | TR1-2 | 34,893,186 | 34,409,538 | 98.61 | 30,272,810 | 86.76 |
| BK | SL1-2 | 22,931,546 | 22,784,776 | 99.36 | 19,934,398 | 86.93 |
| BL | SR1-2 | 26,421,383 | 26,178,495 | 99.08 | 22,932,702 | 86.80 |
| BM | TL2-2 | 43,378,704 | 43,026,919 | 99.19 | 38,436,768 | 88.61 |
| BN | TR2-2 | 65,443,855 | 64,864,862 | 99.12 | 57,840,610 | 88.38 |
| BO | SL2-2 | 37,499,070 | 36,960,678 | 98.56 | 32,322,585 | 86.20 |
| BP | SR2-2 | 33,478,561 | 33,054,710 | 98.73 | 29,114,541 | 86.96 |
| **Replicate 3** | | | | | | |
| CA | TLC-3 | 23,835,480 | 23,648,008 | 99.21 | 20,944,308 | 87.87 |
| CB | TRC-3 | 32,458,154 | 32,189,703 | 99.17 | 28,666,079 | 88.32 |
| CC | SLC-3 | 44,146,851 | 42,459,608 | 96.18 | 36,741,837 | 83.23 |
| CD | SRC-3 | 43,620,934 | 43,151,864 | 98.92 | 38,432,898 | 88.11 |
| CE | TL3-3 | 51,048,669 | 50,507,050 | 98.94 | 45,143,511 | 88.43 |
| CF | TR3-3 | 49,055,736 | 48,663,688 | 99.20 | 43,932,011 | 89.56 |
| CG | SL3-3 | 40,740,771 | 40,235,487 | 98.76 | 33,879,880 | 83.16 |
| CH | SR3-3 | 59,552,325 | 58,455,003 | 98.16 | 51,906,375 | 87.16 |
| CI | TL1-3 | 46,415,103 | 44,879,577 | 96.69 | 39,301,492 | 84.67 |
| CJ | TR1-3 | 33,341,378 | 32,625,336 | 97.85 | 29,075,254 | 87.20 |
| CK | SL1-3 | 55,247,111 | 54,841,731 | 99.27 | 48,340,865 | 87.50 |
| CL | SR1-3 | 54,778,319 | 54,342,632 | 99.20 | 47,851,250 | 87.35 |
| CM | TL2-3 | 31,377,679 | 30,704,322 | 97.85 | 27,518,051 | 87.70 |
| CN | TR2-3 | 40,295,595 | 39,361,665 | 97.68 | 35,046,577 | 86.97 |
| CO | SL2-3 | 56,400,098 | 55,659,431 | 98.69 | 48,984,525 | 86.85 |
| CP | SR2-3 | 37,945,230 | 37,681,160 | 99.30 | 33,724,034 | 88.88 |

Supplementary Table 3. Sample QC and read mapping statistics. The total number of reads is shown for each of 48 samples, together with the number and percentage of trimmed or mapped reads. Percentages are given based on the total number of reads. Samples are coded as “VarietyTissueTreatment-Replicate”. For variety, S denotes susceptible and T denotes tolerant. For tissue, L is leaf and R is root. Treatments are denoted control (C), early time point (1), late time point (2) and recovery time point (3). Biological replicates are numbered 1-3.

1. **Root, T1**

| **Potato genome v6.1  Gene ID** | **Potato genome v6.1  Gene Annotation** | **Root. Tolerant** | | | | | | **Root. Susceptible** | | | | | |
| --- | --- | --- | --- | --- | --- | --- | --- | --- | --- | --- | --- | --- | --- |
|  |  | **T1** | | **T2** | | **T3** | | **T1** | | **T2** | | **T3** | |
|  |  | **LFC** | **padj** | **LFC** | **padj** | **LFC** | **padj** | **LFC** | **padj** | **LFC** | **padj** | **LFC** | **padj** |
| **Upregulated genes** | | | | | | | | | | | | | |
| **Cell wall modification** | | | | | | | | | | | | | |
| Soltu.DM.08G001190 | expansin-like B1 | 6.87 | 2.80E-02 | 4.09 | 1.58E-01 | 7.69 | 7.63E-04 | 2.34 | NA | 3.18 | 1.74E-01 | 4.51 | 2.23E-02 |
| **Lignin synthesis** |  |  |  |  |  |  |  |  |  |  |  |  |  |
| Soltu.DM.04G028320***** | Laccase | 5.14 | 4.34E-02 | 1.72 | 5.12E-01 | 5.39 | 3.17E-03 | 2.51 | NA | 5.24 | 2.02E-02 | 6.89 | 6.24E-04 |
| Soltu.DM.06G032730 | Peroxidase superfamily protein | 4.93 | 5.92E-03 | 2.45 | 1.78E-01 | 1.88 | 2.44E-01 | 0.83 | 7.25E-01 | 1.19 | 5.46E-01 | 2.10 | 1.70E-01 |
| **Terpene biosynthesis** |  |  |  |  |  |  |  |  |  |  |  |  |  |
| Soltu.DM.01G040960 | terpene synthase | 5.17 | 1.01E-02 | 2.92 | 1.37E-01 | 4.81 | 2.03E-03 | 0.53 | NA | 0.79 | 7.16E-01 | -0.68 | 7.11E-01 |
| Soltu.DM.01G040950 | terpene synthase | 4.89 | 2.32E-02 | 3.52 | 7.27E-02 | 4.91 | 2.28E-03 | 1.51 | NA | 1.95 | 3.26E-01 | -1.27 | 5.31E-01 |
| Soltu.DM.01G040930 | terpene synthase | 4.77 | 3.72E-03 | 3.51 | 2.51E-02 | 4.57 | 4.50E-04 | 0.55 | 7.96E-01 | -1.57 | 3.17E-01 | -2.46 | 5.51E-02 |
| **UDP-glycosyltransferases** |  |  |  |  |  |  |  |  |  |  |  |  |  |
| Soltu.DM.09G031020***** | UDP-Glycosyltransferase superfamily protein | 5.58 | 3.27E-02 | 5.21 | 1.61E-02 | 6.20 | 1.04E-03 | -2.43 | NA | 2.67 | 1.97E-01 | 2.10 | 2.77E-01 |
| Soltu.DM.04G011110 | UDP-glycosyltransferase 73B4 | 4.98 | 2.76E-02 | 4.03 | 4.08E-02 | 2.85 | 1.58E-01 | -0.64 | NA | -3.60 | 5.78E-04 | 0.21 | 8.72E-01 |
| **Others** |  |  |  |  |  |  |  |  |  |  |  |  |  |
| Soltu.DM.03G003070 | serine-type endopeptidase inhibitors | 6.45 | 2.50E-04 | 0.22 | 9.39E-01 | 1.86 | 2.93E-01 | 0.49 | NA | -0.15 | 9.55E-01 | -0.31 | 8.70E-01 |
| Soltu.DM.06G018620***** | serine-type endopeptidase inhibitors | 5.33 | 3.28E-03 | -1.40 | 5.67E-01 | 0.63 | 7.59E-01 | 0.26 | NA | -1.87 | 3.64E-01 | 0.40 | 8.37E-01 |
| Soltu.DM.12G001820 | myb domain protein | 6.44 | 7.32E-03 | 4.03 | 7.46E-02 | 9.26 | 2.20E-07 | 2.67 | 1.52E-01 | 1.39 | 4.70E-01 | 6.03 | 5.71E-06 |
| Soltu.DM.05G023310 | myb domain protein | 5.21 | 1.26E-02 | 5.68 | 8.26E-04 | 6.37 | 3.25E-05 | 1.23 | NA | 3.10 | 3.82E-03 | 4.27 | 9.49E-06 |
| Soltu.DM.02G006200***** | Dehydrin domain containing protein | 5.27 | 1.99E-02 | 8.54 | 8.88E-07 | 10.35 | 5.99E-11 | 0.64 | 8.50E-01 | 9.30 | 5.81E-10 | 9.66 | 3.98E-11 |
| Soltu.DM.04G027470 | Cytochrome P450 superfamily protein | 5.40 | 4.92E-04 | 6.08 | 1.62E-05 | 7.59 | 4.67E-10 | 1.11 | 5.40E-01 | 7.06 | 1.80E-10 | 7.25 | 2.10E-11 |
| Soltu.DM.01G005280 | cytochrome P450, family 71, subfamily B, polypeptide | 5.06 | 5.71E-03 | 5.96 | 4.37E-05 | 6.02 | 1.22E-05 | 0.94 | NA | 1.53 | 1.27E-03 | 1.04 | 2.97E-02 |
| Soltu.DM.02G027440 | phytosulfokine 6 precursor | 5.17 | 2.25E-03 | 5.08 | 8.85E-04 | 4.62 | 7.35E-04 | 0.78 | 6.72E-01 | 1.47 | 3.10E-01 | 3.46 | 1.96E-03 |
| Soltu.DM.01G047920 | Cupredoxin superfamily protein | 5.00 | 3.08E-02 | 2.92 | 1.79E-01 | 3.99 | 2.41E-02 | 1.75 | NA | 1.02 | 6.61E-01 | 3.81 | 1.63E-02 |
| Soltu.DM.01G047870 | Cupredoxin superfamily protein | 4.87 | 3.28E-02 | 2.64 | 2.25E-01 | 2.64 | 1.53E-01 | 0.94 | 7.34E-01 | 0.68 | 7.99E-01 | 3.04 | 8.02E-02 |
| Soltu.DM.01G039820 | Protein kinase family protein | 4.88 | 3.24E-02 | 10.42 | 9.32E-11 | 10.03 | 3.61E-11 | -0.07 | 9.82E-01 | 5.23 | 1.61E-06 | 4.73 | 9.00E-06 |
| Soltu.DM.03G015580 | hypothetical protein | 6.49 | 1.92E-02 | 1.61 | 6.54E-01 | 7.43 | 2.89E-04 | 2.51 | NA | -0.75 | 8.09E-01 | 5.11 | 2.82E-03 |
| **Downregulated genes** |  |  |  |  |  |  |  |  |  |  |  |  |  |
| **Circadian Clock** |  |  |  |  |  |  |  |  |  |  |  |  |  |
| Soltu.DM.10G000080***** | circadian clock associated / | -1.92 | 1.24E-03 | -4.90 | 1.40E-12 | -3.96 | 3.85E-14 | -0.57 | 4.18E-01 | -2.76 | 1.90E-08 | -2.71 | 1.59E-08 |
|  | Homeodomain-like superfamily protein |  |  |  |  |  |  |  |  |  |  |  |  |
| Soltu.DM.10G000090***** | Homeodomain-like superfamily protein | -1.91 | 9.55E-26 | -4.13 | 8.72E-85 | -4.38 | 9.97E-117 | -0.72 | 7.17E-04 | -2.88 | 1.63E-60 | -3.25 | 3.57E-75 |
| Soltu.DM.10G023770 | Homeodomain-like superfamily protein | -1.66 | 3.69E-03 | -1.43 | 8.04E-03 | -2.12 | 2.43E-06 | -1.12 | 5.05E-02 | -0.82 | 1.24E-01 | -1.50 | 8.27E-04 |
| **Cell wall modification** |  |  |  |  |  |  |  |  |  |  |  |  |  |
| Soltu.DM.05G008140 | xyloglucan:xyloglucosyl transferase | -1.50 | 2.30E-02 | -1.46 | 1.27E-02 | 1.49 | 1.41E-03 | -0.60 | 3.89E-01 | -1.05 | 5.57E-02 | 1.77 | 1.10E-04 |
| **Others** |  |  |  |  |  |  |  |  |  |  |  |  |  |
| Soltu.DM.01G049280***** | conserved hypothetical protein | -2.75 | 1.60E-02 | -3.37 | 7.92E-04 | -0.76 | 4.67E-01 | -0.84 | 5.18E-01 | -1.46 | 1.58E-01 | -1.98 | 2.44E-02 |
| Soltu.DM.11G003320 | basic helix-loop-helix (bHLH) DNA-binding superfamily protein | -1.91 | 1.46E-04 | -2.97 | 9.45E-10 | -2.65 | 1.89E-10 | -0.08 | 9.31E-01 | -0.77 | 1.51E-01 | -1.89 | 4.06E-05 |
| Soltu.DM.02G028760 | basic helix-loop-helix (bHLH) DNA-binding superfamily protein | -1.57 | 2.51E-02 | -0.49 | 4.96E-01 | 0.68 | 2.32E-01 | -0.67 | 3.60E-01 | -0.05 | 9.62E-01 | -0.60 | 2.97E-01 |
| Soltu.DM.02G023850 | flavanone 3-hydroxylase | -2.15 | 5.06E-04 | -2.85 | 1.23E-05 | -0.72 | 1.58E-01 | -0.92 | NA | -0.23 | 7.40E-01 | 0.68 | 1.49E-01 |
| Soltu.DM.06G008600 | nodulin MtN21 /EamA-like transporter family protein | -1.83 | 1.37E-02 | -3.95 | 1.08E-09 | -3.67 | 4.08E-11 | -0.67 | 4.18E-01 | -3.93 | 2.94E-12 | -4.09 | 2.31E-13 |
| Soltu.DM.03G020100 | Nodulin MtN3 family protein | -1.83 | 7.50E-03 | 2.01 | 4.11E-04 | 2.37 | 7.56E-07 | -0.65 | 3.82E-01 | 2.93 | 1.13E-09 | 3.30 | 1.29E-12 |
|  |  |  |  |  |  |  |  |  |  |  |  |  |  |
| Soltu.DM.03G025460***** | cytochrome P450, family 71, subfamily A, polypeptide | -1.72 | 1.96E-04 | -4.23 | 5.09E-16 | -4.13 | 6.94E-21 | 0.07 | 9.25E-01 | -3.13 | 1.69E-13 | -3.74 | 4.45E-17 |
| Soltu.DM.09G017200 | cytochrome P450, family 82, subfamily G, polypeptide | -1.69 | 1.46E-02 | -1.58 | 1.22E-02 | -0.05 | 9.50E-01 | -0.66 | NA | -1.31 | 7.48E-02 | -1.55 | 2.10E-02 |
| Soltu.DM.11G024560 | alpha/beta-Hydrolases superfamily protein | -1.53 | 2.87E-03 | -2.10 | 4.24E-05 | -2.15 | 6.07E-07 | -0.46 | NA | -1.53 | 2.60E-03 | -2.49 | 3.77E-06 |
| Soltu.DM.02G030190 | Adenosylmethionine decarboxylase family protein | -1.52 | 5.19E-04 | -1.52 | 2.69E-04 | 0.78 | 4.16E-02 | -0.99 | 2.86E-02 | -0.61 | 1.65E-01 | 1.92 | 3.20E-08 |
| Soltu.DM.03G019030***** | Homeodomain-like superfamily protein | -1.42 | 3.02E-04 | -2.92 | 2.51E-16 | -4.15 | 1.05E-37 | -0.10 | 8.67E-01 | -2.67 | 1.72E-17 | -3.84 | 1.18E-33 |
| Soltu.DM.09G001780 | NAD(P)-binding Rossmann-fold superfamily protein | -1.41 | 3.57E-02 | -2.88 | 1.07E-05 | -1.98 | 8.46E-05 | -0.85 | NA | -1.33 | 3.69E-02 | -1.96 | 1.33E-03 |
| Soltu.DM.02G030180 | conserved peptide upstream open reading frame | -1.37 | 5.17E-03 | -1.47 | 1.02E-03 | 1.24 | 1.10E-03 | -0.58 | 2.86E-01 | -0.16 | 7.96E-01 | 2.45 | 5.67E-12 |
| Soltu.DM.04G028940 | STAS domain / Sulfate transporter family | -1.33 | 1.37E-02 | -0.42 | 4.34E-01 | 0.25 | 6.10E-01 | -0.51 | NA | 0.49 | 3.62E-01 | 0.29 | 5.62E-01 |
| Soltu.DM.09G029770***** | terpene synthase | -1.31 | 4.78E-02 | -2.56 | 1.04E-05 | -2.50 | 3.32E-07 | -0.01 | 9.92E-01 | -1.90 | 1.91E-05 | -1.32 | 2.61E-03 |
| Soltu.DM.04G005260 | dentin sialophosphoprotein-related | -1.29 | 2.97E-04 | -2.52 | 3.25E-15 | -3.15 | 3.73E-29 | -0.36 | 4.12E-01 | -1.51 | 2.98E-07 | -1.87 | 4.11E-11 |

1. **Leaf, T1**

| **Potato genome v6.1  Gene ID** | **Potato genome v6.1  Gene Annotation** | **Leaf. Tolerant** | | | | | | **Leaf. Susceptible** | | | | | |  |
| --- | --- | --- | --- | --- | --- | --- | --- | --- | --- | --- | --- | --- | --- | --- |
|  |  | **T1** | | **T2** | | **T3** | | **T1** | | **T2** | | **T3** | |  |
|  |  | **LFC** | **padj** | **LFC** | **padj** | **LFC** | **padj** | **LFC** | **padj** | **LFC** | **padj** | **LFC** | **padj** |  |
| **Upregulated genes** |  |  |  |  |  |  |  |  |  |  |  |  |  |  |
| **Response to ABA** |  |  |  |  |  |  |  |  |  |  |  |  |  |  |
| Soltu.DM.06G013730 | protein phosphatase 2CA | 6.20 | 8.13E-04 | 4.36 | 7.23E-03 | 5.45 | 1.53E-03 | 2.78 | 9.53E-01 | 3.65 | 4.56E-02 | 2.64 | 8.99E-02 |  |
| Soltu.DM.03G012480 | protein phosphatase 2CA | 4.56 | 3.01E-03 | 6.39 | 1.36E-07 | 7.33 | 4.49E-08 | 2.10 | 1.00E+00 | 6.16 | 8.93E-06 | 6.57 | 2.28E-08 |  |
| **Transport** |  |  |  |  |  |  |  |  |  |  |  |  |  |  |
| Soltu.DM.06G010580 | Transmembrane amino acid transporter family protein | 6.60 | 9.32E-04 | 9.51 | 2.52E-09 | 10.06 | 3.87E-09 | -0.03 | 1.00E+00 | 4.00 | 1.23E-02 | 5.61 | 1.11E-05 |  |
| Soltu.DM.10G018680 | lipid transfer protein | 5.29 | 2.22E-03 | 5.62 | 7.31E-05 | 6.05 | 6.10E-05 | 0.16 | 1.00E+00 | 1.95 | 2.30E-01 | 2.76 | 2.47E-02 |  |
| Soltu.DM.12G025220 | sugar transporter | 4.57 | 4.62E-03 | 3.61 | 8.28E-03 | 2.46 | 1.16E-01 | 0.50 | 1.00E+00 | 0.89 | 6.04E-01 | 0.72 | 5.86E-01 |  |
| Soltu.DM.10G018810 | lipid transfer protein | 4.26 | 6.55E-04 | 4.41 | 2.43E-05 | 4.38 | 1.13E-04 | -0.12 | 1.00E+00 | 2.28 | 5.03E-02 | 2.93 | 1.57E-03 |  |
| **Heat shock proteins** |  |  |  |  |  |  |  |  |  |  |  |  |  |  |
| Soltu.DM.06G013460 | heat shock protein 90.1 | 6.22 | 1.39E-02 | 4.46 | 3.38E-02 | 1.68 | 5.40E-01 | 1.92 | 1.00E+00 | 7.50 | 6.70E-04 | 5.75 | 2.41E-03 |  |
| Soltu.DM.06G031870 | HSP20-like chaperones superfamily protein | 5.45 | 2.74E-02 | 5.90 | 2.10E-03 | 4.68 | 3.17E-02 | -0.06 | 1.00E+00 | 6.15 | 4.80E-03 | 5.51 | 2.59E-03 |  |
| **Stress responsive genes** |  |  |  |  |  |  |  |  |  |  |  |  |  |  |
| Soltu.DM.07G011880 | heat shock transcription factor A2 | 4.44 | 2.79E-03 | 4.50 | 2.27E-04 | 5.71 | 1.43E-05 | 2.63 | 6.76E-01 | 4.59 | 8.88E-04 | 4.95 | 1.77E-05 |  |
| Soltu.DM.10G003790 | late embryogenesis abundant domain-containing protein / LEA domain-containing protein | 4.33 | 2.37E-02 | 5.57 | 1.27E-04 | 7.28 | 3.02E-06 | 1.80 | 1.00E+00 | 3.77 | 2.81E-02 | 4.13 | 3.08E-03 |  |
| Soltu.DM.03G004180 | late embryogenesis abundant domain-containing protein / LEA domain-containing protein | 4.03 | 2.39E-02 | 4.63 | 7.29E-04 | 4.94 | 9.04E-04 | 1.72 | 1.00E+00 | 3.90 | 1.36E-02 | 3.08 | 2.23E-02 |  |
| Soltu.DM.02G024670 | Dehydrin domain containing protein | 5.68 | 1.11E-04 | 7.73 | 2.03E-10 | 8.61 | 1.84E-10 | 0.10 | 1.00E+00 | 3.79 | 1.24E-02 | 4.83 | 7.73E-05 |  |
| **Others** |  |  |  |  |  |  |  |  |  |  |  |  |  |  |
| Soltu.DM.06G026880 | Octicosapeptide/Phox/Bem1p family protein | 6.29 | 1.86E-03 | 4.72 | 6.84E-03 | 5.61 | 2.64E-03 | 4.24 | 3.05E-01 | 2.92 | 1.68E-01 | 2.91 | 8.69E-02 |  |
| Soltu.DM.01G007150 | CAP160 protein | 5.91 | 3.61E-02 | 8.95 | 1.22E-05 | 10.45 | 1.01E-06 | 0.08 | 1.00E+00 | 4.80 | 1.43E-02 | 6.30 | 7.22E-05 |  |
| Soltu.DM.01G027460 | Expressed protein | 5.90 | 6.02E-03 | 7.14 | 2.74E-05 | 8.48 | 4.91E-06 | 2.98 | 1.00E+00 | 6.95 | 3.06E-04 | 7.32 | 5.88E-06 |  |
| Soltu.DM.03G024470 | conserved hypothetical protein | 5.86 | 6.07E-04 | 6.56 | 3.70E-06 | 8.03 | 2.09E-07 | 2.51 | 1.00E+00 | 5.30 | 1.24E-03 | 5.56 | 4.87E-05 |  |
| Soltu.DM.01G035490 | homeobox | 5.74 | 8.57E-04 | 7.34 | 1.52E-07 | 7.87 | 4.11E-07 | 2.27 | 1.00E+00 | 6.25 | 1.26E-04 | 6.40 | 2.94E-06 |  |
| Soltu.DM.02G033970 | conserved hypothetical protein | 4.79 | 1.21E-03 | 6.66 | 1.94E-08 | 8.16 | 2.46E-10 | 0.48 | 1.00E+00 | 4.39 | 9.96E-04 | 4.66 | 2.80E-05 |  |
| Soltu.DM.10G028300 | nuclear factor Y, subunit A9 | 4.25 | 1.87E-02 | 6.57 | 1.05E-06 | 7.92 | 5.93E-08 | -0.87 | 1.00E+00 | 5.30 | 4.19E-04 | 6.34 | 3.65E-07 |  |
| Soltu.DM.08G010200***** | cytochrome P450, family 71, subfamily B, polypeptide | 4.18 | 1.53E-03 | 4.52 | 3.17E-05 | 3.50 | 4.35E-03 | 1.09 | 1.00E+00 | 3.28 | 2.55E-02 | 3.19 | 1.03E-02 |  |
|  | cytochrome P450, family 71, subfamily B, polypeptide |  |  |  |  |  |  |  |  |  |  |  |  |  |
|  | cytochrome P450, family 71, subfamily A, polypeptide |  |  |  |  |  |  |  |  |  |  |  |  |  |
| **Downregulated genes** |  |  |  |  |  |  |  |  |  |  |  |  |  |  |
| Soltu.DM.08G025250 | Bifunctional inhibitor/lipid-transfer protein/seed storage 2S | -4.26 | 3.20E-02 | -5.83 | 1.32E-04 | -5.47 | 1.11E-03 | 1.12 | 1.00E+00 | -3.36 | 9.62E-02 | -2.47 | 1.29E-01 |  |
|  | albumin superfamily protein |  |  |  |  |  |  |  |  |  |  |  |  |  |
| Soltu.DM.06G019440 | Bifunctional inhibitor/lipid-transfer protein/seed storage 2S  albumin superfamily protein | -2.19 | 4.61E-02 | -3.58 | 6.36E-06 | -3.31 | 2.05E-04 | -0.70 | 1.00E+00 | -4.16 | 3.31E-06 | -4.20 | 3.44E-08 |  |
|  |  |  |  |  |  |  |  |  |  |  |  |  |  |  |
|  |  |  |  |  |  |  |  |  |  |  |  |  |  |  |
| Soltu.DM.04G033210 | N-terminal nucleophile aminohydrolases | -3.14 | 3.82E-05 | -3.09 | 3.11E-06 | -1.91 | 1.33E-02 | -0.11 | 1.00E+00 | -0.34 | 7.69E-01 | -0.58 | 4.63E-01 |  |
|  | (Ntn hydrolases) superfamily protein |  |  |  |  |  |  |  |  |  |  |  |  |  |
| Soltu.DM.05G027180 | ABC-2 type transporter family protein | -2.75 | 1.50E-03 | -1.84 | 4.84E-03 | 0.28 | 7.03E-01 | -1.18 | NA | 0.00 | 9.99E-01 | 1.96 | 7.24E-05 |  |
| Soltu.DM.01G024030***** | basic helix-loop-helix (bHLH) DNA-binding superfamily protein | -2.46 | 1.34E-02 | -1.43 | 7.01E-02 | -0.38 | 7.09E-01 | -0.56 | 1.00E+00 | -0.16 | 9.01E-01 | -1.51 | 4.02E-02 |  |
| Soltu.DM.04G009990 | Heavy metal transport/detoxification | -2.40 | 1.70E-02 | -3.64 | 1.59E-06 | -3.53 | 3.14E-05 | -0.59 | 1.00E+00 | -3.05 | 7.15E-04 | -3.82 | 3.35E-07 |  |
|  | superfamily protein |  |  |  |  |  |  |  |  |  |  |  |  |  |
| Soltu.DM.06G027540 | Kinase interacting (KIP1-like) family protein | -2.35 | 4.71E-02 | -1.75 | 6.00E-02 | -0.76 | 5.09E-01 | -0.05 | NA | 0.18 | 9.14E-01 | 0.15 | 9.06E-01 |  |
| Soltu.DM.03G018250 | detoxifying efflux carrier | -2.27 | 4.14E-02 | -2.06 | 1.70E-02 | -0.87 | 4.13E-01 | -1.29 | 1.00E+00 | -1.36 | 2.06E-01 | -1.55 | 6.52E-02 |  |
| Soltu.DM.07G026560 | glycosyl hydrolase 9B8 | -2.05 | 1.21E-04 | -3.98 | 1.52E-16 | -4.12 | 1.58E-14 | -1.18 | 5.17E-01 | -2.86 | 1.97E-05 | -3.10 | 3.44E-08 |  |
| Soltu.DM.12G027980 | O-acyltransferase (WSD1-like) family protein | -2.00 | 2.79E-02 | -1.43 | 5.44E-02 | -1.34 | 1.07E-01 | -0.63 | 1.00E+00 | -1.42 | 1.05E-01 | -1.49 | 3.37E-02 |  |
| Soltu.DM.10G024900 | growth-regulating factor | -1.99 | 4.18E-02 | -1.63 | 3.22E-02 | -1.97 | 1.60E-02 | -0.90 | 1.00E+00 | -0.59 | 6.00E-01 | -1.34 | 8.94E-02 |  |
| Soltu.DM.02G025610 | conserved hypothetical protein | -1.96 | 4.95E-02 | 0.18 | 8.49E-01 | 0.54 | 5.75E-01 | -0.47 | 1.00E+00 | 0.90 | 3.70E-01 | 1.30 | 7.73E-02 |  |
| Soltu.DM.10G029820 | NAD(P)-binding Rossmann-fold superfamily protein | -1.96 | 3.85E-02 | -2.51 | 4.32E-04 | -2.63 | 7.74E-04 | -0.34 | 1.00E+00 | -1.16 | 2.37E-01 | -1.56 | 3.94E-02 |  |
|  |  |  |  |  |  |  |  |  |  |  |  |  |  |  |
| Soltu.DM.10G025190 | NAD(P)-binding Rossmann-fold superfamily protein | -1.79 | 4.18E-02 | -0.98 | 1.87E-01 | -1.06 | 1.89E-01 | -0.34 | 1.00E+00 | -1.18 | 1.73E-01 | -1.02 | 1.45E-01 |  |
| Soltu.DM.01G033740 | cytochrome P450, family 86, subfamily A, polypeptide | -1.96 | 2.64E-02 | -1.68 | 1.68E-02 | -1.77 | 2.15E-02 | -0.79 | 1.00E+00 | -2.38 | 2.45E-03 | -1.73 | 9.86E-03 |  |
| Soltu.DM.03G011660 | 3-ketoacyl-CoA synthase | -1.93 | 6.55E-04 | -1.66 | 5.56E-04 | -1.44 | 6.83E-03 | -0.70 | 1.00E+00 | -2.02 | 7.83E-03 | -1.61 | 6.22E-03 |  |
| Soltu.DM.03G036380***** | Glucose-methanol-choline (GMC) | -1.93 | 1.32E-02 | -2.71 | 5.11E-06 | -2.60 | 8.57E-05 | -0.29 | 1.00E+00 | -2.48 | 3.59E-04 | -2.61 | 6.70E-06 |  |
|  | oxidoreductase family protein |  |  |  |  |  |  |  |  |  |  |  |  |  |
| Soltu.DM.09G012800***** | Leucine-rich repeat protein kinase family protein | -1.90 | 2.24E-02 | -2.17 | 7.28E-04 | -2.60 | 1.99E-04 | 0.10 | 1.00E+00 | -2.52 | 4.93E-04 | -3.62 | 1.23E-09 |  |
| Soltu.DM.10G029690 | F-box family protein | -1.89 | 7.67E-07 | -3.23 | 2.44E-21 | -2.76 | 2.07E-13 | -0.65 | 9.73E-01 | -1.72 | 2.05E-05 | -1.87 | 4.06E-08 |  |
| Soltu.DM.09G028560***** | Chalcone and stilbene synthase family protein | -1.78 | 3.52E-02 | -0.90 | 2.16E-01 | -0.37 | 6.85E-01 | 0.22 | 1.00E+00 | -0.81 | 3.64E-01 | -1.06 | 1.09E-01 |  |

1. **Root, T2**

| **Potato genome v6.1 Gene ID** | **Potato genome v6.1  Gene Annotation** | **Root. Tolerant** | | | | | | **Root. Susceptible** | | | | | |  |
| --- | --- | --- | --- | --- | --- | --- | --- | --- | --- | --- | --- | --- | --- | --- |
|  |  | **T1** | | **T2** | | **T3** | | **T1** | | **T2** | | **T3** | |  |
|  |  | **LFC** | **padj** | **LFC** | **padj** | **LFC** | **padj** | **LFC** | **padj** | **LFC** | **padj** | **LFC** | **padj** |  |
| **Upregulated genes** |  |  |  |  |  |  |  |  |  |  |  |  |  |  |
| **Transcription factors** |  |  |  |  |  |  |  |  |  |  |  |  |  |  |
| Soltu.DM.12G007400 | WRKY DNA-binding protein | 4.12 | 5.28E-02 | 4.31 | 7.60E-03 | 7.68 | 7.54E-09 | 2.19 | NA | 1.11 | 2.01E-01 | 4.41 | 7.71E-14 |  |
| Soltu.DM.07G014750 | NAC domain containing protein | 3.55 | 1.87E-02 | 4.43 | 2.67E-04 | 2.64 | 3.02E-02 | -0.12 | NA | 1.04 | 3.67E-01 | -0.46 | 7.06E-01 |  |
| Soltu.DM.06G010320 | DRE-binding protein 2A | 2.26 | 1.41E-01 | 5.27 | 2.18E-07 | 4.68 | 3.60E-07 | 0.01 | NA | 1.07 | 7.37E-01 | 0.11 | 9.73E-01 |  |
| **Terpene biosynthesis** |  |  |  |  |  |  |  |  |  |  |  |  |  |  |
| Soltu.DM.01G040970 | terpene synthase | 3.47 | 2.54E-01 | 4.55 | 2.49E-02 | 5.01 | 4.75E-03 | 2.13 | NA | 0.68 | 7.97E-01 | -1.29 | 5.74E-01 |  |
| Soltu.DM.07G004480 | terpene synthase | 1.20 | 7.08E-01 | 5.46 | 2.78E-05 | 7.64 | 3.42E-11 | 2.01 | NA | 0.55 | 8.17E-01 | 1.03 | 5.40E-01 |  |
| **UDP-glycosyltransferases** |  |  |  |  |  |  |  |  |  |  |  |  |  |  |
| Soltu.DM.09G031020 | UDP-Glycosyltransferase superfamily protein | 5.58 | 3.27E-02 | 5.21 | 1.61E-02 | 6.20 | 1.04E-03 | -2.43 | NA | 2.67 | 1.97E-01 | 2.10 | 2.77E-01 |  |
| Soltu.DM.12G004300 | UDP-Glycosyltransferase superfamily protein | 0.15 | NA | 6.38 | 5.72E-03 | 7.07 | 4.91E-04 | 0.00 | NA | 0.11 | 9.80E-01 | 1.04 | 7.43E-01 |  |
| Soltu.DM.05G007640 | UDP-glucosyl transferase 74B1 | 2.60 | 2.00E-01 | 4.79 | 6.97E-04 | 5.02 | 2.81E-05 | 0.43 | 8.51E-01 | 2.41 | 9.11E-02 | 4.40 | 2.26E-04 |  |
| **Others** |  |  |  |  |  |  |  |  |  |  |  |  |  |  |
| Soltu.DM.04G020100 | non-photochemical quenching | 5.71 | NA | 5.62 | 6.58E-03 | 2.23 | 3.41E-01 | 0.58 | NA | -2.07 | 4.40E-01 | -1.22 | 5.89E-01 |  |
| Soltu.DM.09G023010 | Thioredoxin superfamily protein | 5.56 | 5.05E-02 | 4.96 | 3.48E-02 | 1.15 | 7.15E-01 | 2.08 | NA | 0.05 | 9.84E-01 | 1.69 | 3.50E-01 |  |
| Soltu.DM.02G027440 | phytosulfokine 6 precursor | 5.17 | 2.25E-03 | 5.08 | 8.85E-04 | 4.62 | 7.35E-04 | 0.78 | 6.72E-01 | 1.47 | 3.10E-01 | 3.46 | 1.96E-03 |  |
| Soltu.DM.01G006840 | NAD+ ADP-ribosyltransferases;NAD+ ADP-ribosyltransferases | 3.31 | NA | 4.95 | 1.87E-03 | 5.01 | 7.29E-04 | 0.57 | NA | 1.41 | 1.91E-01 | 1.44 | 1.31E-01 |  |
| Soltu.DM.12G009350 | fatty acid desaturase | 3.32 | 6.99E-02 | 4.79 | 4.29E-04 | 1.23 | 4.57E-01 | -1.73 | NA | 0.34 | 8.56E-01 | -3.67 | 3.20E-02 |  |
| Soltu.DM.01G005200 | beta-1,3-glucanase | 3.24 | 2.99E-01 | 4.58 | 1.85E-02 | 3.63 | 5.19E-02 | -0.54 | NA | 1.05 | 5.08E-01 | 0.49 | 7.48E-01 |  |
| Soltu.DM.03G013100 | 1-cysteine peroxiredoxin | 0.15 | NA | 4.30 | 3.26E-02 | 6.18 | 2.22E-04 | 0.82 | NA | 3.56 | 7.77E-02 | 4.30 | 1.43E-02 |  |
| Soltu.DM.04G019150 | cytochrome P450, family 76, subfamily C, polypeptide | 0.15 | NA | 4.99 | 4.54E-03 | 5.16 | 1.25E-03 | -0.52 | NA | 2.30 | 6.52E-02 | 1.51 | 2.25E-01 |  |
| Soltu.DM.04G001920 | hypothetical protein | 3.80 | 3.17E-01 | 5.00 | 4.40E-02 | 4.39 | 5.14E-02 | 1.19 | NA | 1.94 | 5.13E-01 | 5.75 | 4.75E-03 |  |
| Soltu.DM.06G019500 | hypothetical protein | 2.79 | 4.56E-02 | 4.87 | 6.90E-06 | 2.78 | 5.83E-03 | 2.09 | 8.80E-02 | 1.87 | 8.86E-02 | 1.01 | 3.58E-01 |  |
| Soltu.DM.01G007060 | hypothetical protein | 0.75 | NA | 4.75 | 8.36E-03 | 4.99 | 1.95E-03 | 0.95 | NA | 0.11 | 9.78E-01 | 0.11 | 9.75E-01 |  |
| Soltu.DM.08G024450 | hypothetical protein | 0.15 | 9.82E-01 | 6.26 | 2.94E-05 | 7.06 | 4.05E-07 | 0.98 | NA | 2.50 | 2.50E-01 | 1.80 | 3.95E-01 |  |
| **Downregulated genes** |  |  |  |  |  |  |  |  |  |  |  |  |  |  |
| **Cell wall modification** |  |  |  |  |  |  |  |  |  |  |  |  |  |  |
| Soltu.DM.02G001870 | Plant invertase/pectin methylesterase inhibitor superfamily | -0.95 | 4.12E-01 | -4.04 | 2.07E-09 | -0.63 | 3.85E-01 | -1.10 | 1.83E-01 | -0.94 | 2.14E-01 | 0.05 | 9.56E-01 |  |
| Soltu.DM.12G025120 | xyloglucan endotransglucosylase/hydrolase | -0.97 | 3.82E-01 | -3.60 | 6.53E-08 | -0.21 | 7.97E-01 | -0.99 | 2.37E-01 | -1.00 | 1.71E-01 | 0.13 | 8.72E-01 |  |
| Soltu.DM.01G006590 | root hair specific | -0.86 | 7.13E-01 | -5.99 | 2.90E-03 | -1.44 | 2.29E-01 | -1.68 | NA | -1.58 | 1.86E-01 | -0.87 | 4.41E-01 |  |
| Soltu.DM.04G014020 | Pectin lyase-like superfamily protein | -0.39 | NA | -3.54 | 1.74E-02 | -1.52 | 1.05E-01 | -1.00 | NA | 0.53 | 7.29E-01 | -0.87 | 5.27E-01 |  |
| **Others** |  |  |  |  |  |  |  |  |  |  |  |  |  |  |
| Soltu.DM.10G029820 | NAD(P)-binding Rossmann-fold superfamily protein | -2.33 | NA | -4.72 | 1.81E-02 | -5.00 | 1.91E-03 | -1.71 | NA | -3.18 | 6.91E-02 | -4.09 | 1.41E-02 |  |
|  |  |  |  |  |  |  |  |  |  |  |  |  |  |  |
| Soltu.DM.06G017710 | Serine hydrolase (FSH1) domain containing protein | -1.47 | NA | -4.37 | 2.20E-02 | -1.57 | 1.88E-01 | -0.26 | NA | -0.09 | 9.44E-01 | -0.24 | 7.89E-01 |  |
| Soltu.DM.10G004610 | HXXXD-type acyl-transferase family protein | -1.59 | 2.45E-01 | -5.87 | 9.92E-04 | -1.79 | 3.81E-02 | -1.10 | NA | -0.01 | 9.94E-01 | -0.21 | 8.34E-01 |  |
| Soltu.DM.01G005700 | HXXXD-type acyl-transferase family protein | -0.80 | 3.60E-01 | -3.99 | 1.01E-09 | -1.09 | 3.18E-02 | -0.64 | 3.68E-01 | -0.84 | 1.62E-01 | -0.56 | 3.19E-01 |  |
| Soltu.DM.01G006560 | Peroxidase superfamily protein | -0.75 | 6.61E-01 | -3.81 | 2.02E-04 | -0.51 | 6.07E-01 | -1.18 | 2.70E-01 | -0.99 | 3.10E-01 | 0.16 | 8.75E-01 |  |
| Soltu.DM.03G007520 | Protein kinase superfamily protein | -0.67 | 7.88E-01 | -4.68 | 1.89E-02 | -2.35 | 8.61E-02 | -0.36 | NA | -0.23 | 8.70E-01 | -0.69 | 4.97E-01 |  |
| Soltu.DM.03G025430 | cytochrome P450, family 71, subfamily A, polypeptide | -0.43 | NA | -3.85 | 4.54E-02 | -1.40 | 2.16E-01 | -0.28 | NA | -0.91 | 5.15E-01 | -1.53 | 1.93E-01 |  |
| Soltu.DM.08G026520 | Subtilase family protein | -0.30 | NA | -4.68 | 2.85E-02 | -0.17 | 9.22E-01 | -0.74 | NA | -2.69 | 9.15E-02 | -1.19 | 3.71E-01 |  |
| Soltu.DM.01G000610 | S-adenosyl-L-methionine-dependent methyltransferases superfamily protein | 0.30 | NA | -3.83 | 3.90E-02 | -2.46 | 3.11E-02 | 0.08 | NA | -0.69 | 6.04E-01 | -0.59 | 6.01E-01 |  |
| Soltu.DM.09G009180 | wall-associated kinase | -0.29 | NA | -4.70 | 1.59E-02 | -1.14 | 3.29E-01 | 0.53 | NA | -0.66 | 6.18E-01 | -1.90 | 9.59E-02 |  |
| Soltu.DM.01G028400 | Cysteine/Histidine-rich C1 domain family protein | 0.05 | 9.81E-01 | -4.42 | 7.31E-05 | -2.97 | 1.84E-05 | -0.15 | NA | -2.62 | 9.54E-02 | -2.73 | 4.11E-02 |  |
| Soltu.DM.06G001160 | Protein of unknown function, DUF547 | -0.47 | 6.91E-01 | -3.61 | 1.14E-08 | -0.75 | 1.99E-01 | -0.24 | 7.94E-01 | -0.39 | 6.00E-01 | 0.12 | 8.66E-01 |  |
| Soltu.DM.10G005090 | Organ specific protein domain containing protein | -0.02 | 9.93E-01 | -4.78 | 1.35E-02 | -1.56 | 1.61E-01 | -0.28 | NA | -0.01 | 9.94E-01 | -0.83 | 4.54E-01 |  |
| Soltu.DM.02G009560 | conserved hypothetical protein | -2.38 | NA | -3.74 | 1.80E-02 | -1.91 | 6.25E-02 | -2.14 | NA | -1.81 | 1.80E-01 | -2.76 | 3.60E-02 |  |
| Soltu.DM.01G046840 | conserved hypothetical protein | -0.56 | NA | -5.42 | 4.74E-03 | -1.02 | 3.39E-01 | -0.45 | NA | -1.89 | 1.74E-01 | -0.87 | 4.56E-01 |  |
| SoltuDM.06G003740 | hypothetical protein | 0.23 | NA | -3.99 | 2.72E-02 | -1.25 | 1.81E-01 | -0.42 | NA | -1.44 | 2.17E-01 | -1.69 | 1.03E-01 |  |

1. **Leaf, T2**

| **Potato genome v6.1  Gene ID** | **Potato genome v6.1  Gene Annotation** | **Leaf.Tolerant** | | | | | | **Leaf.Susceptible** | | | | | |  |
| --- | --- | --- | --- | --- | --- | --- | --- | --- | --- | --- | --- | --- | --- | --- |
|  |  | **T1** | | **T2** | | **T3** | | **T1** | | **T2** | | **T3** | |  |
|  |  | **LFC** | **padj** | **LFC** | **padj** | **LFC** | **padj** | **LFC** | **padj** | **LFC** | **padj** | **LFC** | **padj** |  |
| **Upregulated genes** |  |  |  |  |  |  |  |  |  |  |  |  |  |  |
| **Transport** |  |  |  |  |  |  |  |  |  |  |  |  |  |  |
| Soltu.DM.03G008090 | Transmembrane amino acid transporter family protein | 2.03 | NA | 6.86 | 7.53E-05 | 6.73 | 1.71E-04 | -0.02 | NA | 3.48 | 1.28E-01 | 4.83 | 6.30E-03 |  |
|  |  |  |  |  |  |  |  |  |  |  |  |  |  |  |
| Soltu.DM.10G018680 | lipid transfer protein | 5.29 | 2.22E-03 | 5.62 | 7.31E-05 | 6.05 | 6.10E-05 | 0.16 | 1.00E+00 | 1.95 | 2.30E-01 | 2.76 | 2.47E-02 |  |
| **Heat shock proteins** |  |  |  |  |  |  |  |  |  |  |  |  |  |  |
| Soltu.DM.04G028280 | heat shock protein 18.2 | 1.45 | 7.27E-01 | 5.92 | 1.73E-02 | 7.63 | 2.70E-03 | 0.50 | 1.00E+00 | 4.12 | 1.45E-01 | 7.97 | 1.92E-04 |  |
| **Others** |  |  |  |  |  |  |  |  |  |  |  |  |  |  |
| Soltu.DM.06G018480 | Late embryogenesis abundant protein, group 1 protein | 4.16 | NA | 6.13 | 1.46E-02 | 6.56 | 1.28E-02 | -2.84 | NA | -2.88 | 4.42E-01 | -0.30 | 9.28E-01 |  |
| Soltu.DM.03G030490 | late embryogenesis abundant protein-related / LEA protein-related | -0.21 | NA | 5.07 | 1.52E-02 | 6.67 | 1.25E-03 | 0.00 | NA | 4.49 | 5.95E-02 | 6.89 | 2.69E-04 |  |
| Soltu.DM.03G010080 | Late Embryogenesis Abundant 4-5 | 1.50 | NA | 5.06 | 1.09E-02 | 6.10 | 2.12E-03 | -0.98 | NA | 3.60 | 1.25E-01 | 5.76 | 1.62E-03 |  |
| Soltu.DM.04G002580 | Heavy metal transport/detoxification superfamily protein | 3.29 | NA | 5.70 | 8.91E-03 | 4.31 | 7.27E-02 | 0.01 | NA | 2.83 | 3.33E-01 | 4.37 | 4.45E-02 |  |
| Soltu.DM.09G027510 | AWPM-19-like family protein | 1.70 | NA | 5.69 | 5.45E-03 | 5.74 | 6.86E-03 | -1.24 | NA | 2.62 | 2.12E-01 | 3.80 | 1.78E-02 |  |
| Soltu.DM.06G033600 | nuclear transport factor 2 (NTF2) family protein / RNA recognition motif (RRM)-containing protein | 4.47 | 2.26E-01 | 5.66 | 4.26E-02 | 7.69 | 6.73E-03 | 2.20 | NA | 5.14 | 9.07E-02 | 6.12 | 1.27E-02 |  |
| Soltu.DM.09G021850 | Pollen Ole e 1 allergen and extensin family protein | 1.49 | 7.27E-01 | 5.63 | 2.38E-02 | 7.16 | 4.71E-03 | 0.39 | 1.00E+00 | 0.53 | 8.75E-01 | -0.20 | 9.41E-01 |  |
| Soltu.DM.08G017870 | Pyridoxal phosphate (PLP)-dependent transferases superfamily protein | 3.86 | 5.82E-02 | 5.55 | 1.57E-04 | 7.03 | 1.46E-06 | -0.51 | NA | -1.14 | 4.38E-01 | 1.88 | 3.84E-02 |  |
| Soltu.DM.08G017880 | Pyridoxal phosphate (PLP)-dependent transferases superfamily protein | 3.84 | NA | 5.17 | 2.55E-03 | 6.63 | 8.19E-05 | 0.47 | NA | 0.18 | 9.35E-01 | 3.41 | 1.22E-03 |  |
| Soltu.DM.08G028850 | WRKY family transcription factor | 3.59 | 2.99E-04 | 5.36 | 2.17E-11 | 5.17 | 6.74E-09 | 1.84 | 6.23E-01 | 0.30 | 8.34E-01 | 5.05 | 3.10E-11 |  |
| Soltu.DM.10G006090 | Bifunctional inhibitor/lipid-transfer protein/seed storage 2S albumin superfamily protein | 5.01 | NA | 5.30 | 2.44E-02 | 6.82 | 4.02E-03 | -0.84 | NA | 1.27 | 7.25E-01 | 1.80 | 4.75E-01 |  |
| Soltu.DM.02G014830 | Protein of unknown function (DUF1264) | 2.97 | NA | 5.37 | 1.01E-02 | 6.14 | 3.73E-03 | -1.80 | NA | 3.87 | 9.10E-02 | 5.55 | 2.49E-03 |  |
| Soltu.DM.10G023160 | Protein of unknown function (DUF668) | 1.93 | 2.60E-01 | 5.20 | 1.12E-05 | 6.33 | 8.47E-07 | -0.75 | 1.00E+00 | 1.12 | 5.42E-01 | 4.82 | 2.72E-05 |  |
| Soltu.DM.08G029210 | hypothetical protein | 3.91 | 1.07E-01 | 7.69 | 1.48E-06 | 7.45 | 6.28E-06 | 0.22 | NA | 2.74 | 5.67E-02 | 4.29 | 1.61E-04 |  |
| Soltu.DM.01G007060 | hypothetical protein | 3.89 | NA | 6.28 | 2.04E-03 | 6.78 | 1.28E-03 | -0.98 | NA | -1.01 | 8.06E-01 | -1.07 | 7.26E-01 |  |
| Soltu.DM.12G009200 | conserved hypothetical protein | 2.96 | NA | 6.80 | 3.58E-03 | 6.08 | 1.53E-02 | -2.03 | NA | 3.83 | 1.57E-01 | 5.17 | 1.55E-02 |  |
| Soltu.DM.03G031190 | hypothetical protein | 4.07 | NA | 5.13 | 1.75E-03 | 5.56 | 5.98E-04 | -1.87 | NA | 1.78 | NA | 2.48 | 4.08E-02 |  |
| **Downregulated genes** |  |  |  |  |  |  |  |  |  |  |  |  |  |  |
| **Cell wall modification** |  |  |  |  |  |  |  |  |  |  |  |  |  |  |
| Soltu.DM.02G024320 | Pectin lyase-like superfamily protein | -2.69 | 1.17E-01 | -3.36 | 9.67E-03 | -1.41 | 3.67E-01 | -0.01 | 1.00E+00 | -1.62 | 3.40E-01 | -1.80 | 1.67E-01 |  |
| **Others** |  |  |  |  |  |  |  |  |  |  |  |  |  |  |
| Soltu.DM.08G025250 | Bifunctional inhibitor/lipid-transfer protein/seed storage 2S albumin superfamily protein | -4.26 | 3.20E-02 | -5.83 | 1.32E-04 | -5.47 | 1.11E-03 | 1.12 | 1.00E+00 | -3.36 | 9.62E-02 | -2.47 | 1.29E-01 |  |
| Soltu.DM.10G020770 | myb domain protein | -3.02 | 8.13E-02 | -5.00 | 3.93E-04 | -3.13 | 3.01E-02 | 0.37 | NA | -0.96 | 6.25E-01 | -2.47 | 7.36E-02 |  |
| Soltu.DM.11G019840 | cytochrome P450, family 716, subfamily A, polypeptide | -1.21 | 2.29E-01 | -4.92 | 2.35E-04 | -3.50 | 6.13E-04 | -0.36 | 1.00E+00 | -0.87 | 3.16E-01 | -2.13 | 9.00E-04 |  |
| Soltu.DM.06G015970 | Plant stearoyl-acyl-carrier-protein desaturase family protein | -2.94 | NA | -4.65 | 3.03E-02 | -0.99 | 6.66E-01 | 0.15 | NA | -0.77 | NA | -0.85 | 6.47E-01 |  |
| Soltu.DM.04G019810 | GDSL-like Lipase/Acylhydrolase superfamily protein | -3.66 | NA | -4.60 | 4.24E-02 | -1.05 | 7.06E-01 | 1.28 | NA | -0.52 | 8.90E-01 | -2.11 | 4.14E-01 |  |
| Soltu.DM.10G015940 | SAUR-like auxin-responsive protein family | -1.07 | NA | -4.52 | 1.08E-04 | -3.95 | 2.64E-04 | -0.33 | NA | -3.65 | 6.33E-02 | -4.15 | 7.28E-03 |  |
| Soltu.DM.03G011570 | HXXXD-type acyl-transferase family protein | -0.89 | NA | -4.36 | 2.13E-03 | -2.26 | 3.25E-02 | 0.12 | NA | -0.81 | 5.37E-01 | -2.40 | 1.99E-02 |  |
| Soltu.DM.07G021960 | protodermal factor | -4.01 | 7.11E-02 | -3.95 | 2.11E-02 | -2.21 | 2.87E-01 | 0.37 | 1.00E+00 | -2.58 | 2.36E-01 | -2.66 | 1.22E-01 |  |
| Soltu.DM.06G032220 | Undecaprenyl pyrophosphate synthetase family protein | -1.71 | NA | -3.57 | 1.17E-02 | -0.55 | 7.57E-01 | 1.28 | NA | -0.54 | 8.25E-01 | -0.03 | 9.88E-01 |  |
| Soltu.DM.01G035840 | RING/U-box superfamily protein | -1.48 | NA | -3.44 | 7.24E-03 | -1.95 | 7.04E-02 | 0.19 | NA | -0.87 | 4.95E-01 | -1.94 | 4.84E-02 |  |
| Soltu.DM.09G020340 | heat shock transcription factor A6B | 1.18 | 4.38E-01 | -3.38 | 1.73E-03 | -5.26 | 7.58E-06 | 1.13 | 1.00E+00 | -0.12 | 9.49E-01 | -2.90 | 5.61E-03 |  |
| Soltu.DM.10G000470 | terpene synthase | -0.90 | NA | -3.35 | 4.56E-02 | -0.79 | 5.44E-01 | -0.32 | NA | -1.46 | 7.03E-02 | -3.07 | 3.26E-05 |  |
| Soltu.DM.02G029200 | NAD(P)-binding Rossmann-fold superfamily protein | -2.00 | NA | -3.26 | 4.61E-03 | -2.11 | 6.92E-02 | 0.30 | NA | -2.29 | 1.80E-01 | -1.73 | 1.65E-01 |  |
| Soltu.DM.01G044230 | Chloroplast-targeted copper chaperone protein | -2.55 | NA | -3.18 | 1.59E-02 | -2.33 | 9.27E-02 | -0.27 | NA | -3.82 | 9.20E-02 | -2.85 | 6.23E-02 |  |
| Soltu.DM.04G028960 | sulfate transporter 1;3 | -0.78 | NA | -3.17 | 3.35E-02 | -2.02 | 1.09E-01 | 0.36 | NA | -0.08 | 9.55E-01 | -1.35 | 1.48E-01 |  |
| Soltu.DM.03G035650 | Protein of unknown function (DUF1677) | -0.87 | 4.75E-01 | -3.32 | 1.64E-04 | -5.20 | 1.90E-06 | -0.63 | 1.00E+00 | -1.21 | 2.80E-01 | -3.71 | 1.16E-05 |  |
| Soltu.DM.05G015980 | hypothetical protein | -0.93 | NA | -3.75 | 3.05E-02 | -1.81 | 2.94E-01 | 0.19 | NA | 0.54 | 5.97E-01 | 0.41 | 6.15E-01 |  |
| Soltu.DM.07G027150 | conserved hypothetical protein | 0.63 | 6.30E-01 | -3.21 | 1.40E-02 | -1.55 | 1.79E-01 | -0.40 | 1.00E+00 | -0.19 | 8.91E-01 | 0.09 | 9.30E-01 |  |
| Soltu.DM.08G020230 | hypothetical protein | -1.79 | NA | -3.16 | 9.65E-03 | -2.40 | 4.81E-02 | -0.49 | NA | 0.58 | 7.48E-01 | -0.03 | 9.87E-01 |  |

**Supplementary Table 4. Top 20 most up- or down- regulated genes in the early and late responses to drought in only the tolerant variety.** Differentially Expressed Genes (DEGs) are defined by log2FC >1 and padj. < 0.05 compared with the non-stressed control condition and are shown for the early response (T1) in root **(A)** or leaf **(B)** and in the late response (T2) in root **(C)** or leaf **(D)**. An asterisk denotes DEGs whose expression is significantly higher (or lower) in the tolerant than in the susceptible variety for the upregulated (or downregulated) genes at T1. In grey are the padj. values > 0.05. Expression of the DEGs is also indicated for the recovery phase, T3. Genes referred to in the main text are shown in red font.

| **Potato genome v6.1 Gene ID** | ***Potato genome v6.1 Gene Annotation*** | **Leaf.Tolerant** | | | | | | **Leaf.Susceptible** | | | | | |
| --- | --- | --- | --- | --- | --- | --- | --- | --- | --- | --- | --- | --- | --- |
|  |  | **T1** | | **T2** | | **T3** | | **T1** | | **T2** | | **T3** | |
|  |  | **log2FC** | **padj** | **log2FC** | **padj** | **log2FC** | **padj** | **log2FC** | **padj** | **log2FC** | **padj** | **log2FC** | **padj** |
| Soltu.DM.01G050980 | subtilisin-like serine protease | -0.09 | 8.59E-01 | 0.08 | 8.36E-01 | 1.11 | 4.23E-04 | -0.06 | 1.00E+00 | 0.6 | 9.85E-02 | 0.99 | 3.59E-04 |
| Soltu.DM.03G015440 | Plant invertase/pectin methylesterase inhibitor superfamily protein | 2.4 | 4.25E-05 | 5.00 | 4.61E-26 | 4.76 | 3.71E-19 | 2.16 | 2.28E-03 | 5.49 | 5.13E-24 | 5.45 | 1.53E-29 |
| Soltu.DM.03G015460 | Plant invertase/pectin methylesterase inhibitor superfamily protein | 3.42 | 2.48E-02 | 6.86 | 1.03E-10 | 6.66 | 1.05E-09 | 3.35 | 6.82E-01 | 6.18 | 3.98E-05 | 6.75 | 1.24E-06 |
| Soltu.DM.03G015480 | Plant invertase/pectin methylesterase inhibitor superfamily protein | 1.82 | 2.79E-03 | 3.73 | 4.64E-16 | 3.24 | 4.53E-10 | 2.8 | 8.72E-05 | 6.3 | 2.40E-27 | 6.11 | 2.16E-30 |
| Soltu.DM.03G015490 | Plant invertase/pectin methylesterase inhibitor superfamily protein | 3.21 | 2.41E-04 | 5.74 | 1.57E-16 | 5.6 | 6.63E-13 | 2.48 | 5.33E-02 | 5.76 | 2.62E-13 | 6.46 | 5.47E-21 |
| Soltu.DM.03G015500 | Plant invertase/pectin methylesterase inhibitor superfamily protein | 3.17 | 3.32E-05 | 4.51 | 1.20E-12 | 3.71 | 1.76E-07 | 1.78 | 4.60E-01 | 5.16 | 1.01E-12 | 5.71 | 6.09E-19 |
| Soltu.DM.03G015510 | Plant invertase/pectin methylesterase inhibitor superfamily protein | 2.9 | 5.30E-09 | 5.13 | 8.02E-32 | 4.56 | 2.29E-20 | 1.98 | 3.29E-03 | 5.55 | 1.59E-28 | 6.08 | 2.12E-42 |
| Soltu.DM.03G015520 | Plant invertase/pectin methylesterase inhibitor superfamily protein | 1.55 | 1.67E-01 | 2.8 | 3.39E-04 | 1.06 | 2.92E-01 | 1.26 | 1.00E+00 | 4.18 | 1.82E-06 | 4.88 | 1.27E-10 |
| Soltu.DM.10G026840 | Plant protein of unknown function (DUF828) | -1.00 | 2.72E-02 | -1.4 | 4.19E-05 | -1.00 | 1.01E-02 | -0.29 | 1.00E+00 | -0.74 | 9.27E-02 | -1.44 | 1.94E-05 |

Supplementary Table 5. Genes inside the plant cell wall modification term (GO:0009827) differentially expressed in any time point (T1, T2 or T3) in the leaf of the susceptible or tolerant variety. Values in grey denote padj. > 0.05.

| **Potato genome v6.1  Gene ID** | **Potato genome v6.1  Gene Annotation** | **Leaf.Tolerant** | | | **Leaf.Susceptible** | | | **Root.Tolerant** | | | **Root.Susceptible** | | | ***A. thaliana* GeneName** |
| --- | --- | --- | --- | --- | --- | --- | --- | --- | --- | --- | --- | --- | --- | --- |
|  |  | **T1** | **T2** | **T3** | **T1** | **T2** | **T3** | **T1** | **T2** | **T3** | **T1** | **T2** | **T3** |  |
| Soltu.DM.11G011430 | ATP-binding cassette family G25 | 1.48 | 2.26 | 2.74 | - | 1.74 | 1.66 | - | 2.84 | 1.45 | - | 1.73 | - | ABCG25 |
| Soltu.DM.05G023720 | pleiotropic drug resistance | 1.21 | 1.89 | 1.76 | - | - | 1.56 | - | - | 1.22 | - | 1.05 | 1.10 | ABCG40 |
| Soltu.DM.04G000490 | ABI five binding protein | 3.42 | 4.44 | 5.17 | - | 4.07 | 4.13 | - | 6.82 | 6.16 | - | 5.98 | 5.00 | AFP1 |
| Soltu.DM.02G030840 | ABI five binding protein | 3.65 | 3.63 | 5.10 | - | 3.99 | 4.67 | - | 2.51 | 1.96 | - | 2.43 | 1.78 | AFP3 |
| Soltu.DM.05G000860 | ABI five binding protein | 2.98 | 4.42 | 5.54 | - | 3.81 | 3.99 | - | 4.60 | 4.43 | - | 3.40 | 3.07 | AFP3 |
| Soltu.DM.08G001760 | 2-oxoglutarate (2OG) and Fe(II)-dependent oxygenase superfamily protein | 1.60 | 2.40 | 1.64 | - | - | 1.17 | - | - | 3.68 | - | 3.34 | 3.14 | AT2G36690 |
| Soltu.DM.02G017790 | Aluminium induced protein with YGL and LRDR motifs | 1.77 | 2.30 | 2.27 | - | 1.63 | 2.17 | - | 2.25 | 1.77 | - | - | - | AT4G27450 / AT3G22850 |
| Soltu.DM.01G035490 | homeobox | 5.74 | 7.34 | 7.87 | - | 6.25 | 6.40 | - | 6.01 | 4.19 | - | 3.61 | 2.18 | ATHB-12 |
| Soltu.DM.06G014070 | homeobox | 2.68 | 3.19 | 3.07 | - | 3.26 | 2.29 | - | 1.47 | - | - | 2.58 | - | ATHB-7 |
| Soltu.DM.11G022320 | BEL1-like homeodomain | 1.61 | 2.01 | 1.77 | - | 1.95 | 1.95 | 0.92 | 1.54 | - | - | 1.06 | - | BLH1 |
| Soltu.DM.06G026950 | BTB and TAZ domain protein | 1.74 | - | - | - | - | - | 0.77 | 1.24 | 1.21 | 0.68 | 0.97 | 1.41 | BT2 |
| Soltu.DM.02G032680 | Peroxidase superfamily protein | 1.41 | - | - | - | - | -1.13 | - | - | - | - | - | - | DOX1 |
| Soltu.DM.06G010320 | DRE-binding protein 2A | 2.07 | - | - | - | - | - | - | 5.27 | 4.68 | - | - | - | DREB2C |
| Soltu.DM.04G031660 | MATE efflux family protein | 2.20 | 2.30 | - | - | - | 1.60 | - | 3.49 | 1.27 | 1.41 | 3.52 | 2.28 | DTX48 |
| Soltu.DM.06G034820 | EID1-like | 2.32 | - | - | - | 2.39 | - | 3.23 | 5.07 | 2.80 | 2.75 | 4.22 | 2.19 | EDL3 |
| Soltu.DM.10G005000 | erf domain protein | 2.80 | 2.74 | 2.98 | - | 2.19 | 2.90 | 1.73 | 2.28 | 1.89 | 1.63 | - | 1.68 | ERF4 |
| Soltu.DM.01G050660 | GAST1 protein homolog | 1.82 | 2.03 | - | - | - | - | - | - | - | - | - | - | GASA1 |
| Soltu.DM.05G019830 | G-box binding factor | 3.59 | 4.65 | 5.61 | - | 3.94 | 4.77 | - | 2.93 | 3.13 | - | 2.67 | 3.07 | GBF3 |
| Soltu.DM.01G007150 | CAP160 protein | 5.91 | 8.95 | 10.45 | - | 4.80 | 6.30 | - | - | 4.98 | - | - | - | LTI65 |
| Soltu.DM.01G045280 | nicotinamidase | 1.09 | 0.95 | - | - | 1.05 | 1.21 | - | - | - | - | - | - | NIC1 |
| Soltu.DM.09G026670 | phosphate transporter 1;4 | 1.31 | 1.77 | 2.02 | - | 1.02 | 1.02 | - | - | - | - | -1.37 | -0.99 | PHT1-4 |
| Soltu.DM.06G013730 | protein phosphatase 2CA | 6.20 | 4.36 | 5.45 | - | 3.65 | - | - | 7.72 | 5.90 | - | 8.33 | 6.10 | PP2CA |
| Soltu.DM.03G012480 | protein phosphatase 2CA | 4.56 | 6.39 | 7.33 | - | 6.16 | 6.57 | - | - | - | - | - | - | PP2CA |
| Soltu.DM.05G023010 | protein phosphatase 2CA | 2.64 | 2.63 | 2.99 | - | 2.80 | 2.45 | 1.51 | 3.37 | 1.22 | 1.75 | 3.08 | 1.19 | PP2CA |
| Soltu.DM.03G022710 | highly ABA-induced PP2C gene | 3.78 | 5.65 | 6.73 | - | 4.93 | 5.05 | - | 7.10 | 6.73 | - | 6.21 | 5.43 | AIP1 / PP2CA |
| Soltu.DM.07G012130 | Protein phosphatase 2C family protein | 1.47 | 2.28 | 2.54 | - | 2.00 | 2.04 | - | 2.91 | 2.41 | - | 2.21 | 1.49 | ABI2 |
| Soltu.DM.12G029330 | NAC (No Apical Meristem) domain transcriptional regulator superfamily protein | 3.38 | 4.37 | 4.83 | - | 3.55 | 3.38 | 2.95 | 5.18 | 3.29 | 2.65 | 5.08 | 3.29 | RD26 |
| Soltu.DM.07G024710 | NAC (No Apical Meristem) domain transcriptional regulator superfamily protein | 3.13 | 5.52 | 6.16 | - | 5.25 | 5.94 | 1.32 | 4.52 | 2.48 | 1.63 | 4.34 | 2.57 | RD26 |
| Soltu.DM.09G024150 | Major facilitator superfamily protein | 1.09 | 2.01 | 1.60 | - | 1.44 | 1.63 | - | 2.64 | 3.09 | - | - | 2.36 | STP13 |
| Soltu.DM.01G048750 | extra-large G-protein | 1.99 | 3.57 | 3.51 | - | 1.43 | 3.48 | - | - | 2.52 | - | - | 1.71 | XLG1 |
| Soltu.DM.12G006170 | zinc-finger protein | 1.90 | 2.02 | 3.50 | - | - | 1.88 | 2.94 | - | 2.15 | 3.29 | -1.82 | - | ZAT10 |
| Soltu.DM.08G015120 | carotenoid cleavage dioxygenase | 1.24 | 1.51 | 1.02 | - | - | - | - | - | - | - | - | - | NCED1 / NCED3 |
| Soltu.DM.05G021330 | white-brown complex homolog protein | - | - | 1.96 | - | - | - | 1.46 | 1.84 | 2.25 | - | - | 1.72 | ABCG11 |
| Soltu.DM.05G021350 | white-brown complex homolog protein | - | - | - | - | - | - | 1.39 | 1.84 | 2.15 | - | 1.03 | 1.78 | ABCG11 |
| Soltu.DM.05G021360 | white-brown complex homolog protein | - | - | - | - | - | - | 1.71 | 1.93 | 2.45 | - | 0.98 | 1.75 | ABCG11 |
| Soltu.DM.05G023730 | pleiotropic drug resistance | - | 1.64 | - | - | - | - | 1.27 | 1.09 | 1.25 | - | - | - | ABCG40 |
| Soltu.DM.09G028710 | pleiotropic drug resistance | - | - | - | - | - | - | 1.18 | 0.97 | 1.60 | - | - | 1.14 | ABCG40 |
| Soltu.DM.11G004860 | 2-oxoglutarate (2OG) and Fe(II)-dependent oxygenase superfamily protein | - | - | - | - | - | - | 2.40 | - | - | - | - | -2.89 | AT2G36690 |
| Soltu.DM.05G023310 | myb domain protein | - | - | - | - | - | - | 5.21 | 5.68 | 6.37 | - | 3.10 | 4.27 | AtMYB78 |
| Soltu.DM.12G001820 | myb domain protein | - | - | - | - | - | - | 6.44 | - | 9.26 | - | - | 6.03 | AtMYB78 |
| Soltu.DM.02G019660 | cysteine-rich RLK (RECEPTOR-like protein kinase) | - | - | - | - | - | - | 4.00 | 4.05 | 4.25 | - | - | - | CRK29 |
| Soltu.DM.12G023090 | Receptor-like protein kinase-related family protein | - | - | - | - | - | - | 2.19 | - | - | - | - | - | CRRSP38 |
| Soltu.DM.03G001930 | ethylene responsive element binding factor | - | 2.19 | 2.85 | - | 3.83 | 4.18 | 1.86 | 3.71 | 2.57 | - | 2.57 | 1.90 | ERF4 |
| Soltu.DM.10G020680 | GRAS family transcription factor family protein | - | - | 2.70 | - | - | 1.49 | 2.53 | 2.52 | 3.83 | - | - | - | GAI |
| Soltu.DM.02G006360 | galactinol synthase | - | - | 5.31 | - | 4.50 | 5.08 | 4.74 | 7.13 | 7.43 | - | 4.01 | 5.16 | GOLS1 |
| Soltu.DM.08G010990 | lipoxygenase | - | - | - | - | - | - | 1.85 | 1.95 | 2.47 | - | 1.25 | 1.81 | LOX1 |
| Soltu.DM.07G017180 | mitogen-activated protein kinase kinase kinase | - | 2.40 | 3.77 | - | - | 3.39 | 1.51 | 4.79 | 3.02 | - | 3.59 | 2.25 | MAPKKK17 |
| Soltu.DM.02G008230 | PATATIN-like protein | - | - | - | - | - | - | 4.06 | - | 3.57 | - | - | - | PLP3 |
| Soltu.DM.02G006090 | BURP domain-containing protein | - | - | - | - | - | - | 1.89 | - | - | - | - | -0.95 | RD22 |
| Soltu.DM.02G033230 | Raffinose synthase family protein | - | 7.15 | 8.14 | - | 4.07 | 5.06 | 1.57 | 6.94 | 8.22 | - | 4.98 | 5.57 | RFS5 |
| Soltu.DM.10G020700 | GRAS family transcription factor family protein | - | 2.38 | 3.05 | - | - | 2.36 | 1.26 | 1.05 | 3.84 | - | - | 1.90 | RGL1 |
| Soltu.DM.08G030040 | homeobox | 3.50 | 5.61 | 6.07 | - | 5.31 | 5.93 | 2.33 | 6.03 | 4.75 | - | 5.26 | 3.73 | ATHB-7 |

Supplementary Table 6. DEGs inside the response to abscisic acid term (GO:0009737) unique to the early response of the tolerant variety. Shown are the log2FC values for the DEGs that are only upregulated in the tolerant leaves (green) or only in the tolerant roots (yellow), or only in both tissues (orange), but not in the susceptible during the early response to drought. (-) denotes genes that did not have a significant change from control to the respective time point. Genes referred to in the main text are shown in red font.

| **Potato genome v6.1  Gene ID** | **Potato genome v6.1  Gene Annotation** | **Root.Tolerant** | | | **Root.Susceptible** | | | ***A. thaliana* Gene Name** |
| --- | --- | --- | --- | --- | --- | --- | --- | --- |
|  |  | **T1** | **T2** | **T3** | **T1** | **T2** | **T3** |  |
| Soltu.DM.03G003070 | serine-type endopeptidase inhibitors | 6.45 | - | - | - | - | - | AT1G72060 |
| Soltu.DM.06G018620 | serine-type endopeptidase inhibitors | 5.33 | - | - | - | - | - | AT1G72060 |
| Soltu.DM.06G032730 | Peroxidase superfamily protein | 4.93 | - | - | - | - | - | PER52 |
| Soltu.DM.02G006360 | galactinol synthase | 4.74 | 7.13 | 7.43 | - | 4.01 | 5.16 | GOLS1 |
| Soltu.DM.06G018610 | serine-type endopeptidase inhibitors | 4.63 | - | - | - | - | - | AT1G72060 |
| Soltu.DM.02G023240 | Rhodanese/Cell cycle control phosphatase superfamily protein | 2.53 | 1.73 | - | - | 1.80 | - | STR15 |
| Soltu.DM.01G040810 | Peroxidase superfamily protein | 2.24 | - | 3.73 | - | - | 3.53 | PER28 |
| Soltu.DM.01G000790 | PLANT CADMIUM RESISTANCE | 1.96 | - | 2.28 | - | - | - | PCR2 |
| Soltu.DM.03G030760 | blue-copper-binding protein | 1.85 | 1.93 | - | - | - | 1.80 | BCB |
| Soltu.DM.01G011520 | Peroxidase superfamily protein | 1.62 | - | - | - | -1.78 | -1.46 | PER59 |
| Soltu.DM.02G033230 | Raffinose synthase family protein | 1.57 | 6.94 | 8.22 | - | 4.98 | 5.57 | RFS5 |
| Soltu.DM.06G031880 | HSP20-like chaperones superfamily protein | 1.54 | 2.56 | 4.29 | - | 1.77 | 3.73 | HSP17.6B |
| Soltu.DM.06G010770 | Peroxidase superfamily protein | 1.51 | 4.04 | 5.78 | - | 3.37 | 4.81 | PER52 |
| Soltu.DM.04G027640 | Peroxidase superfamily protein | 1.26 | - | - | - | - | - | PER12 |
| Soltu.DM.04G037090 | chloroplastic drought-induced stress protein of 32 kD | 1.22 | - | - | - | - | 1.29 | CDSP32 |
| Soltu.DM.12G004810 | catalase | 1.11 | 1.10 | - | - | - | - | CAT2 |

Supplementary Table 7. 16 DEGs inside the response to oxidative stress term (GO:0006979) and only upregulated in the tolerant root in the early response to drought. (-) denotes genes that did not have a significant change from control to the respective time point. Genes referred to in the main text are shown in red font.

| **Potato genome v6.1  Gene ID** | **Potato genome v6.1  Gene Annotation** | **Leaf.Tolerant** | | | **Leaf.Susceptible** | | | **Root.Tolerant** | | | **Root.Susceptible** | | | ***A. thaliana* GeneName** |
| --- | --- | --- | --- | --- | --- | --- | --- | --- | --- | --- | --- | --- | --- | --- |
|  |  | **T1** | **T2** | **T3** | **T1** | **T2** | **T3** | **T1** | **T2** | **T3** | **T1** | **T2** | **T3** |  |
| Soltu.DM.05G023590 | nine-cis-epoxycarotenoid dioxygenase | - | 3.90 | - | - | 3.87 | - | - | 6.80 | 4.01 | - | 7.06 | - | NCED6 |
| Soltu.DM.07G022620 | nine-cis-epoxycarotenoid dioxygenase | - | 3.92 | 4.64 | - | 3.03 | 3.96 | 3.16 | 6.20 | 3.50 | 3.35 | 5.12 | 2.64 | NCED3 |
| Soltu.DM.08G003330 | nine-cis-epoxycarotenoid dioxygenase | - | - | - | - | - | - | - | -3.26 | -2.90 | - | -2.16 | -3.10 | NCED4 |
| Soltu.DM.08G003340 | nine-cis-epoxycarotenoid dioxygenase | - | - | - | - | - | - | - | - | - | - | - | -3.79 | NCED4 |
| Soltu.DM.08G003350 | nine-cis-epoxycarotenoid dioxygenase | - | - | - | - | - | - | - | - | - | - | - | -2.36 | NCED4 |
| Soltu.DM.08G006990 | nine-cis-epoxycarotenoid dioxygenase | - | - | - | - | - | - | 1.86 | 3.74 | 2.53 | 2.23 | 3.79 | 3.46 | NCED5 |
| Soltu.DM.08G015120 | carotenoid cleavage dioxygenase | 1.24 | 1.51 | 1.02 | - | - | - | - | - | - | - | - | - | NCED1 / NCED3 |
| Soltu.DM.08G020360 | nine-cis-epoxycarotenoid dioxygenase | - | - | - | - | - | -1.59 | - | - | - | - | - | - | NCED4 |
| Soltu.DM.08G020370 | nine-cis-epoxycarotenoid dioxygenase | - | - | - | - | -1.44 | -1.35 | - | - | - | - | - | - | NCED4 |

Supplementary Table 8. Expression of NCED genes annotated in the potato genome across time points, tissues, and varieties. Shown is the log2FC from control to each time point. (-) denotes genes that did not have a significant change from control to the respective time point. Genes referred to in the main text are shown in red font.

| **Potato genome v6.1** | **Potato genome v6.1 Gene Annotation** | **Leaf.Tolerant** | | | **Leaf.Susceptible** | | | **Root.Tolerant** | | | **Root.Susceptible** | | | ***A. thaliana*  Gene Name** |
| --- | --- | --- | --- | --- | --- | --- | --- | --- | --- | --- | --- | --- | --- | --- |
|  |  | **T1** | **T2** | **T3** | **T1** | **T2** | **T3** | **T1** | **T2** | **T3** | **T1** | **T2** | **T3** |  |
| Soltu.DM.02G022920 | Chitinase family protein | - | 2.19 | 2.84 | - | - | 2.12 | 1.90 | 2.94 | 3.76 | - | 3.53 | 4.33 | CHI-B |
| Soltu.DM.02G022960 | basic chitinase | *-* | *-* | *-* | *-* | *-* | *-* | 1.61 | 1.73 | - | - | - | - | CHI-B |
| Soltu.DM.07G005390 | basic chitinase | *-* | *-* | *-* | *-* | *-* | *-* | 3.46 | 3.50 | - | - | - | - | CHI-B |
| Soltu.DM.07G005400 | basic chitinase | *-* | *-* | *-* | *-* | *-* | *-* | 3.52 | 3.63 | - | - | - | - | CHI-B |
| Soltu.DM.10G026220 | calmodulin | - | 1.35 | 1.34 | - | 1.23 | 1.59 | - | 1.43 | 1.95 | - | - | 1.15 | CAM7 |
| Soltu.DM.04G012040 | Calcium-binding EF-hand family protein | - | 1.39 | 2.13 | - | - | 1.76 | - | 1.91 | 2.93 | 1.13 | - | 2.30 | CML36 |
| Soltu.DM.10G026210 | calmodulin | - | 1.14 | 1.27 | - | 1.29 | 1.65 | - | 1.21 | 1.72 | - | - | 1.23 | CAM7 |
| Soltu.DM.10G027990 | Calcium-binding EF-hand family protein | - | 1.35 | 1.75 | - | - | 1.70 | - | 0.94 | 1.80 | - | - | 1.45 | CML36 |
| Soltu.DM.09G009490 | WRKY DNA-binding protein | 2.26 | 4.14 | 5.45 | - | - | 4.95 | 1.71 | 1.26 | 3.16 | 1.88 | - | 2.78 | WRKY33 |
| Soltu.DM.08G015910 | WRKY DNA-binding protein | - | 4.19 | 4.16 | - | - | - | - | 2.26 | - | - | - | 1.59 | WRKY40 |
| Soltu.DM.03G030960 | WRKY DNA-binding protein | - | 2.24 | 2.49 | - | - | 2.17 | 2.26 | 2.30 | 6.16 | 3.24 | 2.36 | 6.52 | WRKY40 |
| Soltu.DM.06G018840 | WRKY DNA-binding protein | - | 2.43 | 3.00 | - | - | 2.30 | 1.38 | 1.28 | 2.89 | 1.43 | - | 2.56 | WRKY33 |
| Soltu.DM.09G011140 | WRKY DNA-binding protein | - | 2.43 | 2.68 | - | - | - | - | 2.39 | 1.41 | - | - | - | WRKY70 |
| Soltu.DM.08G012710 | WRKY DNA-binding protein | - | 4.13 | 4.80 | - | 3.59 | 4.34 | 2.03 | 3.77 | 4.41 | - | - | 2.31 | WRKY50 |
| Soltu.DM.06G024270 | WRKY DNA-binding protein | - | 1.62 | 1.86 | - | 1.29 | 2.69 | 1.65 | - | 4.03 | 1.75 | - | 4.18 | WRKY40 |
| Soltu.DM.03G013350 | WRKY DNA-binding protein | - | - | 1.57 | - | - | - | - | 1.86 | 1.63 | - | - | 1.74 | WRKY70 |
| Soltu.DM.04G023540 | WRKY DNA-binding protein | - | 3.96 | 4.53 | - | 2.31 | 1.98 | - | 3.84 | 3.88 | - | 2.55 | 2.37 | WRKY50 |
| Soltu.DM.04G028130 | WRKY DNA-binding protein | - | 2.85 | 3.30 | - | - | 1.71 | - | 3.12 | 4.13 | - | 1.62 | 3.78 | WRKY51 |
| Soltu.DM.08G015900 | WRKY DNA-binding protein | - | 2.23 | 3.32 | - | - | 2.78 | - | 4.71 | 3.91 | - | 2.85 | 3.82 | WRKY40 |
| Soltu.DM.10G018560 | WRKY DNA-binding protein | *-* | *-* | *-* | *-* | *-* | *-* | - | - | - | - | -2.53 | - | WRKY70 |
| Soltu.DM.10G021890 | WRKY DNA-binding protein | *-* | *-* | *-* | *-* | *-* | *-* | - | 3.12 | - | - | - | - | WRKY70 |
| Soltu.DM.12G007400 | WRKY DNA-binding protein | *-* | *-* | *-* | *-* | *-* | *-* | - | 4.31 | 7.68 | - | - | 4.41 | WRKY51 |
| Soltu.DM.12G007390 | WRKY DNA-binding protein | *-* | *-* | *-* | *-* | *-* | *-* | - | - | 6.42 | - | - | 4.10 | WRKY51 |
| Soltu.DM.01G034410 | BCL-2-associated athanogene | 1.95 | 1.65 | - | - | - | - | - | 2.79 | 3.54 | - | 2.67 | 3.07 | BAG6 |

Supplementary Table 9. DEGs annotated as chitinase, calcium interacting proteins, BAG or WRKY genes inside the response to fungus term (GO:0050832). Shown are the log2FC values for genes in GO:0050832 that were DE since T1 or T2 in any tissue or variety. (-) denotes genes that did not have a significant change from control to the respective time point. Genes referred to in the main text are shown in red font.

| **Potato genome v6.1** | **Potato genome v6.1 Gene Annotation** | **Root.Tolerant** | | | **Root.Susceptible** | | |
| --- | --- | --- | --- | --- | --- | --- | --- |
|  |  | **T1** | **T2** | **T3** | **T1** | **T2** | **T3** |
| Soltu.DM.02G021800 | Plant invertase/pectin methylesterase inhibitor superfamily | - | -3.70 | -1.34 | - | -2.88 | -1.58 |
| Soltu.DM.03G015350 | Plant invertase/pectin methylesterase inhibitor superfamily | - | -3.85 | - | - | -2.73 | - |
| Soltu.DM.01G039670 | Plant invertase/pectin methylesterase inhibitor superfamily | - | -5.30 | - | - | -2.50 | -1.90 |
| Soltu.DM.03G015370 | Plant invertase/pectin methylesterase inhibitor superfamily | -0.83 | -2.42 | -0.67 | - | -1.41 | -0.62 |
| Soltu.DM.02G001870 | Plant invertase/pectin methylesterase inhibitor superfamily | - | -4.04 | - | - | - | - |
| Soltu.DM.03G011600 | Plant invertase/pectin methylesterase inhibitor superfamily | - | -1.85 | -1.02 | - | - | -1.16 |
| Soltu.DM.09G023650 | Plant invertase/pectin methylesterase inhibitor superfamily | - | -1.45 | 1.49 | - | - | 1.33 |
| Soltu.DM.06G012610 | Plant invertase/pectin methylesterase inhibitor superfamily | - | -1.71 | - | - | - | - |
| Soltu.DM.05G019240 | Plant invertase/pectin methylesterase inhibitor superfamily | - | -1.70 | -2.27 | - | - | -2.72 |
| Soltu.DM.09G023660 | Plant invertase/pectin methylesterase inhibitor superfamily | - | -1.74 | - | - | - | - |
| Soltu.DM.06G013790 | Plant invertase/pectin methylesterase inhibitor superfamily | - | - | -0.63 | - | -0.59 | -1.06 |
| Soltu.DM.01G031690 | Plant invertase/pectin methylesterase inhibitor superfamily | - | - | - | - | - | -1.15 |
| Soltu.DM.11G008250 | Plant invertase/pectin methylesterase inhibitor superfamily | - | - | 1.73 | - | - | 1.27 |
| Soltu.DM.01G025220 | Pectin lyase-like superfamily protein | - | - | -1.29 | - | -1.76 | -2.23 |
| Soltu.DM.08G024910 | Pectin lyase-like superfamily protein | - | - | -1.54 | - | - | -1.58 |
| Soltu.DM.12G025490 | Pectin lyase-like superfamily protein | - | - | - | - | - | -1.75 |
| Soltu.DM.07G014040 | Pectin lyase-like superfamily protein | - | -1.74 | - | - | - | - |
| Soltu.DM.03G037420 | pectin methylesterase | - | - | 2.13 | - | - | 2.84 |
| Soltu.DM.07G006430 | pectin methylesterase | - | 1.30 | 1.84 | - | 1.34 | 1.85 |
| Soltu.DM.01G049540 | pectinesterase | - | -1.55 | - | - | -0.64 | - |
| Soltu.DM.07G002190 | Xyloglucan endotransglucosylase/hydrolase family protein | - | 3.78 | 4.10 | 1.16 | 4.58 | 4.84 |
| Soltu.DM.12G020770 | Xyloglucan endotransglucosylase/hydrolase family protein | - | 1.62 | 6.65 | - | - | 5.68 |
| Soltu.DM.12G028730 | xyloglucan endotransglucosylase/hydrolase | - | -2.89 | - | - | -2.68 | - |
| Soltu.DM.09G030000 | xyloglucan endotransglucosylase/hydrolase | - | -2.64 | - | - | -2.38 | - |
| Soltu.DM.03G018880 | xyloglucan endotransglucosylase/hydrolase | - | -1.46 | -2.53 | - | -2.27 | -3.19 |
| Soltu.DM.07G021990 | xyloglucan endotransglucosylase/hydrolase | - | -3.09 | 0.87 | - | -1.69 | 0.92 |
| Soltu.DM.03G002910 | xyloglucan endotransglucosylase/hydrolase | - | -2.21 | - | - | -1.63 | - |
| Soltu.DM.12G025130 | xyloglucan endotransglucosylase/hydrolase | - | -3.25 | - | - | -1.59 | - |
| Soltu.DM.10G000430 | xyloglucan endotransglucosylase/hydrolase | - | -1.87 | -2.14 | - | -1.57 | -2.04 |
| Soltu.DM.11G020640 | xyloglucan endotransglucosylase/hydrolase | - | -1.69 | -2.38 | - | -1.56 | -3.02 |
| Soltu.DM.07G018430 | xyloglucan endotransglucosylase/hydrolase | - | - | - | - | -1.55 | - |
| Soltu.DM.10G000420 | xyloglucan endotransglucosylase/hydrolase | - | -1.98 | -1.68 | - | -1.25 | -1.35 |
| Soltu.DM.09G005280 | xyloglucan endotransglucosylase/hydrolase | - | -2.07 | -2.34 | - | -1.48 | -1.26 |
| Soltu.DM.12G025120 | xyloglucan endotransglucosylase/hydrolase | - | -3.60 | - | - | - | - |
| Soltu.DM.07G022400 | xyloglucan endotransglucosylase/hydrolase | 1.59 | - | 5.96 | - | - | 5.25 |
| Soltu.DM.12G028740 | xyloglucan endotransglucosylase/hydrolase | - | -1.88 | 1.87 | - | - | 1.50 |
| Soltu.DM.02G019780 | xyloglucan endotransglucosylase/hydrolase | - | -2.20 | - | - | - | - |
| Soltu.DM.07G002180 | xyloglucan endotransglucosylase/hydrolase | - | -1.02 | 1.08 | - | - | 1.38 |
| Soltu.DM.01G029240 | xyloglucan endotransglucosylase/hydrolase | - | 1.46 | 1.35 | - | 1.46 | 1.74 |
| Soltu.DM.07G005220 | xyloglucan endotransglucosylase/hydrolase | - | - | - | - | 1.51 | 3.07 |
| Soltu.DM.11G019660 | xyloglucan endotransglucosylase/hydrolase | - | 1.91 | 1.74 | - | 1.90 | 1.30 |
| Soltu.DM.11G015160 | xyloglucan endotransglucosylase/hydrolase | - | -1.91 | -1.20 | - | -0.87 | -0.92 |
| Soltu.DM.12G025140 | xyloglucan endotransglucosylase/hydrolase | - | -1.92 | 1.57 | - | - | 2.01 |
| Soltu.DM.07G022000 | xyloglucan endotransglucosylase/hydrolase | - | - | 6.53 | - | - | 5.69 |
| Soltu.DM.12G002500 | xyloglucan endotransglucosylase/hydrolase | - | - | - | - | - | 2.15 |
| Soltu.DM.01G039330 | xyloglucan endotransglucosylase/hydrolase | - | -1.08 | - | - | - | - |
| Soltu.DM.08G021810 | xyloglucan endotransglucosylase/hydrolase | - | - | 1.00 | - | - | 1.63 |
| Soltu.DM.04G003720 | xyloglucan endotransglucosylase/hydrolase | - | - | - | - | - | 1.00 |
| Soltu.DM.05G008140 | xyloglucan:xyloglucosyl transferase | -1.50 | -1.46 | 1.49 | - | - | 1.77 |
| Soltu.DM.03G029870 | xyloglucan xylosyltransferase | - | -0.41 | 1.78 | - | - | 1.50 |
| Soltu.DM.03G015040 | xyloglucan endotransglycosylase | - | -1.94 | 4.00 | -1.28 | -3.31 | 3.57 |
| Soltu.DM.03G015050 | xyloglucan endotransglycosylase | - | - | 5.36 | - | -2.59 | 4.83 |
| Soltu.DM.03G015060 | xyloglucan endotransglycosylase | - | -2.04 | 4.77 | - | -2.52 | 4.32 |
| Soltu.DM.03G015070 | xyloglucan endotransglycosylase | - | -1.63 | 4.61 | - | -2.41 | 3.98 |
| Soltu.DM.05G018970 | xyloglucan endotransglycosylase | - | - | 1.99 | - | - | 1.83 |
| Soltu.DM.09G023860 | cellulose synthase-like D5 | - | -1.00 | -0.84 | - | - | -1.93 |
| Soltu.DM.05G000310 | Exostosin family protein | - | - | - | - | -1.48 | -1.42 |
| Soltu.DM.04G036240 | glyoxal oxidase-related protein | - | -2.17 | -1.49 | - | -1.31 | -1.24 |
| Soltu.DM.04G036230 | glyoxal oxidase-related protein | - | -1.48 | - | - | - | - |
| Soltu.DM.04G036250 | glyoxal oxidase-related protein | - | -2.09 | - | - | - | - |
| Soltu.DM.04G034500 | OBF binding protein | - | - | -1.29 | - | -0.85 | -1.67 |
| Soltu.DM.02G022030 | Domain of unknown function (DUF23) | - | - | 1.07 | - | -0.76 | 0.68 |
| Soltu.DM.08G030280 | pyrophosphorylase | - | -0.99 | 1.34 | - | - | 1.47 |
| Soltu.DM.12G008450 | UDP-D-glucose/UDP-D-galactose 4-epimerase | -0.89 | -1.78 | 0.58 | -0.71 | - | 2.00 |
| Soltu.DM.01G022940 | UDP-xylosyltransferase | - | - | 2.26 | - | 0.47 | 2.22 |
| Soltu.DM.05G000160 | Galactosyl transferase GMA12/MNN10 family protein | - | -2.17 | -1.86 | - | - | -1.42 |
| Soltu.DM.01G003570 | TRICHOME BIREFRINGENCE-LIKE | - | - | 1.19 | - | - | - |
| Soltu.DM.07G025630 | methylesterase PCR A | - | - | - | - | - | 1.29 |
| Soltu.DM.08G027650 | root hair specific | - | 1.64 | 2.07 | - | 0.98 | 1.80 |
| Soltu.DM.02G009560 | conserved hypothetical protein | - | -3.74 | - | - | - | -2.76 |

Supplementary Table 10. Genes inside the GO terms related to the cell wall (xyloglucan metabolic process: GO:0010411, cell wall biogenesis: GO:0042546 and cell wall modification: GO:0042545 that were enriched in the late response of the tolerant variety. Log2FC values are shown. (-) denotes genes that did not have a significant change from control to the respective time point. Genes referred to in the main text are shown in red font.

**(A)**

| **Potato genome v6.1**  **Gene ID** | **Potato genome v6.1 Gene Annotation** | **Leaf.Tolerant** | | | | | | **Leaf.Susceptible** | | | | | |
| --- | --- | --- | --- | --- | --- | --- | --- | --- | --- | --- | --- | --- | --- |
|  |  | **T1** | | **T2** | | **T3** | | **T1** | | **T2** | | **T3** | |
|  |  | **LFC** | **padj.** | **LFC** | **padj.** | **LFC** | **padj.** | **LFC** | **padj.** | **LFC** | **padj.** | **LFC** | **padj.** |
| **Upregulated genes from control to T1** | | | | | | | | | | | | | |
| **Cell wall modification** | | | | | | | | | | | | | |
| Soltu.DM.03G015500* | Plant invertase/pectin methylesterase inhibitor superfamily protein | 3.17 | 3.32E-05 | 4.51 | 1.20E-12 | 3.71 | 1.76E-07 | 1.78 | 4.60E-01 | 5.16 | 1.01E-12 | 5.71 | 6.09E-19 |
| Soltu.DM.03G015510* | Plant invertase/pectin methylesterase inhibitor superfamily protein | 2.9 | 5.30E-09 | 5.13 | 8.02E-32 | 4.56 | 2.29E-20 | 1.98 | 3.29E-03 | 5.55 | 1.59E-28 | 6.08 | 2.12E-42 |
| **Chaperones** | | | | | | | | | | | | | |
| Soltu.DM.06G031870* | HSP20-like chaperones superfamily protein | 5.45 | 2.74E-02 | 5.9 | 2.10E-03 | 4.68 | 3.17E-02 | -0.06 | 1.00E+00 | 6.15 | 4.80E-03 | 5.51 | 2.59E-03 |
| **Transcription factor** | | | | | | | | | | | | | |
| Soltu.DM.06G015000 | heat shock transcription factor A6B | 2.42 | 3.37E-03 | 3.42 | 1.23E-07 | 2.65 | 3.19E-04 | 1.57 | 5.06E-01 | 3.38 | 3.40E-06 | 2.93 | 3.04E-06 |
| **Pathogen response** | | | | | | | | | | | | | |
| Soltu.DM.12G005300 | bifunctional nuclease in basal defense response | 2.28 | 2.54E-02 | 2.5 | 1.62E-03 | 1.39 | 1.44E-01 | 0.31 | 1.00E+00 | 1 | 3.58E-01 | 1.54 | 5.35E-02 |
| **Starch breakdown** | | | | | | | | | | | | | |
| Soltu.DM.08G023420* | chloroplast beta-amylase | 2.4 | 1.09E-06 | 2.86 | 3.65E-11 | 2.11 | 2.24E-05 | 0.39 | 1.00E+00 | 1.78 | 6.95E-04 | 1.86 | 2.24E-05 |
| **Transport** | | | | | | | | | | | | | |
| Soltu.DM.04G031660 | MATE efflux family protein | 2.2 | 7.60E-03 | 2.3 | 4.84E-04 | 1.34 | 8.62E-02 | 0.73 | 1.00E+00 | 1.38 | 8.91E-02 | 1.6 | 1.31E-02 |
| **Calcium binding** | | | | | | | | | | | | | |
| Soltu.DM.01G032110* | EF hand calcium-binding protein family | 1.3 | 3.09E-02 | 1.92 | 1.20E-05 | 1.25 | 1.26E-02 | 0.03 | 1.00E+00 | 0.74 | 2.44E-01 | 1 | 3.66E-02 |
| **Others** | | | | | | | | | | | | | |
| Soltu.DM.08G010200* | cytochrome P450, family 71, subfamily B, polypeptide | 4.18 | 1.53E-03 | 4.52 | 3.17E-05 | 3.5 | 4.35E-03 | 1.09 | 1.00E+00 | 3.28 | 2.55E-02 | 3.19 | 1.03E-02 |
| Soltu.DM.01G028100* | beta glucosidase | 1.43 | 1.02E-02 | 1.54 | 5.25E-04 | 0.85 | 1.10E-01 | 0 | 1.00E+00 | 0.13 | 8.70E-01 | -0.18 | 7.46E-01 |
| Soltu.DM.12G009420 | Cytochrome P450 superfamily protein | 2.47 | 3.41E-02 | 2.5 | 5.73E-03 | 1.49 | 1.65E-01 | 0.25 | 1.00E+00 | 0.69 | 6.03E-01 | 0.59 | 5.67E-01 |
| Soltu.DM.04G034610* | cytochrome P450, family 82, subfamily C, polypeptide | 1.71 | 9.32E-04 | 1.86 | 1.32E-05 | 1.23 | 1.39E-02 | 0.5 | 1.00E+00 | 1.62 | 1.22E-03 | 1.77 | 2.12E-05 |
| Soltu.DM.09G002950 | 2-oxoglutarate (2OG) and Fe(II)-dependent oxygenase superfamily protein | 2.22 | 1.88E-02 | 2.62 | 3.04E-04 | 1.51 | 7.76E-02 | 0.55 | 1.00E+00 | 0.8 | 3.87E-01 | 1.04 | 1.30E-01 |
| Soltu.DM.09G002960 | 2-oxoglutarate (2OG) and Fe(II)-dependent oxygenase superfamily protein | 1.97 | 1.47E-02 | 2.41 | 1.08E-04 | 1.55 | 3.28E-02 | 0.04 | 1.00E+00 | 0.4 | 6.93E-01 | 1.09 | 9.27E-02 |
| Soltu.DM.09G002970 | 2-oxoglutarate (2OG) and Fe(II)-dependent oxygenase superfamily protein | 1.82 | 8.93E-04 | 2.71 | 3.57E-10 | 2.13 | 1.20E-05 | -0.33 | 1.00E+00 | 0.24 | 7.19E-01 | 0.83 | 5.15E-02 |
| Soltu.DM.04G007210* | alpha/beta-Hydrolases superfamily protein | 2 | 3.69E-02 | 3.63 | 1.06E-07 | 1.34 | 1.22E-01 | 0.56 | 1.00E+00 | 2.58 | 1.87E-03 | 3.45 | 3.28E-07 |
| Soltu.DM.01G026800 | Subtilase family protein | 1.85 | 2.38E-03 | 2.81 | 3.08E-09 | 2.01 | 2.29E-04 | 0.35 | 1.00E+00 | 1.19 | 4.87E-02 | 0.96 | 5.81E-02 |
| Soltu.DM.01G050660 | GAST1 protein homolog | 1.82 | 4.70E-02 | 2.03 | 3.57E-03 | 1.46 | 6.90E-02 | -0.15 | 1.00E+00 | -0.18 | 8.85E-01 | 0.75 | 3.17E-01 |
| Soltu.DM.02G020310 | deoxyhypusine synthase | 2.39 | 4.28E-02 | 2.96 | 6.83E-04 | 2.31 | 1.67E-02 | 0.19 | NA | 0.63 | 5.91E-01 | 0.94 | 2.65E-01 |
| Soltu.DM.03G029920 | Tetratricopeptide repeat (TPR)-like superfamily protein | 1.21 | 2.40E-02 | 1.48 | 2.53E-04 | 0.96 | 3.56E-02 | 0.16 | 1.00E+00 | 1.29 | 2.13E-03 | 1.3 | 2.62E-04 |
| Soltu.DM.01G040460 | hypothetical protein | 1.54 | 4.29E-02 | 2.3 | 2.75E-05 | 1.56 | 1.38E-02 | 0.29 | 1.00E+00 | 2.08 | 5.94E-04 | 1.32 | 1.42E-02 |
| **Downregulated genes from control to T1** | | | | | | | | | | | | | |
| **Transcription factors** | | | | | | | | | | | | | |
| Soltu.DM.09G019660 | basic helix-loop-helix (bHLH) DNA-binding superfamily protein | -1.5 | 4.60E-02 | -1.8 | 1.56E-03 | -0.39 | 6.13E-01 | -0.36 | 1.00E+00 | -0.09 | 9.31E-01 | -0.15 | 8.40E-01 |
| **Others** | | | | | | | | | | | | | |
| Soltu.DM.10G000510 | terpene synthase | -1.75 | 8.65E-03 | -2.54 | 7.95E-07 | -0.8 | 2.31E-01 | -0.29 | 1.00E+00 | -1.9 | 2.71E-03 | -3.55 | 2.97E-11 |
| Soltu.DM.08G002160* | FAD-dependent oxidoreductase family protein | -1.48 | 1.94E-03 | -1.93 | 1.19E-06 | -1.2 | 5.58E-03 | 0.17 | 1.00E+00 | -0.58 | 2.66E-01 | -0.66 | 9.92E-02 |
| Soltu.DM.02G024270* | B-box type zinc finger family protein | -1.47 | 1.72E-02 | -4.35 | 2.05E-19 | -3.82 | 2.88E-13 | -0.6 | 1.00E+00 | -3.96 | 1.01E-13 | -4.89 | 1.94E-24 |

**(B)**

| **Potato genome v6.1**  **Gene ID** | **Potato genome v6.1 Gene Annotation** | **Root.Tolerant** | | | | | | **Root.Susceptible** | | | | | |
| --- | --- | --- | --- | --- | --- | --- | --- | --- | --- | --- | --- | --- | --- |
|  |  | **T1** | | **T2** | | **T3** | | **T1** | | **T2** | | **T3** | |
|  |  | **LFC** | **padj.** | **LFC** | **padj** | **LFC** | **padj** | **LFC** | **padj.** | **LFC** | **padj** | **LFC** | **padj** |
| **Upregulated genes from control to T1** | | | | | | | | | | | | | |
| **Transcription factors** | | | | | | | | | | | | | |
| Soltu.DM.04G000840 | NAC-like, activated by AP3/PI | 3.78 | 4.64E-06 | 5.19 | 4.94E-12 | 3.96 | 1.57E-08 | 2.08 | 5.53E-03 | 3.82 | 2.15E-10 | 2.46 | 5.93E-05 |
| Soltu.DM.07G014750 | NAC domain containing protein | 3.55 | 1.87E-02 | 4.43 | 2.67E-04 | 2.64 | 3.02E-02 | -0.12 | NA | 1.04 | 3.67E-01 | -0.46 | 7.06E-01 |
| Soltu.DM.10G000020 | NAC domain containing protein | 2.31 | 4.74E-02 | 2.65 | 3.95E-03 | 1.38 | 1.45E-01 | 1.26 | NA | 0.9 | 3.62E-01 | -1.33 | 1.86E-01 |
| Soltu.DM.01G044140 | xylem NAC domain | 1.44 | 4.01E-02 | 2.62 | 1.53E-06 | 1.4 | 6.23E-03 | 0.63 | 3.87E-01 | 1.61 | 1.75E-03 | 0.8 | 1.33E-01 |
| Soltu.DM.02G018840 | MYB-like | 3.32 | 1.93E-03 | 6.22 | 2.49E-11 | 3.94 | 2.67E-06 | 1.91 | 9.09E-02 | 4.44 | 1.74E-07 | 2.72 | 1.57E-03 |
| Soltu.DM.07G019030 | MYB-like | 2.11 | 8.09E-03 | 5.61 | 2.31E-18 | 4.31 | 7.81E-14 | 1.31 | 9.54E-02 | 3.28 | 1.90E-08 | 3.31 | 5.37E-09 |
| Soltu.DM.01G040220 | basic region/leucine zipper motif | 1.45 | 2.46E-02 | 1.92 | 2.85E-04 | 1.19 | 1.68E-02 | -0.15 | 8.60E-01 | 1.93 | 1.06E-05 | 1.57 | 2.74E-04 |
| **Pathogen response** | | | | | | | | | | | | | |
| Soltu.DM.07G005400* | basic chitinase | 3.52 | 3.12E-04 | 3.63 | 8.11E-05 | 1.25 | 1.75E-01 | -0.17 | 9.16E-01 | 1.17 | 2.52E-01 | -0.54 | 5.97E-01 |
| Soltu.DM.07G005390* | basic chitinase | 3.46 | 1.03E-03 | 3.5 | 4.17E-04 | 1.31 | 1.77E-01 | -0.25 | 8.82E-01 | 0.82 | 4.93E-01 | -0.73 | 4.81E-01 |
| Soltu.DM.02G022960* | basic chitinase | 1.61 | 1.37E-04 | 1.73 | 1.61E-05 | 0.53 | 1.89E-01 | 0.24 | 6.94E-01 | -0.33 | 5.30E-01 | -0.21 | 6.50E-01 |
| Soltu.DM.02G033060* | beta-1,3-glucanase | 3.14 | 3.43E-06 | 3.73 | 1.08E-08 | 1.21 | 6.55E-02 | -0.49 | 6.17E-01 | -0.59 | 4.95E-01 | -2.96 | 2.04E-05 |
| Soltu.DM.03G017710 | PAR1 protein | 2.16 | 1.24E-02 | 3.03 | 3.44E-05 | 0.73 | 3.69E-01 | 0.14 | NA | 1.45 | 4.29E-02 | -0.05 | 9.60E-01 |
| Soltu.DM.09G027690 | MLP-like protein | 2.91 | 2.20E-05 | 3.26 | 8.56E-07 | 2.21 | 1.89E-04 | 0.83 | 3.36E-01 | 1.39 | 3.82E-02 | 1.23 | 5.04E-02 |
| **Transport** | | | | | | | | | | | | | |
| Soltu.DM.06G030890 | nitrate excretion transporter1 | 1.96 | 4.89E-02 | 2.12 | 8.61E-03 | 1.13 | 1.65E-01 | 0.38 | NA | 0.47 | 6.52E-01 | 0.62 | 4.54E-01 |
| Soltu.DM.04G001100 | nitrate transporter 1:2 | 1.52 | 2.36E-02 | 2.88 | 9.88E-08 | 1.74 | 4.19E-04 | 0.61 | 3.95E-01 | 1.52 | 3.34E-03 | 1.47 | 2.57E-03 |
| **Signalling cascade** | | | | | | | | | | | | | |
| Soltu.DM.07G017180 | mitogen-activated protein kinase kinase kinase | 1.51 | 1.18E-02 | 4.79 | 2.77E-28 | 3.02 | 3.48E-13 | 0.56 | 3.78E-01 | 3.59 | 1.95E-21 | 2.25 | 7.99E-09 |
| **Others** | | | | | | | | | | | | | |
| Soltu.DM.05G002810 | alpha/beta-Hydrolases superfamily protein | 5.59 | 1.68E-04 | 6.07 | 9.85E-06 | 5.13 | 2.81E-05 | 3.55 | 9.58E-03 | 2.78 | 2.82E-02 | 4.37 | 7.78E-05 |
| Soltu.DM.09G019250 | EID1-like | 5.51 | 5.73E-08 | 8.07 | 3.01E-16 | 6.5 | 1.33E-13 | 3.99 | 1.44E-04 | 7.2 | 2.38E-16 | 5.62 | 1.41E-10 |
| Soltu.DM.01G044850 | RING/U-box superfamily protein | 1.2 | 4.48E-02 | 1.35 | 6.25E-03 | 0.84 | 6.62E-02 | -0.07 | 9.32E-01 | -0.03 | 9.74E-01 | -0.45 | 3.47E-01 |
| Soltu.DM.06G012790* | 2-oxoglutarate (2OG) and Fe(II)-dependent oxygenase superfamily protein | 2.64 | 1.38E-02 | 5.39 | 6.80E-12 | 3.84 | 5.45E-07 | 0.9 | NA | 3.59 | 6.90E-06 | 2.98 | 1.67E-04 |
| Soltu.DM.11G010780 | 2-oxoglutarate (2OG) and Fe(II)-dependent oxygenase superfamily protein | 2.27 | 3.93E-05 | 2.74 | 7.52E-08 | 2.2 | 2.08E-06 | 0.52 | 4.18E-01 | 0.57 | 3.07E-01 | 0.38 | 4.63E-01 |
| Soltu.DM.04G021610 | NAD(P)-binding Rossmann-fold superfamily protein | 2.59 | 3.59E-03 | 3.08 | 1.28E-04 | 2.55 | 2.93E-04 | 1.05 | 2.96E-01 | 1.36 | 9.91E-02 | 0.15 | 8.77E-01 |
| Soltu.DM.08G030040 | homeobox | 2.33 | 1.19E-04 | 6.03 | 1.71E-30 | 4.75 | 7.95E-24 | 1.27 | 5.11E-02 | 5.26 | 5.19E-29 | 3.73 | 3.91E-15 |
| Soltu.DM.05G006430 | homeobox | 1.6 | 2.95E-04 | 4.11 | 5.91E-27 | 2.43 | 2.72E-12 | -0.03 | 9.74E-01 | 2.38 | 1.08E-11 | 0.7 | 7.07E-02 |
| Soltu.DM.03G030760 | blue-copper-binding protein | 1.85 | 2.60E-02 | 1.93 | 6.64E-03 | 0.73 | 3.27E-01 | 0.83 | NA | 1.34 | 5.78E-02 | 1.8 | 3.61E-03 |
| Soltu.DM.02G018100 | serine carboxypeptidase-like | 1.8 | 1.86E-03 | 2.87 | 1.86E-08 | 1.44 | 2.35E-03 | 0.42 | 5.56E-01 | -0.57 | 3.40E-01 | -0.6 | 2.44E-01 |
| Soltu.DM.01G026820 | Subtilase family protein | 1.69 | 4.93E-03 | 2.56 | 3.16E-07 | 0.82 | 1.32E-01 | 0.81 | NA | 0.56 | 3.85E-01 | -0.55 | 3.63E-01 |
| Soltu.DM.01G043360 | plantacyanin | 1.16 | 1.27E-03 | 1.3 | 1.06E-04 | 0.46 | 1.60E-01 | -0.13 | 8.03E-01 | -0.4 | 2.92E-01 | -0.32 | 3.53E-01 |
| Soltu.DM.06G015140 | Protein of unknown function (DUF1677) | 2.73 | 1.31E-03 | 4.25 | 1.74E-09 | 3.01 | 7.33E-06 | 1.81 | NA | 3.94 | 7.57E-09 | 2.59 | 2.44E-04 |
| Soltu.DM.10G027280* | Protein of unknown function, DUF617 | 2.72 | 1.70E-07 | 6.01 | 2.09E-41 | 4.9 | 5.93E-31 | 1.71 | 2.45E-03 | 5.2 | 1.30E-35 | 3.76 | 1.04E-18 |
| Soltu.DM.09G020460 | Protein of unknown function (DUF506) | 1.52 | 1.19E-02 | 2.69 | 1.02E-07 | 1.36 | 3.61E-03 | 0.71 | 2.74E-01 | 2.18 | 1.89E-06 | 1.62 | 3.48E-04 |
| Soltu.DM.03G024470 | conserved hypothetical protein | 4.04 | 2.30E-04 | 8.2 | 2.01E-17 | 5.86 | 1.33E-11 | 2.3 | 4.59E-02 | 7.03 | 2.03E-16 | 4.25 | 1.03E-06 |
| Soltu.DM.06G024460 | conserved hypothetical protein | 2.85 | 1.87E-09 | 3.9 | 8.68E-17 | 0.54 | 3.15E-01 | 1.67 | 9.59E-04 | 3.05 | 3.32E-13 | 0.14 | 8.08E-01 |
| Soltu.DM.03G025840 | hypothetical protein | 2.6 | 2.65E-03 | 4.32 | 3.54E-09 | 3.07 | 4.76E-06 | 1.33 | 1.22E-01 | 2.13 | 1.73E-03 | 2.31 | 2.71E-04 |
| Soltu.DM.05G002980 | hypothetical protein | 2.53 | 1.37E-02 | 3.54 | 5.99E-05 | 2.72 | 4.70E-04 | 0.76 | 5.30E-01 | 2.43 | 3.45E-03 | 2.78 | 3.04E-04 |
| Soltu.DM.10G026720 | conserved hypothetical protein | 2.26 | 2.40E-02 | 3 | 2.02E-04 | 1.3 | 1.24E-01 | 1.33 | NA | 2.12 | 4.54E-03 | 1.5 | 4.51E-02 |
| Soltu.DM.02G033970 | conserved hypothetical protein | 1.69 | 1.37E-02 | 4.79 | 6.81E-19 | 4.21 | 1.99E-18 | 0.76 | 2.88E-01 | 3.45 | 6.77E-13 | 2.38 | 8.91E-07 |
| Soltu.DM.12G003800* | conserved hypothetical protein | 1.32 | 4.03E-02 | 1.46 | 7.03E-03 | 0.11 | 8.66E-01 | 0.47 | 5.17E-01 | 0.78 | 1.69E-01 | 0.9 | 7.10E-02 |
| **Downregulated genes from control to T1** | | | | | | | | | | | | | |
| **Flavonoid synthesis** | | | | | | | | | | | | | |
| Soltu.DM.02G023850 | flavanone 3-hydroxylase | -2.15 | 5.06E-04 | -2.85 | 1.23E-05 | -0.72 | 1.58E-01 | -0.92 | NA | -0.23 | 7.40E-01 | 0.68 | 1.49E-01 |
| **Others** | | | | | | | | | | | | | |
| Soltu.DM.01G049280* | conserved hypothetical protein | -2.75 | 1.60E-02 | -3.37 | 7.92E-04 | -0.76 | 4.67E-01 | -0.84 | 5.18E-01 | -1.46 | 1.58E-01 | -1.98 | 2.44E-02 |
| Soltu.DM.10G000080* | circadian clock associated | -1.92 | 1.24E-03 | -4.9 | 1.40E-12 | -3.96 | 3.85E-14 | -0.57 | 4.18E-01 | -2.76 | 1.90E-08 | -2.71 | 1.59E-08 |
| Soltu.DM.02G027940 | Protein of unknown function (DUF1230) | -0.95 | 3.28E-02 | -1.52 | 4.12E-05 | -0.87 | 8.73E-03 | 0.16 | 7.81E-01 | -0.41 | 3.31E-01 | -0.08 | 8.60E-01 |

Supplementary Table 11. Genes responding rapidly to drought and rewatering in the tolerant variety. Log2FC values are shown for both varieties in leaf (A) and root (B), with values colour coded from high (red) to low (yellow). padj > 0.05 are shown in grey font. An asterisk denotes genes whose expression was significantly higher (if upregulated from control to T1) or lower (if downregulated from control to T1) in the tolerant than in the susceptible during T1. Genes referred to in the main text are shown in red font.
